# Optimal estimation of drift and diffusion coefficients in the presence of static localization error

**DOI:** 10.1103/PhysRevE.100.022134

**Published:** 2019-08-30

**Authors:** J. Devlin, D. Husmeier, J. A. Mackenzie

**Affiliations:** 1 Department of Mathematics and Statistics, University of Strathclyde, Livingstone Tower, Glasgow G1 1XH, United Kingdom; 2 School of Mathematics and Statistics, University of Glasgow, Glasgow G12 8QQ, United Kingdom

## Abstract

We consider the inference of the drift velocity and the diffusion coefficient of a particle undergoing a directed random walk in the presence of static localization error. A weighted least-squares fit to mean-square displacement (MSD) data is used to infer the parameters of the assumed drift-diffusion model. For experiments which cannot be repeated we show that the quality of the inferred parameters depends on the number of MSD points used in the fitting. An optimal number of fitting points *p*_opt_ is shown to exist which depends on the time interval between frames Δ*t* and the unknown parameters. We therefore also present a simple iterative algorithm which converges rapidly toward *p*_opt_. For repeatable experiments the quality depends crucially on the measurement time interval over which measurements are made, reflecting the different timescales associated with drift and diffusion. An optimal measurement time interval *T*_opt_ exists, which depends on the number of measurement points and the unknown parameters, and so again we present an iterative algorithm which converges quickly toward *T*_opt_ and is shown to be robust to initial parameter guesses.

## I Introduction

Understanding the properties and dynamics of moving particles is of primary interest in a variety of disciplines. Examples include the study of cell movement in cell biology [1,2], elucidating the driving forces of metastasis in cancer research [3], understanding the causes of animal mass migration in ecology [4], monitoring crowd behavior in social science [5,6], and studying rumour diffusion in social networks [7].

Typically, the position of a particle is extracted from a sequence of digital images. The measured trajectory is the path observed using a device such as a microscope connected to a video camera. The measured trajectory can be subject to two different types of localization error, usually referred to as static error and dynamic error [8]. Static error is the difference between the measured and true position of an immobile particle or the instantaneous position of a moving particle. The source of static error therefore comes from the spatial resolution of the measuring instrument. Dynamic errors are inaccuracies which arise when measuring particles which move in time. An example of dynamic error is motion blur which can occur due to the camera shutter being left open to maximize the number of photons being recorded in any one frame. For transport by pure diffusion it has been shown [9] that the precision of determining the diffusion constant is negligibly effected by motion blur and hence in the main paper we will assume that it can be ignored (we do present some numerical simulations of motion blur in Sec. 6 of the Supplemental Material to investigate the effect of this assumption). We will, however, include the effects of static error in the calculations which follow.

The analysis of the resulting trajectory data has traditionally been obtained using the mean-square displacement (MSD) [8,10–12]. Recently it has been recognized that the quality of the statistical inference of diffusion coefficients from realistic particle data is nontrivial. Qian *et al.* [10] were first to consider this question for a drift-diffusion model in an isotropic medium. Their analysis, however, did not consider the more practically relevant situation where static error is present in the data collection. The effect of static error on the quality of inference of diffusion coefficients using MSD analysis was addressed by Michalet [13]. Estimates of the MSD at any given time point were obtained using time-averaged quantities which makes the analysis nontrivial due to the nonuniform variance in the MSD data as well as the data being highly correlated. Michalet considered the uncertainty in the estimation of the diffusion coefficient and static error using weighted and ordinary least-squares regression. Due to the heteroscedasticity of the MSD data one would expect the weighted least-squares (WLS) approach to outperform ordinary least squares. This in general is shown to be true if no consideration is given to the number of MSD data points used in the fitting [12,13]. However, the analysis of the uncertainty in the regression coefficients allows the identification of an optimal number of fitting points which depends on the total number of measurement points as well as the control parameter *x* = *η*^2^/*D*Δ*t*, where *η* is the standard deviation of static error, Δ*t* is the frame duration or time between measurements, and *D* is the diffusion coefficient. Specifically, for a small value of *x*, corresponding to a large value for the diffusion coefficient or the time lag, the best estimate of the diffusion coefficient was found by using the first two MSD points; while for a large value of *x* the best estimate was obtained by using a larger number of points. Surprisingly, Michalet found that if the number of fitting points was optimized using weighted and ordinary least squares, then there was very little difference in the optimal level of uncertainty in the parameters.

A related paper was published around the same time by Berglund [14], who proposed the use of a maximum likelihood estimator (MLE) to infer the diffusion coefficient, also for single particles undergoing Brownian motion in the presence of static error. Following this work, Michalet and Berglund [15] provided theoretical Cramér-Rao lower bounds (CRLB) for the uncertainties in the estimates of both parameters. Furthermore, they showed through simulations that the CRLB was attained using an MLE estimator as well as the optimized least-squares approach using ordinary least squares with the optimal number of fitting points. More recently, Vestergaard [9] considered the use of a simple covariance-based estimator (CVE) and experimental protocols for the determination of parameters for pure diffusion and showed that the CVE performed well in comparison to the CRLB.

The use of MSD has also been used for other models of particle transport. Savin and Doyle [8] derived a general formula for the MSD in the prescence of both static and dynamic errors, for any type of particle motion, and used this to study Maxwell and Voigt models of viscoelastic materials. Shanbhag [16] also looked at the determination of the diffusion coefficient for systems where long time diffusive behavior is preceeded by a short time nondiffusive behavior. A simple measure of the local curvature of the MSD curve was used to determine the nondiffusive regime which was then excluded from the fitting process used to determine the diffusion constant. For a persistent random migation model for self-propelled particles, Tang and Underhill [17] showed that accuracy and precision of the parameters defining the model depended on the timescale over which the MSD was fitted and that this should include the transition region from ballistic to diffusive behavior.

In this paper we extend the analysis of Michalet [13] to particles undergoing drift as well as diffusion in the presence of static error. Drift-diffusion or biased random-walk models have been used in many areas particularly in biology, for example, in the detection of biased motion of leukocytes [18] and T cells [19] and in animal movement [4]. The inclusion of drift gives rise to two timescales associated with the diffusive and transport processes, making the optimal determination of the model parameters more difficult compared to the diffusion only case. Qian *et al.* [10] looked at the variance present in the estimation of the MSD in a diffusion only model and the limit that this would impose on the detection of a drift velocity if the MSD curve was fitted by a quadratic polynomial. This study, however, did not explicitly look at the uncertainties in parameter estimations obtained from fitting the MSD to data from a drift-diffusion model with static error. Saxton [20] used the radius of gyration tensor in an attempt to measure the asymmetry of measured particle trajectories to determine the presence of directional bias. This work, however, did not consider the effect of static error or a quantification of the drift-diffusion model parameters. Here we show that by using weighted least-squares quadratic regression to fit the ensemble time-average MSD curve, the diffusion coefficient, drift magnitude, and strength of the static error can be estimated. This can be done in two different ways depending on whether the experimental data can be recollected. If experiments cannot be repeated, following the work of Michalet [13], then an optimal number of fitting points can be found to best infer the parameters with the data at hand. If repeating experiments is possible, then an optimal measurement time interval is shown to exist which minimizes the uncertainty in inferring the parameters when using WLS on all the MSD points. Both quantities depend on the model parameters themselves and so iterative algorithms are presented for both approaches to obtain an estimate of the optimal number of fitting points and the optimal measurement time interval, along with estimates of the parameters in each. The cases of nonisotropic media and where the particles undergo multiple types of diffusion will not be considered in this paper. All mathematical derivations will be provided in the Supplemental Material [21].

The layout of the rest of the paper is as follows. In Sec. II we introduce the stochastic drift-diffusion equation (SDE) that the particles are assumed to follow and calculate a theoretical expression for the mean and variance of the squared displacement and variance of the MSD. The parameters will be estimated using weighted least-squares regression and so expressions for the variance of the regression coefficients and the covariance of the MSD are presented. In Sec. III we present the results for nonrepeatable experiments, including the estimation of the optimal number of fitting points and use of an iterative algorithm to estimate the model parameters. Similar results for repeatable experiments, including estimating the optimal measurement time interval and the corresponding iterative algorithm, are presented in Sec. IV. A discussion of the use of the results in this paper is given in Sec. V and conclusions are given in Sec. VI.

## II Stochastic Drift-Diffusion Model

We will assume that all the particles move in two dimensions. The true location of a particle at time *t* will be denoted by the random variable ${\tilde{\text{X}}}_{t}$ and it will be assumed that it evolves according to the drift-diffusion SDE,

(1) $$d{\tilde{\text{X}}}_{t}=\alpha dt+\sqrt{2D}d{\text{W}}_{t}.$$

The drift velocity ***α*** = *α*[cos(*θ_d_*), sin(*θ_d_*)], where *α* is the drift magnitude and *θ_d_* is the drift direction; for simplicity we assume that *α* and *θ_d_* are fixed so do not depend on time. The diffusion coefficient is denoted by *D* and *d**W**_t_* = (*dW*_1_, *dW*_2_), where *dW*_1,2_ are independent Wiener processes. We will assume that the measured position of a particle is subject to additive independent and identically distributed static error of the form *𝒩*(**0**, *η*^2^*I*), where *η*^2^ is the variance of the static error and *I* is the identity matrix. Throughout this paper we assume that the static error is independent of time. Note that we do not consider experimental factors which affect the level of static error such as finite frame duration and pixelization of video images; the interested reader can find these issues addressed in Savin and Doyle [8].

### A The mean-squared displacement curve

Assuming that particles follow the drift-diffusion equation (1), the probability density function (PDF) for their displacement at time *t* is given by [22]

$$\tilde{p}\left(\tilde{\text{x}},t\right)=\frac{1}{4\pi Dt}\;\text{exp}\left(\frac{-{\left|\tilde{\text{x}}-\alpha t\right|}^{2}}{4Dt}\right).$$

The observed displacement of a particle from the origin at time *t* will be denoted by the random variable ${\text{X}}_{t}^{o}.$ Since ${\text{X}}_{t}^{o}={\tilde{\text{X}}}_{t}+\text{Z},$ where ***Z*** is the random variable denoting the static error with PDF,

$${\tilde{p}}^{n}\left(z\right)=\frac{1}{2\pi \eta^{2}}\;\text{exp}\left({\frac{-\left|z\right|}{2\eta^{2}}}^{2}\right),$$

then the PDF of ${\text{X}}_{t}^{o}$ can be shown to be

$$p^{o}\left({\text{x}}^{o},t\right)=\frac{1}{2\pi \left(2Dt+\eta^{2}\right)}\;\text{exp}\left[\frac{-{\left|{\text{x}}^{o}-\alpha t\right|}^{2}}{2\left(2Dt+\eta^{2}\right)}\right].$$

The measured displacements of the particles are made relative to the origin with the addition of static error. If ***X**_t_* denotes the random variable for the measured displacement, then ${\text{X}}_{t}={\text{X}}_{t}^{o}-Z,$ and hence its PDF is given by

(2) $$p\left(\text{x},t\right)=\frac{1}{2\pi \left(2Dt+2\eta^{2}\right)}\;\text{exp}\left[\frac{-\left|\text{x}-\alpha t^{2}\right|}{2\left(2Dt+2\eta^{2}\right)}\right].$$

The measured MSD is defined as

$$\rho \left(t\right)\equiv 𝔼\left({\left|{\text{X}}_{t}\right|}^{2}\right)=\int_{{\mathbb{R}}^{2}}{\left|\text{x}\right|}^{2}p\left(\text{x},t\right)d\text{x}.$$

Using the PDF for the observed displacement (2) it can be shown (see Supplemental Material, section 1) that

(3) $$\rho \left(t\right)=\alpha^{2}t^{2}+4Dt+4\eta^{2}.$$

This result has been derived previously without the inclusion of static error; for example, by Qian *et al.* [10] and Codling *et al.* [22]. Note that *ρ*(*t*) is independent of the drift angle *θ_d_*. If this is to be determined from experimental data, then a separate procedure must be used and we outline such an approach in the Supplemental Material (section 7).

The variance of the measured square displacement

$$\text{Var}\left({\left|{\text{X}}_{t}\right|}^{2}\right)\equiv 𝔼\left({\left|{\text{X}}_{t}\right|}^{4}\right)-{\left[𝔼\left({\left|{\text{X}}_{t}\right|}^{2}\right)\right]}^{2}$$

can be shown (see Supplemental Material, section 1) to be

(4) $$\text{Var}\left({\left|{\text{X}}_{t}\right|}^{2}\right)\equiv 4\alpha^{2}t^{2}\left(2Dt+2\eta^{2}\right)+4{\left(2Dt+2\eta^{2}\right)}^{2}.$$

To our knowledge this result has not been explicitly stated before. In the absence of drift it is clear that Var(|***X**_t_*|^2^) = [*ρ*(*t*)]^2^ as the PDF for the measured squared displacement is an exponential distribution [13]. However, when drift is present then Var(|***X**_t_*|^2^) ≠ [*ρ*(*t*)]^2^ and hence the PDF for the squared displacements cannot be exponential. It is interesting to note that the variance of the squared displacement grows cubically in time when drift is present, whereas it only grows quadratically in the absence of drift. This observation has important implications when considering how to optimally infer the parameters of the model as time intervals which are too large may result in extremely noisy estimates of the MSD.

In terms of the experimental data, we will assume that there are *N_S_* observed trajectories, each comprising of particle coordinates using equal time interval between frames *t_n_* = *nT*/*N* = *n*Δ*t*, *n* = 0, …, *N*, covering the measurement time range [0, *T*]. The entire observed experimental data will therefore be denoted as

$${{\text{x}}_{n}}^{\left(j\right)}={\left({x_{n}}^{\left(j\right)},{y_{n}}^{\left(j\right)}\right)}^{T},\;1⩽n⩽N+1,\;1⩽j⩽N_{S}.$$

There are many possible ways to estimate the MSD [13] but the most widely used is the ensemble time-average overlapping MSD. This is constructed by first calculating *N_S_* time-averaged MSDs

(5) $$\begin{array}{l} {\rho_{n}}^{\left(j\right)}=\frac{1}{N+1-\;n}\sum_{i=1}^{N+1-n}|{\text{x}}_{i+n}^{\left(j\right)}-{{\text{x}}_{i}}^{\left(j\right)}{|}^{2},\\ \;n=1,\ldots ,N,\;j=1,\ldots ,N_{S}, \end{array}$$

then averaging over trajectories to obtain

(6) $$\rho_{n}=\frac{1}{N_{S}}\sum_{j=1}^{N_{S}}{\rho_{n}}^{\left(j\right)},\;n=1,\ldots ,N.$$

We will use a weighted least-squares fit to the *ρ_n_* values in the next section to estimate the parameters in the model and this requires the variance $\sigma_{n}^{2}$ of *ρ_n_*. In the Supplemental Material (section 2) we show that

(7) $$\sigma_{n}^{2}=\{\begin{array}{l} \begin{array}{ll} (\frac{n}{6K^{2}}\left(4n^{2}K+2K-n^{3}+n\right){\left(4D\Delta t\right)}^{2} & \\ \;+8\alpha^{2}D{\left(\Delta t\right)}^{3}\left[\frac{n^{3}}{3K^{2}}\left(3Kn+1-n^{2}\right)\right] & \\ \;+\frac{8\eta^{2}}{K^{2}}\{\left(K-n\right)\left[\eta^{2}-{\left(\alpha n\Delta t\right)}^{2}\right] & n⩽K\\ \;+K\left[{\left(\alpha n\Delta t\right)}^{2}+4Dn\Delta t+2\eta^{2}\right]\})/N_{S} & \end{array}\\ \begin{array}{ll} \{\frac{1}{6K}\left(6n^{2}K-4nK^{2}+4n+K^{3}-K\right){\left(4D\Delta t\right)}^{2} & \\ \;+8\alpha^{2}D{\left(\Delta t\right)}^{3}\left[\frac{n^{2}}{3K}\left(3nK-K^{2}+1\right)\right] & n>K\\ \;+\frac{8\eta^{2}}{K}\left[{\left(\alpha n\Delta t\right)}^{2}+4Dn\Delta t+2\eta^{2}\right]\}/N_{S}, & \end{array} \end{array}$$

where *K* = *N* + 1 − *n*. Note that in the absence of drift, the formulas above reduce to those appearing in Michalet [13].

**Fig. 1:**
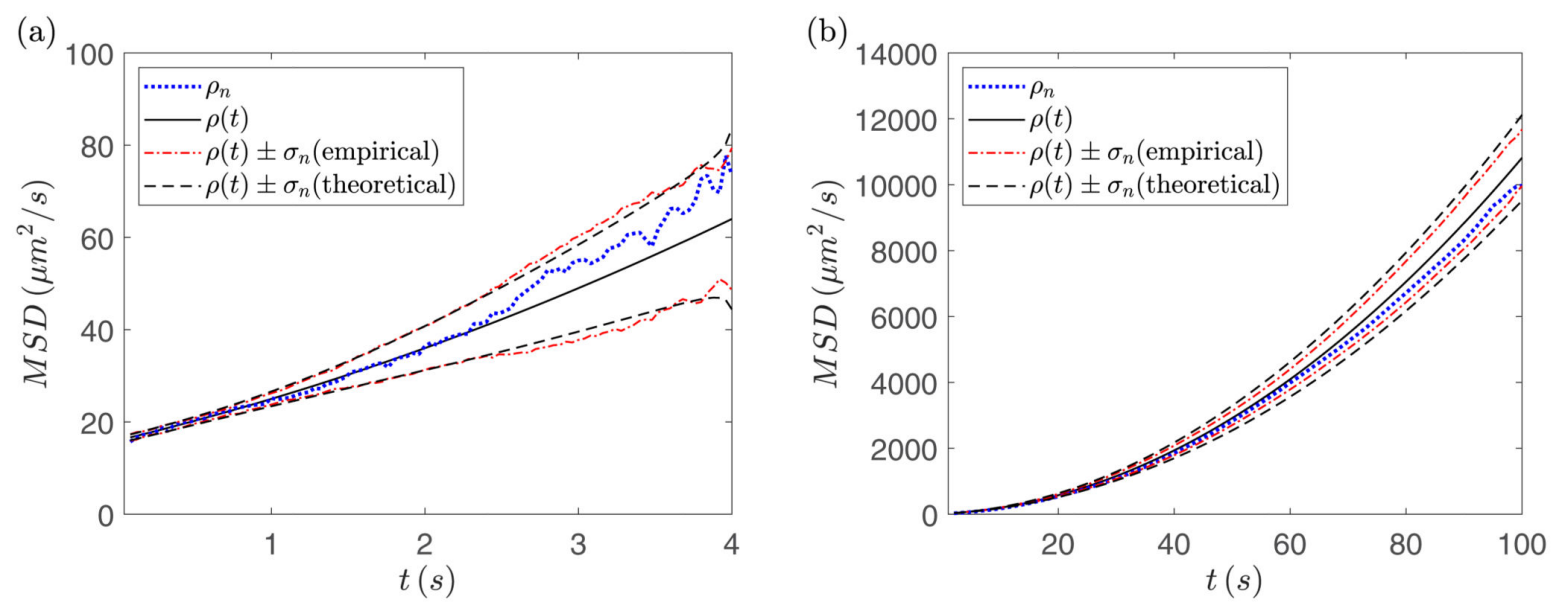
A plot of the theoretical MSD curve (3) (solid black line), the ensemble time-averaged estimate *ρ_n_* (6) (dotted blue line), along with *ρ*(*t*) ± *σ_n_*, where *σ_n_* is estimated empirically using 10 samples (dot-dashed red line) and *ρ*(*t*)±*σ_n_* where *σ_n_* is given by (7) (dashed black line), for a measurement time interval of *T* = 4 s (a) and *T* = 100 s (b). These experiments were for *D* = 2 *μ*m^2^/s, *α* = 1 *μ*m/s, *η* = 2 *μ*m, *N_S_* = 10, and *N* = 100.

To investigate the behavior of the MSD (3) as well as the quality of the ensemble time-averaged estimate (6), simulated data were obtained by solving numerically the drift-diffusion SDE (1) by the Euler-Maruyama method with *N_S_* = 10 trajectories and *N* = 100 time points. Figure 1 shows a plot of the theoretical MSD *ρ*(*t*) compared with the estimate *ρ_n_*. These experiments were for *D* = 2 *μ*m^2^/s, *α* = 1 *μ*m/s, *η* = 2 *μ*m. To estimate the uncertainty in *ρ_n_*, Fig. 1 also includes plots of *ρ_n_* ± *σ_n_*. Both the theoretical *σ_n_* given by (7) and an empirical estimate of *σ_n_*, obtained using 10 independent sample values of *ρ_n_*, are shown. The plot on the left shows simulations with a time interval of *T* = 4 s while the right plot shows simulations with the same parameter values but with a larger time interval *T* = 100 s. We can see that as time increases the size of the uncertainty in *ρ_n_* increases and for small times *ρ_n_* does not approximate *ρ*(*t*) well. This suggests a sufficiently large *T* is required in order to approximate the MSD accurately. We have also observed that choosing *T* too small lowers the accuracy of inferring the drift velocity, while taking the interval too large lowers the accuracy of inferring the diffusion coefficient. This is due to the quadratic form of the MSD, giving rise to two different timescales for the diffusive and drift processes.

### B Variance of the regression coefficients

Since *ρ*(*t*) = *a* + *bt* + *ct*^2^, where *a* = 4*η*^2^, *b* = 4*D*, and *c* = *α*^2^, the coefficients can be inferred by quadratic regression [23]. Let $\sigma_{n}^{2}$ be the variance of *ρ_n_* at the time point *t_n_* = *nT*/*N*, 1 ⩽ *n* ⩽ *N*, and $\sigma_{nm}^{2}=𝔼\left(\rho_{n}\rho_{m}\right)-𝔼\left(\rho_{n}\right)𝔼\left(\rho_{m}\right)$ be the covariance between *ρ_n_* and *ρ_m_*, where 1 ⩽ *n*, *m* ⩽ *N*.

For a quadratic polynomial of the form *μ*(*t*) = *a* + *bt* + *ct*^2^, the variance of the regression coefficients, calculated by fitting the first *p* MSD points, can be estimated by [13]

(8) $$\begin{array}{l} \sigma_{a}^{2}\approx \sum_{n=1}^{p}\sigma_{n}^{2}{\left(\frac{\partial a}{\partial \mu_{n}}\right)}^{2}+2\sum_{n=1}^{p}\sum_{m=1}^{n-1}\sigma_{nm}^{2}\left(\frac{\partial a}{\partial \mu_{n}}\right)\left(\frac{\partial a}{\partial \mu_{m}}\right),\\ \;3⩽p⩽N, \end{array}$$

(9) $$\begin{array}{l} \sigma_{b}^{2}\approx \sum_{n=1}^{p}\sigma_{n}^{2}{\left(\frac{\partial b}{\partial \mu_{n}}\right)}^{2}+2\sum_{n=1}^{p}\sum_{m=1}^{n-1}\sigma_{nm}^{2}\left(\frac{\partial b}{\partial \mu_{n}}\right)\left(\frac{\partial b}{\partial \mu_{m}}\right),\\ 3⩽p⩽N, \end{array}$$

(10) $$\begin{array}{l} \sigma_{c}^{2}\approx \sum_{n=1}^{p}\sigma_{n}^{2}{\left(\frac{\partial c}{\partial \mu_{n}}\right)}^{2}+2\sum_{n=1}^{p}\sum_{m=1}^{n-1}\sigma_{nm}^{2}\left(\frac{\partial c}{\partial \mu_{n}}\right)\left(\frac{\partial c}{\partial \mu_{m}}\right),\\ 3⩽p⩽N, \end{array}$$

where

(11) $$\frac{\partial a}{\partial \mu_{n}}=\frac{S_{2}S_{4}-S_{3}^{2}-S_{1}S_{4}t_{n}+S_{1}S_{3}t_{n}^{2}+S_{2}S_{3}t_{n}-S_{2}^{2}t_{n}^{2}}{\sigma_{n}^{2}\Delta },$$

(12) $$\frac{\partial b}{\partial \mu_{n}}=\frac{S_{0}S_{4}t_{n}-S_{0}S_{3}t_{n}^{2}-S_{1}S_{4}+S_{2}S_{3}+S_{1}S_{2}t_{n}^{2}-S_{2}^{2}t_{n}}{\sigma_{n}^{2}\Delta },$$

(13) $$\frac{\partial c}{\partial \mu_{n}}=\frac{S_{0}S_{2}t_{n}^{2}-S_{0}S_{3}t_{n}-S_{1}^{2}t_{n}^{2}+S_{1}S_{2}t_{n}+S_{1}S_{3}-S_{2}^{2}}{\sigma_{n}^{2}\Delta },$$

and

(14) $$\begin{array}{l} S_{k}=\sum_{n=1}^{p}\frac{{\left(t_{n}\right)}^{k}}{\sigma_{n}^{2}},\;k=0,\ldots ,4,\;3⩽p⩽N,\\ \Delta \;=\left|\begin{array}{lll} S_{0} & S_{1} & S_{2}\\ S_{1} & S_{2} & S_{3}\\ S_{2} & S_{3} & S_{4} \end{array}\right|\cdot \end{array}$$

Note that the lower limit for *p* reflects the minimum number of points needed to fit a quadratic polynomial, while the upper limit corresponds to fitting using all the MSD points. We show in the Supplemental Material (section 3) that the covariance of the MSD is

(15) $$\sigma_{nm}^{2}=\{\begin{array}{l} \begin{array}{ll} (\frac{16nD^{2}{\left(\Delta t\right)}^{2}}{6KP}\left\{-n^{3}-2Pn^{2}+\left[1-6m^{2}+6\left(N+1\right)m\right]n+2P\right\} & \\ \;+\frac{8\alpha^{2}{\left(\Delta t\right)}^{3}mn^{2}D}{3KP}\left\{-n^{2}+3\left[-m^{2}+\left(N+1\right)m+1/3\right]\right\}+\frac{32\eta^{2}nD\Delta t}{K} & \\ \;+\frac{8\eta^{4}\left(-n+2P\right)}{KP}+\frac{8\alpha^{2}{\left(\Delta t\right)}^{2}mn^{2}\eta^{2}}{KP})/N_{S} & m+n⩽N \end{array}\\ \begin{array}{ll} (\frac{8D^{2}{\left(\Delta t\right)}^{2}}{3K}\{-m^{3}+\left(3+3N-4n\right)m^{2}+\left[8\left(N+1\right)n-2-3N^{2}-6N\right]m & \\ \;-6n^{3}+6\left(N+1\right)n^{2}-\left(4N^{2}+8N\right)n+N\left(N+2\right)\left(N+1\right)\} & \\ \;+\frac{8\alpha^{2}D{\left(\Delta t\right)}^{3}mn}{3K}\left[m^{2}-2\left(N+1\right)m+3n^{2}-3\left(N+1\right)nN^{2}+2N\right] & m+n>N\\ \;+\frac{8\eta^{2}}{K}\left[\alpha^{2}{\left(\Delta t\right)}^{2}mn+4Dn\Delta t+\eta^{2}\right])/NS, & \end{array} \end{array}$$

where *K* = *N* + 1 − *n* and *P* = *N* + 1 − *m*. Again, in the absence of drift, the covariance (15) is exactly as stated in Michalet [13].

In this paper we are interested in the optimal estimation of the diffusion coefficient *D* and the drift magnitude *α*. Since these are related to the regression coefficients *b* and *c*, we look to minimize *σ_b_/b* + *σ_c_/c*, the relative errors in *b* and *c*. This can be done in two ways depending on the experimental protocol.

## III Results using the Optimal number of Fitting Points

### A Determination of the optimal number of fitting points

**Fig. 2:**
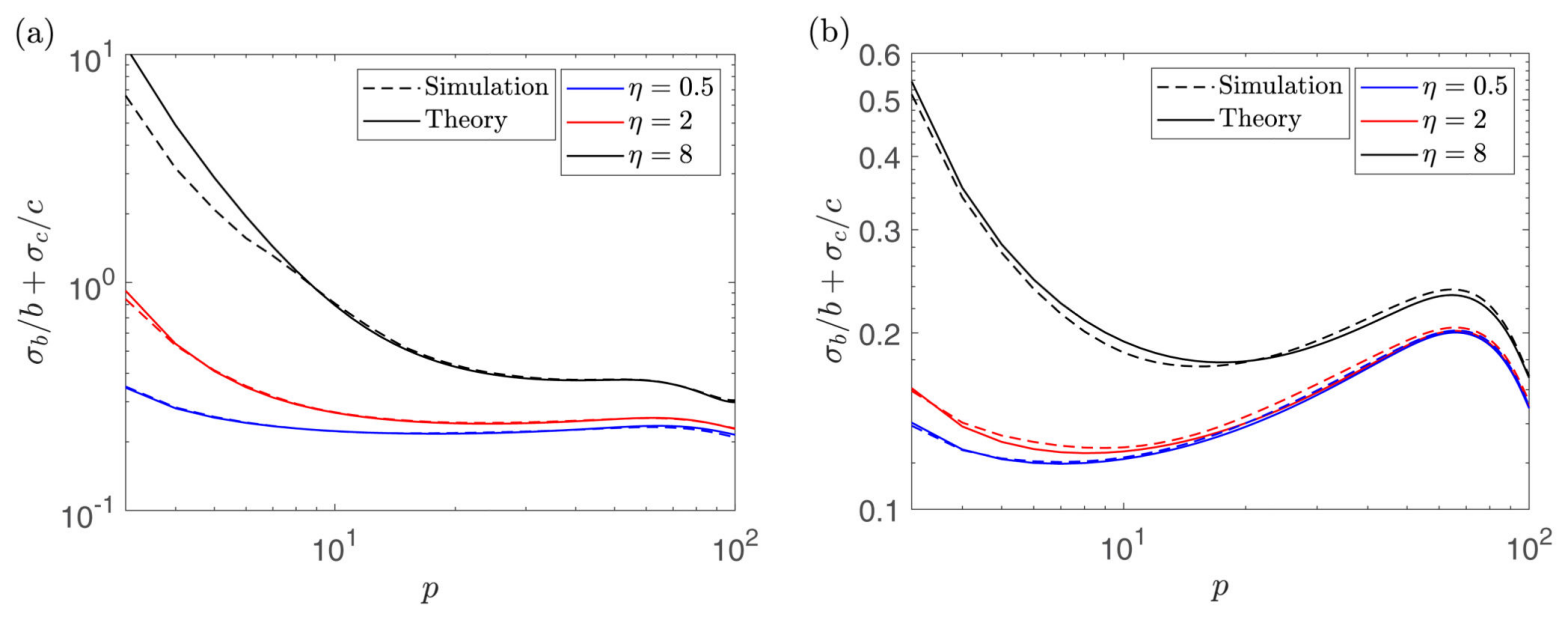
A plot of the theoretical value of *σ_b_/b* + *σ_c_/c* (solid lines) and its empirically estimated value using 1000 samples (dashed lines) when fit with the first *p* MSD points for *η* = 0.5 *μ*m, 2 *μ*m, and 8 *μ*m (from bottom to top, respectively, in both plots) for Δ*t* = 1 s giving *T* = 100 s (a) and Δ*t* = 10 s giving *T* = 1000 s (b). These experiments were for *D* = 2 *μ*m^2^/s, *α* = 1 *μ*m/s, *N_S_* = 10, and *N* = 100. The optimal number of fitting points are 100 for all values of *η* in (a) and 7, 8, and 100 for *η* = 0.5 *μ*m, 2 *μ*m, and 8 *μ*m, respectively, in (b).

If experiments cannot be repeated, then the optimal estimates of the model parameters may be obtained by fitting a subset of the MSD points. For this, we assume that the MSD is calculated using all *N* time points [as in Eqs. (5) and (6)] and then fit using a subset of these points (see Sec. IIB). In the Supplemental Material (section 4.1) we look at optimizing the number of fitting points for different choices of *N_S_* and *N*, as well as results for inferring the diffusion coefficient, drift magnitude, and standard deviation of the static error. To investigate optimizing the number of fitting points, we look at the theoretical value of the uncertainty *σ_b_/b* + *σ_c_/c* using (8)–(14) for different values of *p* and compare this with an empirical estimate calculated from simulations. For the estimated uncertainty, we calculate the MSD data points then use WLS regression to obtain estimates for *b* and *c* by fitting with the first *p* points, where 3 ⩽ *p* ⩽ *N*. This was repeated 1000 times to empirically estimate the values of *σ_b_* and *σ_c_*. Figure 2 shows the theoretical and simulated value of *σ_b_/b* + *σ_c_/c* as a function of the number of fitting points *p* for two different Δ*t* values for *η* = 0.5 *μ*m, 2 *μ*m, and 8 *μ*m. These experiments were for *D* = 2 *μ*m^2^/s, *α* = 1 *μ*m/s, *η* = 2 *μ*m, *N_S_* = 10, and *N* = 100, with Δ*t* = 1 *s* giving *T* = 100 s for the left plot, while Δ*t* = 10 s giving *T* = 1000 s for the right plot. We denote the optimal number of fitting points which minimizes *σ_b_/b* + *σ_c_/c* by *p*_opt_. First notice that we have good agreement between the simulations and the theoretical expressions. Although it is difficult to see, from the left plot when Δ*t* is small, the optimal estimation of the parameters is obtained using all 100 MSD points in the fitting for all values of *η* tested. On the other hand, if Δ*t* is taken to be larger, then there may be an optimal number of fitting points which is less than *N*. In the right plot, for *η* = 0.5 *μ*m, 2 *μ*m, and 8 *μ*m, we have that the optimal number of fitting points for each case are *p*_opt_ = 7, 8, and 100, respectively. The dependence of the optimal number of fitting points on Δ*t* is due to the two different timescales associated with drift and diffusion. Notice that the value of *p*_opt_ depends on the model parameters *D*, *α*, and *η*, as well as the size of the time interval between frames Δ*t* and the total number of time points *N*. In the Supplemental Material (section 4.2) we provide a MATLAB routine which determines *p*_opt_ (*D*, *α*, *η*, Δ*t*, *N*) given these input parameters.

**Algorithm 1 Iterative algorithm to find *p*_opt_ and estimates of *D*, *α*, and *η***

      **Input**: MSD data found at *N* fixed time points with time step Δ*t* = *T*/*N*, and convergence parameter *τ*.

      **Output**: Estimates of optimal number of fitting points *p*_opt_ and parameters *D*, *α* and *η*.

  1: Set the number of fitting points *p*_0_ = *N* and set *i* = 0.

  2: **if** *i* = 0 **then**

  3:       $\sigma_{n}^{2\left(i\right)}=1,\;1⩽n⩽p_{i},$

  4: **else**

  5:      $\sigma_{n}^{2\left(i\right)}=\sigma_{n}^{2}\left(D_{i},\alpha_{i},\eta_{i},\Delta t\right)$ using (7), 1 ⩽ *n* ⩽ *p_i_*.

  6: **end if**

  7: Use WLS regression with weights $1/\sigma_{n}^{2\left(i\right)}$ on the first *p_i_* points of the MSD to get the parameter estimates *D_i_*, *α_i_* and *η_i_*.

  8: Update *p*_*i*+1_ = *p*_opt_ (*D_i_*, *α_i_*, *η_i_*, Δ*t*, *N*).

  9: **if** (*p*_*i*+1_ − *p_i_*)/*p*_*i*+1_ < *τ* **then**

10:     end algorithm,

11: **else**

12:     Set *i* = *i* + 1 and go back to Step 2.

13: **end if**

### B Iterative algorithm to calculate *p*_opt_

The difficulty with using *p*_opt_ (*D*, *α*, *η*, Δ*t*, *N*) to infer the model parameters is we require the values of *D*, *α*, and *η* themselves in order to calculate it. We therefore consider the following iterative technique for determining *p*_opt_. The iterative algorithm initially estimates *D*, *α*, and *η* by fitting all *N* MSD points. The weighting used in the fitting is initially taken to be uniform, and then, for all future iterations, we estimate the variance of the MSD by substituting the current parameter estimates into (7). The algorithm then adapts the number of fitting points according to Algorithm 1.

The tolerance *τ* determines the stopping criterion depending on the relative differences between two successive *p_i_* values.

**Fig. 3:**
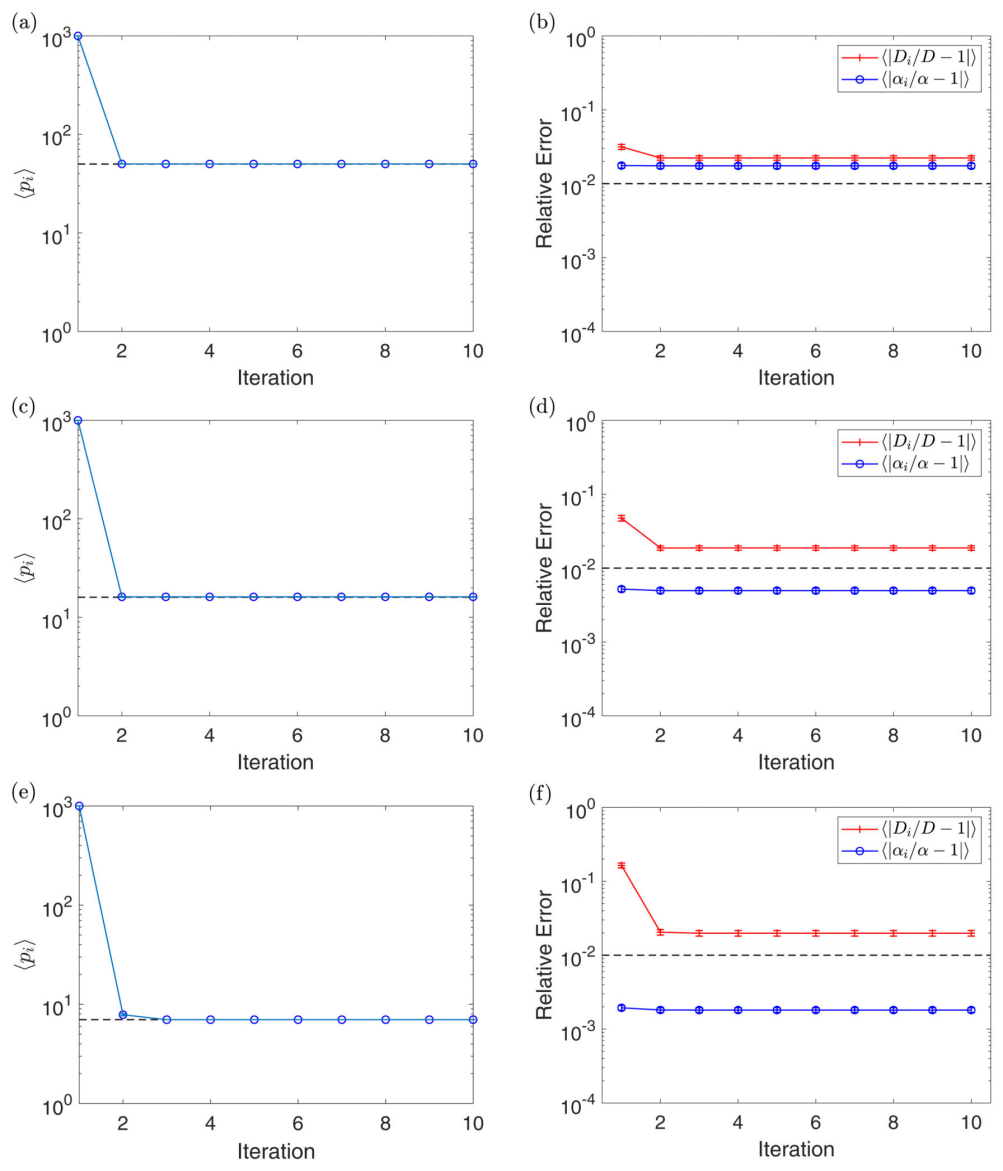
A plot of the value of 〈*p_i_*〉 for each iteration with standard error bars [(a), (c), and (e)], along with a plot of the value of 〈|*D_i_/D* −1|〉 (red crosses) and 〈|*α_i_/α* − 1|〉 (blue circles) for each iteration with standard error bars [(b), (d), and (f)] for Δ*t* = 1 s [(a) and (b)], Δ*t* = 10 s [(c) and (d)], and Δ*t* = 100 s [(e) and (f)]. These experiments were for *D* = 2 *μ*m^2^/s, *α* = 1 *μ*m/s, *η* = 2 *μ*m, *N_S_* = 10, and *N* = 1000. The dashed line in the plots of 〈*p_i_*〉 correspond to *p*_opt_ = 50 (a), *p*_opt_ = 16 (c), and *p*_opt_ = 7 (e), while the dashed line in the plots of 〈|*D_i_/D* − 1|〉 and 〈|*α_i_/α* − 1|〉 correspond with the value 10^−2^, indicating a 1% error.

We tested the iterative algorithm for the parameter values *D* = 2 *μ*m^2^/s, *α* = 1 *μ*m/s, and *η* = 2 *μ*m for three different time steps, Δ*t* = 1 s, Δ*t* = 10 s, and Δ*t* = 100 s. Each simulation run uses *N* = 1000 time points and *N_S_* = 10 trajectories to create the MSD data and a quadratic fit. Since simulations are likely to end after a different number of iterations, Steps 9–11 of Algorithm 1 will be ignored and instead all simulations are stopped after 10 iterations. These simulations were then repeated 100 times. By denoting the mean value of a quantity by the angular brackets 〈·〉 we indicate the performance of the algorithm by plotting 〈*p_i_*〉, 〈|*D_i_*/*D* − 1|〉, and 〈|*α_i_/α* − 1|〉 in Fig. 3. The first thing to notice is that the algorithm converges to *p*_opt_ in a couple of iterations for the cases considered, with most being after just one iteration. We do not see much improvement in 〈|*α_i_/α* − 1|〉 when fit with the optimal number of fitting points, compared with all the MSD points, for any value of Δ*t*. However, we do see a decrease in its value as we increase Δ*t*. This is due to the value of the measurement time interval *T* increasing as we increase Δ*t*. This increase in *T* moves us into the drift timescale where the inference of *α* is better. The value of 〈|*D_i_*/*D* − 1|) decreases after one iteration in all cases, with a larger decrease for larger values of Δ*t*. The final value of 〈|*D_i_*/*D* − 1|) decreases when Δ*t* = 1 s is increased to Δ*t* = 10 s but then increases for Δ*t* = 100 s. Here a small value of Δ*t*, corresponding with a small value of *T*, is likely to give data which is static error dominated. When we increase Δ*t* we leave the noisy domain and so the inference of *D* is improved. However, when we increase *T* too much, we leave the diffusive timescale and so the inference of *D* begins to deteriorate. This example shows that the choice of Δ*t* is important for the optimal inference of both the parameters. Additional experiments were run for different values of *D* and *α*, which can be found in the Supplemental Material (section 4.3).

### C Single-particle parameter estimation using *p*_opt_

While the analysis and results presented so far assume the availability of data for an ensemble of particles, in some situations only single-particle data are available. We now consider how the results we have perform in the single-particle case. An important point to note is that the optimal number of fitting points for both the single-particle case and ensemble of particles case are identical. This is because when calculating the variance and covariance of the MSD in the ensemble particle case, we simply take the single-particle variance and covariance and divide by *N_S_*, as stated in the Supplemental Material (sections 2 and 3). Hence, when calculating the variance of the regression coefficients in (8)–(10), for the ensemble case, we can take out a factor of 1/*N_S_* from $\sigma_{n}^{2}$ and $\sigma_{nm}^{2}.$ Therefore, the value of *σ_b_*/*b* + *σ_c_*/*c* in the ensemble case will be a factor of $\sqrt{N_{S}}$ smaller than the single-particle case but the shape of the curve will be the same in both cases.

When using Algorithm 1 with an ensemble of particles, Steps 2–6 could be ignored and the variance of the MSD can be estimated empirically from the data. This obviously cannot be done for the single-particle case. This stresses the importance of having the theoretical expression for the variance of the MSD (7) as WLS regression can be still be done using single-particle data.

**Fig. 4:**
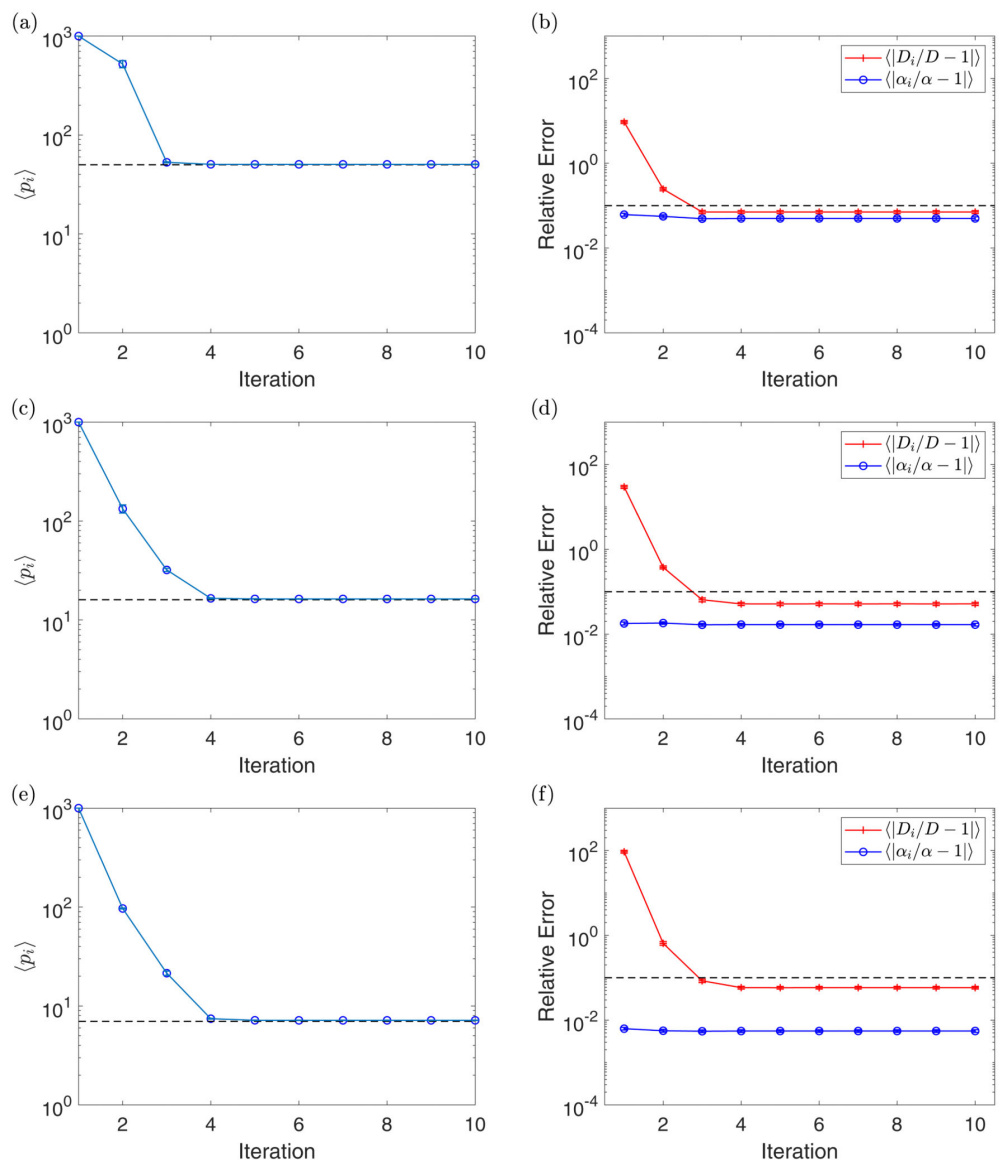
A plot of the value of 〈*p_i_*〉 for each iteration with standard error bars [(a), (c), and (e)], along with a plot of the value of 〈|*D_i_/D* − 1|〉 (red crosses) and 〈|*α_i_/α* − 1|〉 (blue circles) for each iteration with standard error bars [(b), (d), and (f)] for Δ*t* = 1 s [(a) and (b)], Δ*t* = 10 s [(c) and (d)], and Δ*t* = 100 s [(e) and (f)]. These experiments were for *D* = 2 *μ*m^2^/s, *α* = 1 *μ*m/s, *η* = 2 *μ*m, *N_S_* = 1, and *N* = 1000. The dashed line in the plots of 〈*p_i_*〉 correspond to *p*_opt_ = 50 (a), *p*_opt_ = 16 (c), and *p*_opt_ = 7 (e), while the dashed line in the plots of 〈|*D_i_/D* − 1|〉 and 〈|*α_i_/α* − 1|〉 correspond with the value 10^−1^, indicating a 10% error.

**Fig. 5:**
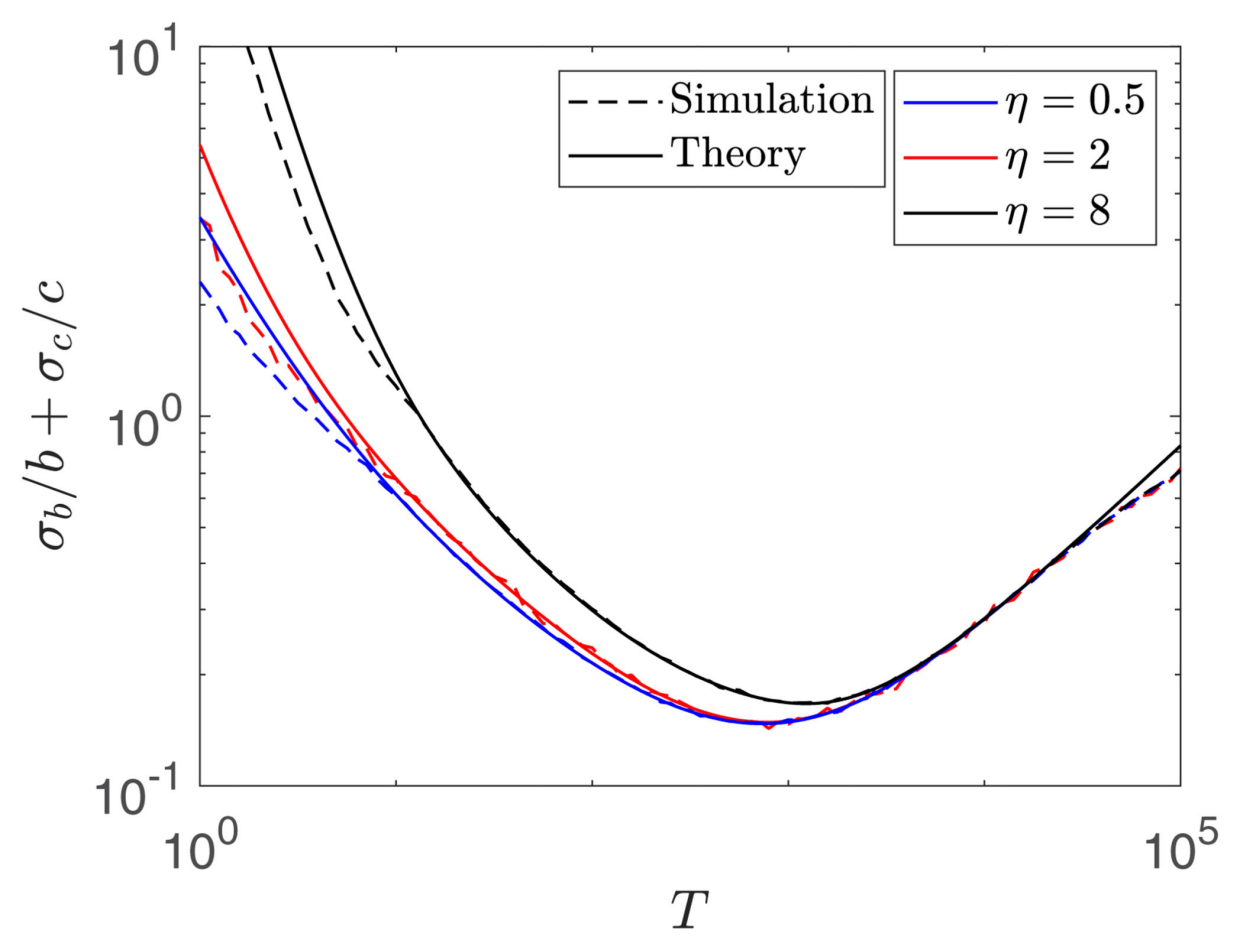
A plot of the theoretical value of *σ_b_/b* + *σ_c_/c* (solid lines) and its empirically estimated value using 1000 samples (dashed lines) against many different values of *T* for *η* = 0.5 *μ*m, 2 *μ*m, and 8 *μ*m (bottom to top, respectively). These experiments were for *D* = 2 *μ*m^2^/s, *α* = 1 *μ*m/s, *N_S_* = 10 and *N* = 100. For *η* = 0.5 *μ*m, 2 *μ*m, and 8 *μ*m, the optimal measurement time intervals are *T*_opt_ ≈ 735 s, 780 s, and 1216 s, respectively.

Figure 4 shows the results of the iterative algorithm for the same parameter values as in Fig. 3 but for *N_S_* = 1. Since we only have a single particle, we expect the relative errors to be higher. Therefore, in each right plot, the dashed line will now correspond to a 10% error. Notice that the value of 〈*p_i_*〉 takes a couple more iterations to converge but still does so in a small number of iterations. We often see the relative errors converge before 〈*p_i_*〉, which is a result of the shallow minimum around *p*_opt_ in the right plot of Fig. 2. We have also observed similar behavior for repeatable experiments; for example, Figs. 5 and 7. We see the same trend for 〈|*α_i_*/*α* − 1|〉 as before, namely that fitting with the optimal number of fitting points does not improve its value much, but using a large value of Δ*t* does. However, we see that the value of 〈|*D_i_*/*D* − 1|〉 is significantly improved; for example, looking at the case where Δ*t* = 100 s, we start with around a 10 000% error and end below a 10% error. This is a considerable improvement compared with the ensemble case seen in Fig. 3. We provide further examples of single-particle experiments for different values of *D* and *α* in the Supplemental Material (section 4.4).

## IV Results using the Optimal Measurement Interval

### A Determination of the optimal measurement time interval

If experiments are able to be repeated then the optimization can be done with respect to the measurement time interval *T* rather than the number of MSD fitting points. This has the advantage that the optimal measurement time interval could help inform future experiments. For this method we assume that the MSD is calculated from all *N* time points and that all *ρ_n_* data points are used in the fitting process. Note that since all the MSD points are used in the fitting, a new value of *T* will correspond with a new value of Δ*t*. As stated before we concentrate on the optimal inference of the diffusion coefficient and drift magnitude. In the Supplemental Material (section 5.1) we again present similar results for different choices of *N_S_* and *N*, as well as results for inferring the diffusion coefficient, drift magnitude and the standard deviation of the static error.

Here, the theoretical uncertainty *σ_b_/b* + *σ_c_/c* is calculated over many different values of *T* using (8)–(14) with *p* = *N* so that all the MSD points are used in the fitting, and is compared with simulations. The simulated result was found by calculating the MSD and using WLS regression to obtain estimates of *b* and *c*. This was repeated 1000 times to obtain estimates of *σ_b_* and *σ_c_*. Figure 5 shows the comparison between the theoretical and simulated value of *σ_b_/b* + *σ_c_/c* over many different values of *T* for *η* = 0.5 *μ*m, 2 *μ*m, and 8 *μ*m. These experiment were for *D* = 2 *μ*m^2^/s, *α* = 1 *μ*m/s, *N_S_* = 10, and *N* = 100. We denote the value of *T* which minimizes the uncertainty *σ_b_/b* + *σ_c_/c* by *T*_opt_. We can see that we have good agreement between the theory and simulated uncertainties, more so closer to *T*_opt_. We also see that for all the cases tested, there exists an optimal measurement time interval. For *η* = 0.5 *μ*m, 2 *μ*m, and 8 *μ*m these optimal measurement time intervals are *T*_opt_ ≈ 735 s, 780 s, and 1216 s. In the Supplemental Material (section 5.2) we provide a matlab routine which determines *T*_opt_ (*D*, *α*, *η*, *N*) given the input parameters.

### B Iterative algorithm to calculate *T*_opt_

As before, the function to calculate *T*_opt_ depends on the model parameters and so another iterative algorithm was created. Note that each new iteration corresponds with repeating the experiment with a new measurement time interval *T_i_*. To begin the iteration we need to provide an initial guess for *T*_opt_, which we denote by *T*_0_, with time interval between frames Δ*t*_0_ = *T*_0_/*N*. The algorithm then adapts the time according to Algorithm 2.

The role of the under-relaxation parameter *ω_i_* is to improve the robustness of the algorithm by reducing oscillations; this is effectively a low-pass filter for the time series of adjustments. For example, if the initial guess *T*_0_ is far from the optimal value *T*_opt_, then the values *T_i_* will quickly be adapted toward the optimal time. Close to the optimal time the algorithm can display oscillations in the convergence behavior, i.e., (*T*_*i*+1_ − *T_i_*) × (*T_i_* − *T*_*i*−1_) < 0. When this occurs the relaxation parameter *ω_i_* is decreased to smooth out the difference between iterates. The tolerance *τ* determines when to stop the algorithm depending on the relative differences between two successive time points. The rate at which the value of *ω_i_* is decreased in Step 10 is determined by the adjustment parameter *ψ* where 0 < *ψ* ⩽ 1. In the experiments that follow, the value of *ψ* = 0.8 has been used but additional values of *ψ* were tested in the Supplemental Material (section 5.3).

**Algorithm 2 Iterative algorithm to find *T*_opt_ and estimates of *D*, *α*, and *η***

    **Input**: Initial estimate of measurement time interval *T*_0_ and measurement interval between frames Δ*t*_0_, number of time points *N*, adaptation parameter *ψ* and convergence parameter *τ*.

    **Output**: Estimates of optimal time *T*_opt_ and parameters *D*, *α* and *η*.

1: Guess an initial time *T*_0_ with corresponding Δ*t*_0_ and set the relaxation parameter *ω*_0_ = 1 and set *i* = 0.

2: **if** *i* = 0 **then**

3: $\sigma_{n}^{2\left(i\right)}=1,\;1⩽n⩽N,$

4: **else**

5:      $\sigma_{n}^{2\left(i\right)}=\sigma_{n}^{2}\left(D_{i},\alpha_{i},\eta_{i},\Delta t_{i}\right)$ using (7), 1 ⩽ *n* ⩽ *N*.

6: **end if**

7: Calculate the MSD at the *N* time points with interval Δ*t_i_* up to *T_i_* and use WLS on all the points with weights $1/\sigma_{n}^{2\left(i\right)}$ to get the parameter estimates *D_i_*, *α_i_* and *η_i_*.

8: Update *T*_*i*+1_ = (1 − *ω_i_*)*T_i_* + *ω_i_T*_opt_ (*D_i_*, *α_i_*, *η_i_*, *N*) and calculate Δ*t*_*i*+1_ = *T*_*i*+1_/*N*.

9: **if** *i* ⩾ 2 and (*T*_*i*+1_ − *T_i_*) × (*T_i_* − *T*_*i*−1_) < 0 **then**

10:      *ω*_*i*+1_ = *ψ* × *ω_i_*, 0 < *ψ* ⩽ 1

11: **else**

12:      *ω*_*i*+1_ = *ω_i_*

13: **end if**

14: **if** (*T*_*i*+1_ − *T_i_*)/*T*_*i*+1_ < *τ* **then**

15:      end algorithm

16: **else**

17:      Set *i* = *i* + 1 and go back to Step 2

18: **end if**

**Fig. 6:**
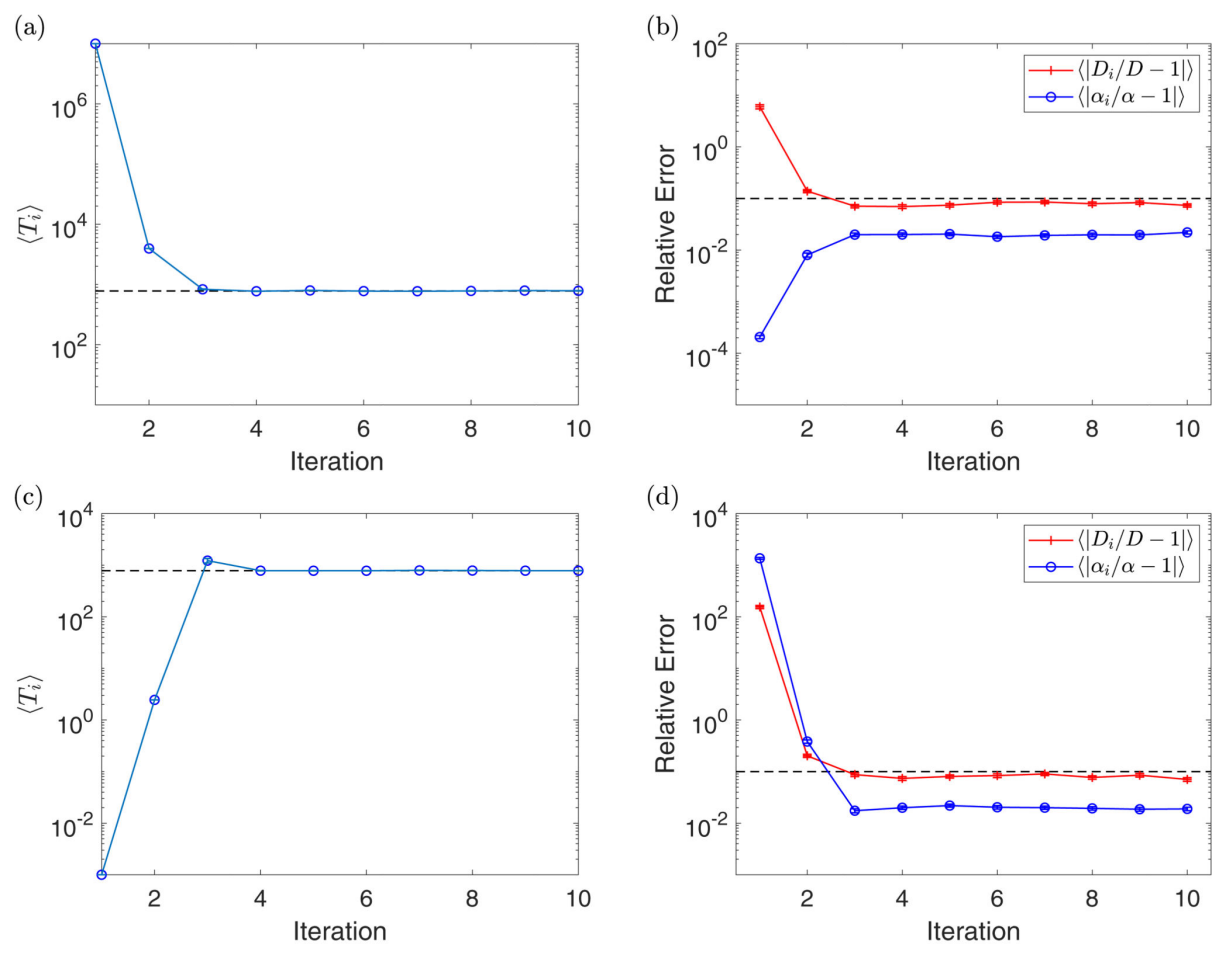
A plot of the value of 〈*T_i_*〉 for each iteration with standard error bars [(a) and (c)], along with a plot of the value of 〈|*D_i_/D* − 1|〉 (red crosses) and 〈|*α_i_/α* − 1|〉 (blue circles) for each iteration with standard error bars [(b) and (d)]. These experiments were for *D* = 2 *μ*m^2^/s, *α* = 1 *μ*m/s, *η* = 2 *μ*m, *N_S_* = 10, and *N* = 100, with a starting time of *T*_0_ = 10^7^ s [(a) and (b)] and *T*_0_ = 10^−3^ s [(c) and (d)]. The dashed line in the plots of 〈*T_i_* 〉 correspond to *T*_opt_ ≈ 780 s, while the dashed line in the plots of 〈|*D_i_/D* − 1|〉 and 〈|*α_i_/α* − 1|〉 correspond with the value 10^−1^, indicating a 10% error.

The iterative algorithm was tested for the two different initial measurement time intervals, *T*_0_ = 10^7^ s and *T*_0_ = 10^−3^ s. Both experiments were for *D* = 2 *μ*m^2^/s, *α* = 1 *μ*m/s, *η* = 2 *μ*m, *N_S_* = 10, and *N* = 100; for these parameters *T*_opt_ ≈ 780 s. Again, Steps 14–16 of Algorithm 2 will be ignored and instead all simulations are stopped after 10 iterations. These simulations were then repeated 100 times. The quantities 〈*T_i_*〉, 〈|*D_i_*/*D* − 1|〉 and 〈|*α_i_*/*α* − 1|〉 are shown in Fig. 6. Notice that the initial guess *T*_0_ = 10^7^ s significantly overestimates the true value of *T*_opt_, but that the value of 〈*T_i_*〉 converges rapidly to a value close to *T*_opt_. While the value of 〈|*α_i_*/*α* − 1|〉 becomes less accurate as we progress, the value of 〈|*D_i_*/*D* − 1|〉 quickly falls from around a 1000% error to under a 10% error in a small number of iterations. When using a much smaller initial time of *T*_0_ = 10^−3^ s, we see that 〈*T_i_*〉 still converges to *T*_opt_ in a small number of iterations. Initially the value of 〈|*D_i_*/*D* − 1|〉 is of the order of magnitude 10^2^ while 〈|*α_i_*/*α* − 1|〉 is of the order of magnitude 10^3^, corresponding to a 10 000% and 100 000% error, respectively. This highlights the fact that an incorrect choice of *T* can lead to very large inaccuracies in the value of inferred parameters. However, using the adaptive algorithm we see that as the 〈*T_i_*〉 values get closer to *T*_opt_, the errors both reduce to under 10%. This stresses the importance of using *T*_opt_ when inferring *D* and *α* using all the MSD points in the fitting. Additional experiments were run for different values of *D* and *α*, which can be found in the Supplemental Material (section 5.3).

### C Single-particle parameter estimation using *T*_opt_

**Fig. 7:**
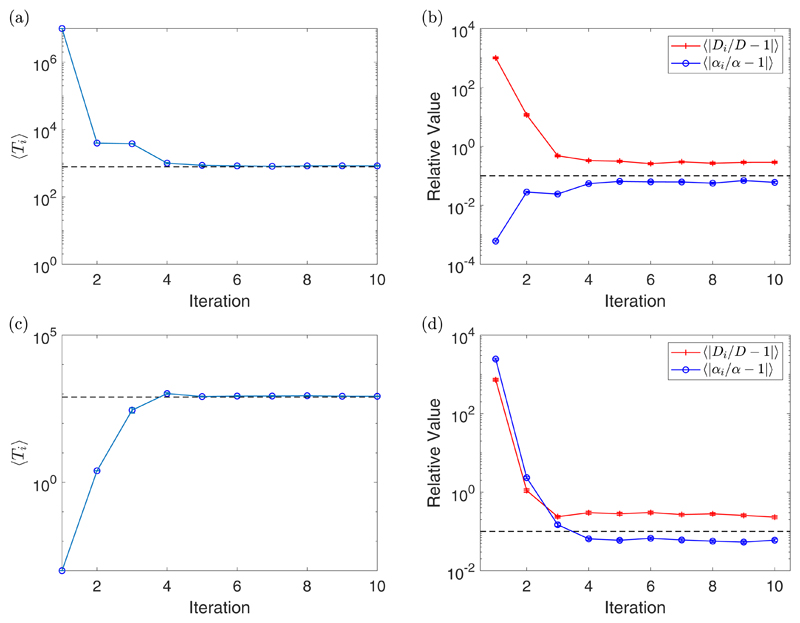
A plot of the value of 〈*T_i_*〉 for each iteration with standard error bars [(a) and (c)], along with a plot of the value of 〈|*D_i_/D* − 1|〉 (red crosses) and 〈|*α_i_/α* − 1|〉 (blue circles) for each iteration with standard error bars [(b) and (d)]. These experiments were for *D* = 2 *μ*m^2^/s, *α* = 1 *μ*m/s and *η* = 2 *μ*m, *N_S_* = 1 and *N* = 100, with a starting time of *T*_0_ = 10^7^ s [(a) and (b)] and *T*_0_ = 10^−3^ s [(c) and (d)]. The dashed line in the plots of 〈*T_i_*〉 correspond to *T*_opt_ ≈ 780 s, while the dashed line in the plots of 〈|*D_i_/D* − 1|〉 and 〈|*α_i_/α* − 1|〉 correspond with the value 10^−1^, indicating a 10% error.

The results for *T*_opt_ can also extend to the single-particle case for the same reasons as the *p*_opt_ method. The optimal measurement time interval will be the same for an ensemble of particles and the single-particle cases. Figure 7 compares the performances using the same initial measurement time intervals, *T*_0_ = 10^7^ s and *T*_0_ = 10^−3^ s, for the same parameter values as those in Fig. 6 but with *N_S_* = 1. The value of 〈*T_i_*〉 continues to converge in a small number of iterations and we observe that the results for 〈|*D_i_*/*D* − 1|〉 and 〈|*α*_*i*_/*α* − 1|〉 have similar dynamics to the ensemble case. These show the strength of the iterative algorithm as they give good results even in the single-particle case where we have less information. Further experiments for different values of *D* and *α* can be found in the Supplemental Material (section 5.4).

The practical feasibility of this procedure to change the measurement time interval depends on the chosen application domain. For instance, in environmental statistics, where the task is, e.g., to monitor the spread of pollutants and contaminants in ground water, it is common practice to repeatedly estimate the same physical quantities. This setting therefore naturally lends itself to the integration of the proposed iterative adjustment scheme. For other applications, like the study of collective cell movement with high-resolution microscopy, a change of the experimental protocol may be required to allow (and budget) for a series of experiments that enable iterative adjustments of the measurement time intervals.

## V Discussion

### A Fitting method

**Fig. 8:**
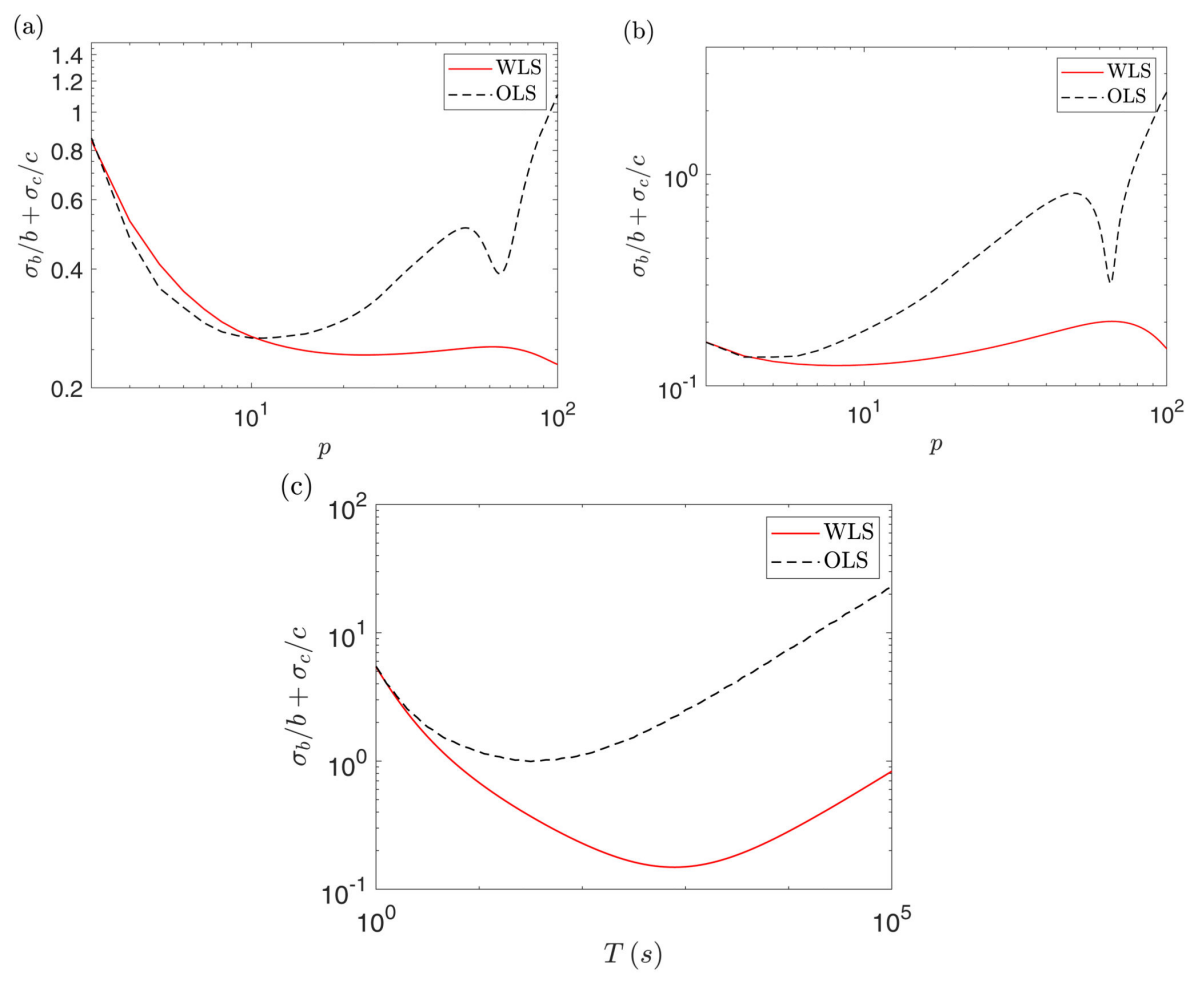
A plot of an empirically estimated value of *σ_b_/b* + *σ_c_/c* using WLS (solid red line) and OLS (dashed black line) as a function of the number of fitting points [(a) and (b)] or fit with all the MSD points over a number of measurement time intervals (c). These experiments were for *D* = 2 *μ*m^2^/s, *α* = 1 *μ*m/s, *η* = 2 *μ*m, *N_S_* = 10, and *N* = 100. For the plots using the number of fitting points we have that Δ*t* = 1 *s* giving *T* = 100 s (a) and Δ*t* = 10 s giving *T* = 1000 s (b).

Throughout the paper we assume that WLS regression is used to infer the parameters of the model. Michalet [13] showed that, in the absence of drift, the uncertainty in the parameters when using WLS and ordinary least squares (OLS) were similar as long as the optimal number of fitting points were used. When drift is included we find that WLS gives better results, both for using the optimal number of fitting points and the optimal measurement time interval, as shown in Fig. 8. We can see that in all cases using WLS gives better results than using OLS. When fitting with a subset of the MSD points, corresponding to the top plots, we do not have a big difference in the minimal uncertainty between WLS and OLS, but when optimizing the measurement time interval, corresponding to the bottom plot, we see a significant difference, with WLS being almost an order of magnitude better.

### B Initial parameter estimates

Our analysis has shown that the optimal number of fitting points or the optimal measurement time depend on the quantities of interest themselves—the diffusion coefficient *D* and the drift magnitude *α* − via

(16) $$p_{\text{opt}}=p_{\text{opt}}\left(D,\alpha ,\eta ,\Delta t,N\right)\;\text{and}\;T_{\text{opt}}=T_{\text{opt}}\left(D,\alpha ,\eta ,N\right).$$

If we have reliable initial guesses for these parameters, then they can simply be inserted into (16) to estimate *p*_opt_ or *T*_opt_; however, such specific prior knowledge is rarely available. What we usually do have, though, is prior knowledge in terms of interval bounds or, more generally, prior probabilities. The simplest case is a uniform distribution of a prior credible interval, but more general forms of distributions may be derived from first principles; let us denote them by *p*(*D*), *p*(*α*), and *p*(*η*). From this, we can derive the prior expectation of *T*_opt_:

$$T_{\text{opt}}^{0}=\int T_{\text{opt}}\left(D,\alpha ,\eta ,N\right)p\left(D\right)p\left(\alpha \right)p\left(\eta \right)\;dDd\alpha d\eta ,$$

which in practice can be estimated with a Monte Carlo sum:

$$T_{\text{opt}}^{0}=\frac{1}{M}\sum_{i=1}^{M}T_{\text{opt}}\left(D,\alpha ,\eta ,N\right),$$

where (*α_i_*, *D_i_*, *η_i_*) are independent draws from *p*(*α*)*p*(*D*)*p*(*η*) with sample size *M*. This provides a good initial guess for the unknown optimal time *T*_opt_. A similar procedure could be used to generate an initial estimate for *p*_opt_.

### C Practical considerations in applications

An implicit assumption on which the proposed methodology is based is that of complete observation. This may not be valid in a real experiment, with missing values caused, e.g., by fluorophore bleaching. If the proportion of missing values is small, and values are missing at random, then there are established statistical procedures based, e.g., on the expectation-maximization algorithm [24], which replace the missing values of the complete-observation model by their conditional means, given the current parameter values, and then optimize both in an iterative procedure. While this iteration suffers from an increase in the computational complexity, the changes to the mathematical procedure and estimation equations are minimal. The challenge of dealing with missing values is, in general, more complex if data are missing systematically, e.g., as a consequence of particles leaving the field of view of the camera. However, in this situation Algorithm 1 can be used with single-particle data to determine the drift and diffusion parameters for each particle using the optimal number of fitting points assuming the frame rate is fixed. This procedure will produce a distribution for each parameter, which can be analyzed to determine if the model assumption of equal diffusion and drift parameters for each particle is valid. If it is, then the empirical mean and variance can be used for parameter estimation and uncertainty quantification. Using the optimal number of fitting points for each particle will reduce the spread in the parameter distributions which would result if a nonoptimal number of fitting points were used as originally discussed by Saxton [12] and Michalet [13].

A further assumption has been that the advection-diffusion model of Eq. (1) provides an accurate mathematical description of the true process under investigation. This may not be the case, e.g., due to inhomogeneities in the medium, leading to a more complex spatial distribution of the advection and diffusion parameters. In addition, there has recently been much interest in modeling animal movement (e.g., Hooten *et al.* [25]) and cell movement (e.g., Jones *et al.* [2]) with advection-diffusion type processes. However, in these cases we are not dealing with genuine physical processes but with more complex biological processes that merely exhibit similar characteristics. So an important question is that of model critique, i.e., to establish whether the assumed mathematical process provides an adequate description of the observed data. To this end, one can choose from a series of statistical techniques, ranging from computationally cheap asymptotic methods, like chi-square and G tests (e.g., McDonald [26]), to computationally more expensive nonasymptotic procedures, like the parametric bootstrap [27,28]. However, what all these methods have in common is the assumption of a reliable procedure for accurate parameter estimation in the assumed model, as otherwise a correct or adequate model may be rejected erroneously. Hence any form of model critique will greatly benefit from the improved parameter estimation procedure proposed in the present paper.

There are many scenarios where we want to discriminate between alternative models based on the observed data. For instance, we may want to establish whether the system of interest is subject to advection as opposed to be driven by diffusion only. In other scenarios, we may want to know if there are significant other driving forces in the system in addition to advection and diffusion. Since these models are nested, one can fit the MSD data and then use an F-test to test the null hypothesis that the data have arisen from the simpler model. Again, the procedure is based on the assumption that the model parameters have been estimated accurately. As we have shown, this depends strongly on how the MSD data are fitted, and the proposed procedure for variance reduction makes a important contribution here.

## VI Conclusions

When particles are assumed to undergo Brownian motion with drift, and the measured position of the particles is subject to static localization error, the accurate inference of model parameters is dependent on either the number of MSD points used in the WLS fitting or the measurement time interval, depending on the experimental protocol. In both cases, when *T* is too small we get inaccurate estimates for the drift magnitude, as well as the data being dominated by the static error, while larger values of *T* result in inaccurate inference of the diffusion coefficient. For experiments which cannot be repeated, an optimal number of fitting points *p*_opt_ was found which optimized the inference of the model parameters. Similarly, for repeatable experiments, an optimal measurement time interval *T*_opt_ was found. Both *p*_opt_ and *T*_opt_ depended on the parameters themselves and so an iterative algorithm was created for both procedures which gives optimally accurate estimates of the parameters. This depended on the calculation of an analytical form for the variance and covariance of the time-average overlapping MSD, particularly the variance as this could be used to perform WLS in the single-particle case.

## Supplementary Material

Supplementary Material
