# Supplementary material for: Optimal estimation of drift and diffusion coefficients in the presence of static localization error
Source: Phys Rev E. Author manuscript; Available in PMC 2019 Oct 4. (PMC6778050; doi:10.1103/PhysRevE.100.022134)
Supplement: Supplementary Material [file EMS84213-supplement-Supplementary_Material.pdf]

Supplementary Material for "Optimal estimation of drift and diffusion coefficients in the presence of observation noise"

J. Devlin, D. Husmeier and J.A. Mackenzie

1 Mean and variance of the squared displacement

Since the particles are assumed to follow the drift-diffusion stochastic differential equation, the probability density function (PDF) for the observed trajectory $p(\boldsymbol{x}, t)$ being at location $\boldsymbol{x} = (x(t), y(t))$ at time t is given by

$$p(\boldsymbol{x}, t) = \frac{1}{2\pi(2Dt + 2\eta^2)} \exp\left(\frac{-|\boldsymbol{x} - \boldsymbol{\alpha}t|^2}{2(2Dt + 2\eta^2)}\right),$$

where D is the diffusion coefficient, $\boldsymbol{\alpha} = \alpha(\cos(\theta_d), \sin(\theta_d))$ is the drift velocity with drift direction θ_d, and η^2 is the variance of the observational noise.

The mean-square displacement (MSD) is calculated as

$$\rho(t) \equiv \mathbb{E}(|\boldsymbol{X}_t|^2) = \int_{\mathbb{R}^2} |\boldsymbol{x}|^2 \, p(\boldsymbol{x}, t) \, \mathrm{d}\boldsymbol{x}.$$

Substituting in the expression for $p(\boldsymbol{x}, t)$ gives

$$\rho(t) = \frac{1}{2\pi(2Dt + 2\eta^2)} \int_{-\infty}^{\infty} \int_{-\infty}^{\infty} (x^2 + y^2) \exp\left(\frac{-|\boldsymbol{x} - \boldsymbol{\alpha}t|^2}{2(2Dt + 2\eta^2)}\right) \mathrm{d}x\mathrm{d}y. \tag{1}$$

With $(\alpha_1, \alpha_2) = (\alpha\cos(\theta_d), \alpha\sin(\theta_d))$, we use the change of variables $x = \alpha_1 t + r\cos\theta$ and $y = \alpha_2 t + r\sin\theta$ for $0 \le r < \infty$ and $0 \le \theta \le 2\pi$, then

$$\mathrm{d}x\mathrm{d}y = \begin{vmatrix} x_r & x_\theta \\ y_r & y_\theta \end{vmatrix} \mathrm{d}r\mathrm{d}\theta = \begin{vmatrix} \cos\theta & -r\sin\theta \\ \sin\theta & r\cos\theta \end{vmatrix} \mathrm{d}r\mathrm{d}\theta = r\,\mathrm{d}r\mathrm{d}\theta,$$

and

$$x^2 + y^2 = \alpha^2 t^2 + 2\alpha_1 tr\cos\theta + 2\alpha_2 tr\sin\theta + r^2.$$

When these expressions are substituted into (1), any terms involving $\cos\theta$ or $\sin\theta$ will vanish since $\int_0^{2\pi} \cos\theta \, \mathrm{d}\theta = \int_0^{2\pi} \sin\theta \, \mathrm{d}\theta = 0$. For simplification let $\gamma = 2Dt + 2\eta^2$, giving

$$\rho(t) = \alpha^2 t^2 \left(\frac{1}{2\pi\gamma} \int_0^{2\pi} \int_0^{\infty} r \exp\left(\frac{-r^2}{2\gamma}\right) \mathrm{d}r\mathrm{d}\theta\right) + \frac{1}{2\pi\gamma} \int_0^{2\pi} \int_0^{\infty} r^3 \exp\left(\frac{-r^2}{2\gamma}\right) \mathrm{d}r\mathrm{d}\theta.$$

The two integrals evaluate to give 1 and 2γ respectively and so substituting back in the original expression for γ we obtain

$$\rho(t) = \alpha^2 t^2 + 4Dt + 4\eta^2.$$

The variance of the square displacement is given by

$$\mathrm{Var}(|\boldsymbol{X}_t|^2) \equiv \mathbb{E}(|\boldsymbol{X}_t|^4) - (\mathbb{E}(|\boldsymbol{X}_t|^2))^2.$$

The latter term is simply the square of the MSD while the former term is calculated as

$$\mathbb{E}(|\boldsymbol{X}_t|^4) = \int_{\mathbb{R}^2} |\boldsymbol{x}|^4\, p(\boldsymbol{x},t)\,\mathrm{d}\boldsymbol{x}$$

$$= \frac{1}{2\pi\gamma}\int_{-\infty}^{\infty}\int_{-\infty}^{\infty}(x^2+y^2)^2\,\exp\left(\frac{-|\boldsymbol{x}-\boldsymbol{\alpha}t|^2}{2\gamma}\right)\,\mathrm{d}x\mathrm{d}y.$$

Using the same change of variables as before, let $x = \alpha_1 t + r\cos\theta$ and $y = \alpha_2 t + r\sin\theta$ for $0 \le r < \infty$ and $0 \le \theta \le 2\pi$, then $\mathrm{d}x\mathrm{d}y = r\,\mathrm{d}r\mathrm{d}\theta$ and

$$\begin{aligned}(x^2+y^2)^2 &= \alpha^4t^4 + 2\alpha^2\alpha_1 t^3 r\cos\theta + 2\alpha^2\alpha_2 t^3 r\sin\theta + \alpha^2t^2r^2\\ &+ 2\alpha^2\alpha_1 t^3 r\cos\theta + 4\alpha_1^2t^2r^2\cos^2\theta + 4\alpha_1\alpha_2t^2r^2\cos\theta\sin\theta\\ &+ 2\alpha_1 tr^3\cos\theta + 2\alpha^2\alpha_2t^3r\sin\theta + 4\alpha_1\alpha_2t^2r^2\cos\theta\sin\theta\\ &+ 4\alpha_2^2t^2r^2\sin^2\theta + 2\alpha_1 tr^3\sin\theta + \alpha^2t^2r^2 + 2\alpha_1tr^3\cos\theta\\ &+ 2\alpha_2tr^3\sin\theta + r^4.\end{aligned}$$

As previously, any terms involving $\cos\theta$, $\sin\theta$ and additionally $\sin\theta\cos\theta$ will be equal to zero once integrated. This leaves

$$\begin{aligned}\mathbb{E}(|\boldsymbol{X}_t|^4) &= \alpha^4t^4\left(\frac{1}{2\pi\gamma}\int_0^{2\pi}\int_0^{\infty} r\exp\left(\frac{-r^2}{2\gamma}\right)\mathrm{d}r\mathrm{d}\theta\right)\\ &+ 2\alpha^2t^2\left(\frac{1}{2\pi\gamma}\int_0^{2\pi}\int_0^{\infty} r^3\exp\left(\frac{-r^2}{2\gamma}\right)\mathrm{d}r\mathrm{d}\theta\right)\\ &+ 4\alpha_1^2t^2\left(\frac{1}{2\pi\gamma}\int_0^{2\pi}\int_0^{\infty} \cos^2\theta\, r^3\exp\left(\frac{-r^2}{2\gamma}\right)\mathrm{d}r\mathrm{d}\theta\right)\\ &+ 4\alpha_2^2t^2\left(\frac{1}{2\pi\gamma}\int_0^{2\pi}\int_0^{\infty} \sin^2\theta\, r^3\exp\left(\frac{-r^2}{2\gamma}\right)\mathrm{d}r\mathrm{d}\theta\right)\\ &+ \frac{1}{2\pi\gamma}\int_0^{2\pi}\int_0^{\infty} r^5\exp\left(\frac{-r^2}{2\gamma}\right)\mathrm{d}r\mathrm{d}\theta.\end{aligned}$$

These integrals are equal to 1, 2γ, γ, γ and $8\gamma^2$, respectively. Therefore, evaluating these integrals we obtain

$$\begin{aligned}\mathbb{E}(|\boldsymbol{X}_t|^4) &= \alpha^4t^4 + 4\alpha^2t^2\gamma + 4\alpha_1^2t^2\gamma + 4\alpha_2^2t^2\gamma + 8\gamma^2\\ &= \alpha^4t^4 + 8\alpha^2t^2\gamma + 8\gamma^2.\end{aligned}$$

Therefore, the variance of the square displacement is given by

$$\begin{aligned}\mathrm{Var}(|\boldsymbol{X}_t|^2) &= \mathbb{E}(|\boldsymbol{X}_t|^4) - (\mathbb{E}(|\boldsymbol{X}_t|^2))^2\\ &= \alpha^4t^4 + 8\alpha^2t^2\gamma + 8\gamma^2 - (\alpha^2t^2 + 2\gamma)^2\\ &= 4\alpha^2t^2\gamma + 4\gamma^2\\ &= 4\alpha^2t^2(2Dt + 2\eta^2) + 4(2Dt + 2\eta^2)^2. \qquad (2)\end{aligned}$$

2 Derivation of the variance of the MSD

The calculation of the variance of the MSD is trickier and uses properties of Weiner processes. First of all, we calculate the variance of the MSD for a single particle, denoted by $\sigma_n^{2(S)}$. Then, due to the independence of the N_S trajectories, the variance of the MSD for an ensemble of particles is given by

$$\sigma_n^2 = \frac{\sigma_n^{2(S)}}{N_S}.$$

By denoting

$$\rho_n^{(1)} = \frac{1}{N+1-n} \sum_{i=1}^{N+1-n} |\boldsymbol{x}_{i+n} - \boldsymbol{x}_i|^2, \quad n = 1, \ldots, N,$$

as the overlapping MSD for a single particle, then the variance of the MSD is defined as

$$\sigma_n^{2(S)} = \mathbb{E}((\rho_n^{(1)})^2) - (\mathbb{E}(\rho_n^{(1)}))^2.$$

Letting $K = N+1-n$ be the number of samples of squared displacements of length $n\Delta t$, then, due to the correlation between overlapping displacements, we have that

$$\begin{aligned}\sigma_n^{2(S)} &= \sum_{i=1}^{K}\sum_{j=1}^{K} \mathrm{Cov}(i,j) \\ &= \frac{\mathrm{Var}(|\boldsymbol{X}_t|^2|)(n\Delta t)}{K} + \frac{2}{K^2}\sum_{i=1}^{K}\sum_{j=i+1}^{K} \mathrm{Cov}(i,j), \quad (i<j) \qquad (3)\end{aligned}$$

where $\mathrm{Var}(|\boldsymbol{X}_t|^2|)(n\Delta t)$ is the variance of the squared displacement at time point $n\Delta t$ and

$$\mathrm{Cov}(i,j) = \mathbb{E}(|\boldsymbol{x}_{i+n} - \boldsymbol{x}_i|^2 |\boldsymbol{x}_{j+n} - \boldsymbol{x}_j|^2) - \mathbb{E}(|\boldsymbol{x}_{i+n} - \boldsymbol{x}_i|^2|)\mathbb{E}(|\boldsymbol{x}_{j+n} - \boldsymbol{x}_j|^2|)$$

is the covariance between squared displacements. The first term in (3) is known and so in order to calculate the variance of the MSD we need only to calculate the covariance of the squared displacements. For this we have to consider the different ways the displacements $|\boldsymbol{x}_{i+n} - \boldsymbol{x}_i|^2$ and $|\boldsymbol{x}_{j+n} - \boldsymbol{x}_j|^2$ can overlap. This can be split into two cases as shown in Figure 1, where $\boldsymbol{R}_1, \boldsymbol{R}_2$ and $\boldsymbol{R}_3$ are the displacements between the given vertices. We can then write the covariance as

$$(a) \quad \mathrm{Cov}(i,j) = \mathbb{E}(\boldsymbol{R}_1^2 \boldsymbol{R}_3^2) - \mathbb{E}(\boldsymbol{R}_1^2)\mathbb{E}(\boldsymbol{R}_3^2). \qquad j > i+n$$

$$(b) \quad \mathrm{Cov}(i,j) = \mathbb{E}((\boldsymbol{R}_1 + \boldsymbol{R}_2)^2(\boldsymbol{R}_2 + \boldsymbol{R}_3)^2) - \mathbb{E}((\boldsymbol{R}_1 + \boldsymbol{R}_2)^2)\,\mathbb{E}((\boldsymbol{R}_2 + \boldsymbol{R}_3)^2). \quad j \le i+n \qquad (4)$$

Notice that when $j > i+n$ (case (a) in Figure 1) the trajectories do not overlap and so $\boldsymbol{R}_2 = \boldsymbol{0}$. Since the remaining displacements $\boldsymbol{R}_1$ and $\boldsymbol{R}_3$ are independent then $\mathrm{Cov}(i,j) = 0$. Therefore, we need only consider the case when $j \le i+n$ (case (b) in Figure 1). In this case, we can write the displacement vectors as

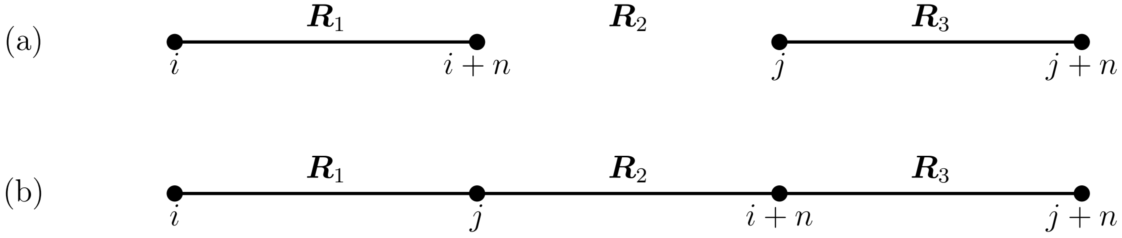

Figure 1: Diagram showing the two different combinations for overlapping displacements used to calculate the variance of the MSD.

$$
\begin{aligned}
\boldsymbol{R}_1 &= \boldsymbol{\alpha}\,\mathrm{d}t_1 + \sqrt{2D}\,\mathrm{d}\boldsymbol{W}_1 + \boldsymbol{\psi}_1,\\
\boldsymbol{R}_2 &= \boldsymbol{\alpha}\,\mathrm{d}t_2 + \sqrt{2D}\,\mathrm{d}\boldsymbol{W}_2 + \boldsymbol{\psi}_2,\\
\boldsymbol{R}_3 &= \boldsymbol{\alpha}\,\mathrm{d}t_3 + \sqrt{2D}\,\mathrm{d}\boldsymbol{W}_3 + \boldsymbol{\psi}_3,
\end{aligned}
$$

where $t_1 = t_j - t_i = (j-i)\Delta t$, $t_2 = t_{i+n} - t_j = (i+n-j)\Delta t$, $t_3 = t_{j+n} - t_{i+n} = (j-i)\Delta t$, $\mathrm{d}\boldsymbol{W}_1$, $\mathrm{d}\boldsymbol{W}_2$, $\mathrm{d}\boldsymbol{W}_3$ are Weiner increments over the time length t_1, t_2, t_3 respectively, and $\boldsymbol{\psi}_1 = \boldsymbol{\eta}_j - \boldsymbol{\eta}_i$, $\boldsymbol{\psi}_2 = \boldsymbol{\eta}_{i+n} - \boldsymbol{\eta}_j$, $\boldsymbol{\psi}_3 = \boldsymbol{\eta}_{j+n} - \boldsymbol{\eta}_{i+n}$, where $\boldsymbol{\eta}_i$ is the observational noise at time t_i, etc. Note that when $j = i+n$, we have that $\boldsymbol{R}_2 = \boldsymbol{0}$. Expanding the right hand side of equation $(4b)$ results in

$$
\begin{aligned}
\mathrm{Cov}(i,j) = {} & \mathbb{E}(|\boldsymbol{R}_1|^2|\boldsymbol{R}_2|^2) + 2\mathbb{E}(|\boldsymbol{R}_1|^2(\boldsymbol{R}_2\cdot\boldsymbol{R}_3)) + \mathbb{E}(|\boldsymbol{R}_1|^2|\boldsymbol{R}_3|^2)\\
& + 2\mathbb{E}(|\boldsymbol{R}_2|^2(\boldsymbol{R}_1\cdot\boldsymbol{R}_2)) + 4\mathbb{E}((\boldsymbol{R}_1\cdot\boldsymbol{R}_2)(\boldsymbol{R}_2\cdot\boldsymbol{R}_3)) + 2\mathbb{E}(|\boldsymbol{R}_3|^2(\boldsymbol{R}_1\cdot\boldsymbol{R}_2))\\
& + \mathbb{E}(|\boldsymbol{R}_2|^4) + 2\mathbb{E}(|\boldsymbol{R}_2|^2(\boldsymbol{R}_2\cdot\boldsymbol{R}_3)) + \mathbb{E}(|\boldsymbol{R}_2|^2|\boldsymbol{R}_3|^2)\\
& - \Big(\mathbb{E}(|\boldsymbol{R}_1|^2)\mathbb{E}(|\boldsymbol{R}_2|^2) + 2\mathbb{E}(|\boldsymbol{R}_1|^2)\mathbb{E}(\boldsymbol{R}_2\cdot\boldsymbol{R}_3) + \mathbb{E}(|\boldsymbol{R}_1|^2)\mathbb{E}(|\boldsymbol{R}_3|^2)\\
& + 2\mathbb{E}(|\boldsymbol{R}_2|^2)\mathbb{E}(\boldsymbol{R}_1\cdot\boldsymbol{R}_2) + 4\mathbb{E}(\boldsymbol{R}_1\cdot\boldsymbol{R}_2)\mathbb{E}(\boldsymbol{R}_2\cdot\boldsymbol{R}_3) + 2\mathbb{E}(|\boldsymbol{R}_3|^2)\mathbb{E}(\boldsymbol{R}_1\cdot\boldsymbol{R}_2)\\
& + (\mathbb{E}(|\boldsymbol{R}_2|^2))^2 + 2\mathbb{E}(|\boldsymbol{R}_2|^2)\mathbb{E}(\boldsymbol{R}_2\cdot\boldsymbol{R}_3) + \mathbb{E}(|\boldsymbol{R}_2|^2)\mathbb{E}(|\boldsymbol{R}_3|^2)\Big).
\end{aligned}
\tag{5}
$$

The first term is calculated as

$$
\begin{aligned}
& \mathbb{E}(|\boldsymbol{R}_1|^2|\boldsymbol{R}_2|^2)\\
& = \mathbb{E}(|\boldsymbol{\alpha}\,\mathrm{d}t_1 + \sqrt{2D}\,\mathrm{d}\boldsymbol{W}_1 + \boldsymbol{\psi}_1|^2|\boldsymbol{\alpha}\,\mathrm{d}t_2 + \sqrt{2D}\,\mathrm{d}\boldsymbol{W}_2 + \boldsymbol{\psi}_2|^2)\\
& = \mathbb{E}((|\boldsymbol{\alpha}|^2\mathrm{d}t_1^2 + 2\sqrt{2D}\mathrm{d}t_1(\boldsymbol{\alpha}\cdot\mathrm{d}\boldsymbol{W}_1) + 2\mathrm{d}t_1(\boldsymbol{\alpha}\cdot\boldsymbol{\psi}_1) + 2D|\mathrm{d}\boldsymbol{W}_1|^2 + 2\sqrt{2D}(\mathrm{d}\boldsymbol{W}_1\cdot\boldsymbol{\psi}_1) + |\boldsymbol{\psi}_1|^2)\\
& \qquad (|\boldsymbol{\alpha}|^2\mathrm{d}t_2^2 + 2\sqrt{2D}\mathrm{d}t_2(\boldsymbol{\alpha}\cdot\mathrm{d}\boldsymbol{W}_2) + 2\mathrm{d}t_2(\boldsymbol{\alpha}\cdot\boldsymbol{\psi}_2) + 2D|\mathrm{d}\boldsymbol{W}_2|^2 + 2\sqrt{2D}(\mathrm{d}\boldsymbol{W}_2\cdot\boldsymbol{\psi}_2) + |\boldsymbol{\psi}_2|^2)).
\end{aligned}
$$

Multiplying out the brackets and taking the expectation of each term gives

$$
\begin{aligned}
\mathbb{E}(|\boldsymbol{R}_1|^2|\boldsymbol{R}_2|^2) = {} & \alpha^2\mathbb{E}(\mathrm{d}t_1^2\mathrm{d}t_2^2) + 2\sqrt{2D}\alpha^2\mathbb{E}(\mathrm{d}t_1^2\mathrm{d}t_2(\boldsymbol{\alpha}\cdot\mathrm{d}\boldsymbol{W}_2)) + 2\alpha^2\mathbb{E}(\mathrm{d}t_1^2\mathrm{d}t_2(\boldsymbol{\alpha}\cdot\boldsymbol{\psi}_2)) \\
& + 2D\alpha^2\mathbb{E}(\mathrm{d}t_1^2|\mathrm{d}\boldsymbol{W}_2|^2) + 2\sqrt{2D}\alpha^2\mathbb{E}(\mathrm{d}t_1^2(\mathrm{d}\boldsymbol{W}_2\cdot\boldsymbol{\psi}_2)) + \alpha^2\mathbb{E}(\mathrm{d}t_1^2|\boldsymbol{\psi}_2|^2) \\
& + 2\sqrt{2D}\alpha^2\mathbb{E}(\mathrm{d}t_1\mathrm{d}t_2^2(\boldsymbol{\alpha}\cdot\mathrm{d}\boldsymbol{W}_1)) + 8D\mathbb{E}(\mathrm{d}t_1\mathrm{d}t_2(\boldsymbol{\alpha}\cdot\mathrm{d}\boldsymbol{W}_1)(\boldsymbol{\alpha}\cdot\mathrm{d}\boldsymbol{W}_2)) \\
& + 4\sqrt{2D}\mathbb{E}(\mathrm{d}t_1\mathrm{d}t_2(\boldsymbol{\alpha}\cdot\mathrm{d}\boldsymbol{W}_1)(\boldsymbol{\alpha}\cdot\boldsymbol{\psi}_2)) + 4D\sqrt{2D}\mathbb{E}(\mathrm{d}t_1|\mathrm{d}\boldsymbol{W}_2|^2(\boldsymbol{\alpha}\cdot\mathrm{d}\boldsymbol{W}_1)) \\
& + 8D\mathbb{E}(\mathrm{d}t_1(\boldsymbol{\alpha}\cdot\mathrm{d}\boldsymbol{W}_1)(\mathrm{d}\boldsymbol{W}_2\cdot\boldsymbol{\psi}_2)) + 2\sqrt{2D}\mathbb{E}(\mathrm{d}t_1|\boldsymbol{\psi}_2|^2(\boldsymbol{\alpha}\cdot\mathrm{d}\boldsymbol{W}_1)) \\
& + 2\alpha^2\mathbb{E}(\mathrm{d}t_1\mathrm{d}t_2^2(\boldsymbol{\alpha}\cdot\boldsymbol{\psi}_1)) + 4\sqrt{2D}\mathbb{E}(\mathrm{d}t_1\mathrm{d}t_2(\boldsymbol{\alpha}\cdot\boldsymbol{\psi}_1)(\boldsymbol{\alpha}\cdot\mathrm{d}\boldsymbol{W}_2)) \\
& + 4\mathbb{E}(\mathrm{d}t_1\mathrm{d}t_2(\boldsymbol{\alpha}\cdot\boldsymbol{\psi}_1)(\boldsymbol{\alpha}\cdot\boldsymbol{\psi}_2)) + 4D\mathbb{E}(\mathrm{d}t_1|\mathrm{d}\boldsymbol{W}_2|^2(\boldsymbol{\alpha}\cdot\boldsymbol{\psi}_1)) \\
& + 4\sqrt{2D}\mathbb{E}(\mathrm{d}t_1(\boldsymbol{\alpha}\cdot\boldsymbol{\psi}_1)(\mathrm{d}\boldsymbol{W}_2\cdot\boldsymbol{\psi}_2)) + 2\mathbb{E}(\mathrm{d}t_1|\boldsymbol{\psi}_2|^2(\boldsymbol{\alpha}\cdot\boldsymbol{\psi}_1)) \\
& + 2D\alpha^2\mathbb{E}(\mathrm{d}t_2^2|\mathrm{d}\boldsymbol{W}_1|^2) + 4D\sqrt{2D}\mathbb{E}(\mathrm{d}t_2|\mathrm{d}\boldsymbol{W}_1|^2(\boldsymbol{\alpha}\cdot\mathrm{d}\boldsymbol{W}_2)) \\
& + 4D\mathbb{E}(\mathrm{d}t_2|\mathrm{d}\boldsymbol{W}_1|^2(\boldsymbol{\alpha}\cdot\boldsymbol{\psi}_2)) + 4D^2\mathbb{E}(|\mathrm{d}\boldsymbol{W}_1|^2|\mathrm{d}\boldsymbol{W}_2|^2) \\
& + 4D\sqrt{2D}\mathbb{E}(|\mathrm{d}\boldsymbol{W}_1|^2(\mathrm{d}\boldsymbol{W}_2\cdot\boldsymbol{\psi}_2)) + 2D\mathbb{E}(|\mathrm{d}\boldsymbol{W}_1|^2|\boldsymbol{\psi}_2|^2) \\
& + 2\sqrt{2D}\alpha^2\mathbb{E}(\mathrm{d}t_2^2(\mathrm{d}\boldsymbol{W}_1\cdot\boldsymbol{\psi}_1)) + 8D\mathbb{E}(\mathrm{d}t_2(\mathrm{d}\boldsymbol{W}_1\cdot\boldsymbol{\psi}_1)(\boldsymbol{\alpha}\cdot\mathrm{d}\boldsymbol{W}_2)) \\
& + 4\sqrt{2D}\mathbb{E}(\mathrm{d}t_2(\mathrm{d}\boldsymbol{W}_1\cdot\boldsymbol{\psi}_1)(\boldsymbol{\alpha}\cdot\boldsymbol{\psi}_2)) + 4D\sqrt{2D}\mathbb{E}(|\mathrm{d}\boldsymbol{W}_2|^2(\mathrm{d}\boldsymbol{W}_1\cdot\boldsymbol{\psi}_1)) \\
& + 8D\mathbb{E}((\mathrm{d}\boldsymbol{W}_1\cdot\boldsymbol{\psi}_1)(\mathrm{d}\boldsymbol{W}_2\cdot\boldsymbol{\psi}_2)) + 2\sqrt{2D}\mathbb{E}(|\boldsymbol{\psi}_2|^2(\mathrm{d}\boldsymbol{W}_1\cdot\boldsymbol{\psi}_1)) \\
& + \alpha^2\mathbb{E}(\mathrm{d}t_2^2|\boldsymbol{\psi}_1|^2) + 2\sqrt{2D}\mathbb{E}(\mathrm{d}t_2|\boldsymbol{\psi}_1|^2(\boldsymbol{\alpha}\cdot\mathrm{d}\boldsymbol{W}_2)) \\
& + 2\mathbb{E}(\mathrm{d}t_2|\boldsymbol{\psi}_1|^2(\boldsymbol{\alpha}\cdot\boldsymbol{\psi}_2)) + 2D\mathbb{E}(|\mathrm{d}\boldsymbol{W}_2|^2|\boldsymbol{\psi}_1|^2) \\
& + 2\sqrt{2D}\mathbb{E}(|\boldsymbol{\psi}_1|^2(\mathrm{d}\boldsymbol{W}_2\cdot\boldsymbol{\psi}_2)) + \mathbb{E}(|\boldsymbol{\psi}_1|^2|\boldsymbol{\psi}_2|^2). \qquad (6)
\end{aligned}
$$

From here, we note that $\mathbb{E}(\mathrm{d}t_i) = t_i$ for $i \in \{1,2,3\}$, and since the Weiner processes are independent, then $(\mathrm{d}\boldsymbol{W}_i \cdot \mathrm{d}\boldsymbol{W}_j) = 0$ for $i \neq j$. Furthermore, since the Weiner processes and noise terms are independent then $(\mathrm{d}\boldsymbol{W}_i \cdot \boldsymbol{\psi}_j) = 0$ for $i, j \in \{1,2,3\}$. However, due to the noise terms containing variances of the observational noise which can share a common vertex, then $(\boldsymbol{\psi}_i \cdot \boldsymbol{\psi}_j) \neq 0$ for $|i-j| = 1$. Using these properties, many of the terms in (6) will be zero. Therefore, we are left with

$$
\begin{aligned}
\mathbb{E}(|\boldsymbol{R}_1|^2|\boldsymbol{R}_2|^2) = {} & \alpha^4 t_i^2 t_2^2 + 2D\alpha^2 t_1^2\mathbb{E}(|\mathrm{d}\boldsymbol{W}_2|^2) + \alpha^2 t_1^2\mathbb{E}(|\boldsymbol{\psi}_2|^2) + 4t_1t_2\mathbb{E}((\boldsymbol{\alpha}\cdot\boldsymbol{\psi}_1)(\boldsymbol{\alpha}\cdot\boldsymbol{\psi}_2)) \\
& + 2D\alpha^2 t_2^2\mathbb{E}(|\mathrm{d}\boldsymbol{W}_1|^2) + 4D^2\mathbb{E}(|\mathrm{d}\boldsymbol{W}_1|^2|\mathrm{d}\boldsymbol{W}_2|^2) + 2D\mathbb{E}(|\mathrm{d}\boldsymbol{W}_1|^2|\boldsymbol{\psi}_2|^2) \\
& + \alpha^2 t_2^2\mathbb{E}(|\boldsymbol{\psi}_1|^2) + 2D\mathbb{E}(|\mathrm{d}\boldsymbol{W}_2|^2|\boldsymbol{\psi}_1|^2) + \mathbb{E}(|\boldsymbol{\psi}_1|^2|\boldsymbol{\psi}_2|^2). \qquad (7)
\end{aligned}
$$

We have that $\mathrm{d}\boldsymbol{W}_i = (\mathrm{d}W_i^1, \mathrm{d}W_i^2)$ for $i \in \{1,2,3\}$, where $\mathrm{d}W_i^1, \mathrm{d}W_i^2$ are independent and identically distributed (i.i.d.) normal random variables with zero mean and variance $\sqrt{t_i}$, i.e. $\mathrm{d}W_i^1, \mathrm{d}W_i^2$ are of the form $\mathcal{N}(0, \sqrt{t_i})$. Similarly for the noise term, we have, for example, $\boldsymbol{\psi}_1 = \boldsymbol{\eta}_j - \boldsymbol{\eta}_i$, where $\boldsymbol{\eta}_i = (\eta_i^1, \eta_i^2)$ and η_i^1, η_i^2 are also i.i.d. random variables of the form $\mathcal{N}(0, \eta^2)$. These can be used to calculate the final expectation terms left in (7). This gives

$$\begin{aligned}\mathbb{E}(|\boldsymbol{R}_1|^2|\boldsymbol{R}_2|^2) &= \alpha^4 t_1^2 t_2^2 + 4D\alpha^2 t_1^2 t_2 + 4\alpha^2\eta^2 t_1^2 \\ &- 4\alpha^2\eta^2 t_1 t_2 + 4D\alpha^2 t_1 t_2^2 + 16D^2 t_1 t_2 + 16D\eta^2 t_1 \\ &+ 4\alpha^2\eta^2 t_2^2 + 16D\eta^2 t_2 + 20\eta^4.\end{aligned}$$

The rest of the expectations will not be shown here but use similar algebra. Instead we will simply present the results.

$$\begin{aligned}
&\mathbb{E}(\boldsymbol{R}_i \cdot \boldsymbol{R}_j) = \alpha^2 t_i t_j - 2\eta^2, && |i-j| = 1 \\
&\mathbb{E}(\boldsymbol{R}_1 \cdot \boldsymbol{R}_3) = \alpha^2 t_1 t_3, && \\
&\mathbb{E}(|\boldsymbol{R}_i|^2) = \alpha^2 t_i^2 + 4Dt_i + 4\eta^2, && i \in \{1,2,3\} \\
&\mathbb{E}(|\boldsymbol{R}_2|^4) = \alpha^4 t_2^4 + 16D\alpha^2 t_2^3 + 16\alpha^2\eta^2 t_2^2 && \\
&\qquad + 32D^2 t_2^2 + 64D\eta^2 t_2 + 32\eta^4, && \\
&\mathbb{E}(|\boldsymbol{R}_i|^2|\boldsymbol{R}_j|^2) = \alpha^4 t_i^2 t_j^2 + 4D\alpha^2 t_i^2 t_j + 4\alpha^2\eta^2 t_i^2 && \\
&\qquad - 4\alpha^2\eta^2 t_i t_j + 4D\alpha^2 t_i t_j^2 + 16D^2 t_i t_j + 16D\eta^2 t_i && \\
&\qquad + 4\alpha^2\eta^2 t_j^2 + 16D\eta^2 t_j + 20\eta^4 && |i-j| = 1 \\
&\mathbb{E}(|\boldsymbol{R}_1|^2|\boldsymbol{R}_3|^2) = \alpha^4 t_1^2 t_3^2 + 4D\alpha^2 t_1^2 t_3 + 4\alpha^2\eta^2 t_1^2 && \\
&\qquad + 4D\alpha^2 t_1 t_3^2 + 16D^2 t_1 t_3 + 16D\eta^2 t_1 + 4\alpha^2\eta^2 t_3^2 && \\
&\qquad + 16D\eta^2 t_3 + 16\eta^4 + (4\eta^4 - 4\alpha^2\eta^2 t_1 t_3)\delta_{j,\,i+n}, && \\
&\mathbb{E}(|\boldsymbol{R}_i|^2(\boldsymbol{R}_i \cdot \boldsymbol{R}_j)) = \alpha^4 t_i^3 t_j - 4\alpha^2\eta^2 t_i^2 + 8D\alpha^2 t_i^2 t_j && \\
&\qquad - 16D\eta^2 t_i + 8\alpha^2\eta^2 t_i t_j - 16\eta^4, && i \neq j \\
&\mathbb{E}(|\boldsymbol{R}_i|^2(\boldsymbol{R}_j \cdot \boldsymbol{R}_k)) = \alpha^4 t_i^2 t_j t_k + 4D\alpha^2 t_i t_j t_k + 4\alpha^2\eta^2 t_j t_k - 8D\eta^2 t_i && \\
&\qquad - 8\eta^4 - 2\alpha^2\eta^2 t_i^2 - 2\alpha^2\eta^2 t_i t_k, && i \neq j \neq k,\, i \neq 2 \\
&\mathbb{E}(|\boldsymbol{R}_2|^2(\boldsymbol{R}_1 \cdot \boldsymbol{R}_3)) = \alpha^4 t_1 t_2^2 t_3 + 4D\alpha^2 t_1 t_2 t_3 + 4\alpha^2\eta^2 t_1 t_3 && \\
&\qquad + 4\eta^4 - 2\alpha^2\eta^2 t_1 t_3 - 2\alpha^2\eta^2 t_2 t_3, && \\
&\mathbb{E}((\boldsymbol{R}_1 \cdot \boldsymbol{R}_2)(\boldsymbol{R}_2 \cdot \boldsymbol{R}_3)) = \alpha^4 t_1 t_2^2 t_3 - 3\alpha^2\eta^2 t_1 t_2 - 3\alpha^2\eta^2 t_2 t_3 && \\
&\qquad + 2\alpha^2\eta^2 t_1 t_3 + 2D\alpha^2 t_1 t_2 t_3 + 6\eta^4. && (8)
\end{aligned}$$

Here,

$$\delta_{j,\,i+n} = \begin{cases} 0 & j \neq i+n \\ 1 & j = i+n \end{cases}$$

represents the Kronecker delta function. In the absense of drift we recover the same expressions as found in the supplementary material of Michalet's paper [?]. Substituting these results into (5) and simplifying yields

$$\text{Cov}(i,j) \equiv \text{Cov}(l) = 16D^2((n-l)\Delta t)^2 + 8D\alpha^2 n^2 (n-l)(\Delta t)^3 + (4\eta^4 - 4(\alpha\eta n\Delta t)^2)\delta_{j,\,i+n}, \quad (9)$$

where $l = j - i$. Now that the covariance has been calculated, to obtain the variance of the MSD we must sum all the non-zero terms appearing in the double sum in (3). This can be split into two cases. When $n \leq K$ we sum the non-zero terms along the diagonals $l = 1, ..., n-1$ of the covariance matrix. The lth diagonal contains $K - l$ identical elements, and therefore the non-zero terms are

$$\sum_{l=1}^{n-1} (K-l)\text{Cov}(l), \quad (10)$$

where $\text{Cov}(l) = 16D^2((n-l)\Delta t)^2 + 8D\alpha^2 n^2(n-l)(\Delta t)^3$. When $l = n$ this corresponds to the case when $j = i + n$ and so the Kronecker delta term will now be non-zero. This diagonal will contain $K - n = N + 1 - 2n$ elements, and so will equal $(N + 1 - 2n)(4\eta^4 - 4(\alpha\eta n\Delta t)^2)$. When $n > K$ we don't have any segments with no overlaps. Therefore, we only have overlapping contributions to the covariance matrix. This time the upper limit in the sum (10) is $N - n$ rather than $n - 1$. So when $n > K$ we have

$$\sum_{l=1}^{N-n} (K-l)\text{Cov}(l),$$

where $\text{Cov}(l) = 16D^2((n-l)\Delta t)^2 + 8D\alpha^2 n^2(n-l)(\Delta t)^3$. Summing all these terms in either case ($n \leq K$ and $n > K$) as above allows us to calculate σ_n^2. It can be shown that the final result is

$$\sigma_n^2 = \begin{cases} \Big[\frac{n}{6K^2}(4n^2K + 2K - n^3 + n)16D^2(\Delta t)^2 \\ +8\alpha^2 D(\Delta t)^3 \left(\frac{n^3}{3K^2}(3Kn + 1 - n^2)\right) & n \leq K \\ +\frac{8\eta^2}{K^2}\left((K-n)(\eta^2 - \alpha^2(n\Delta t)^2) + K\left(\alpha^2(n\Delta t)^2 + 4Dn\Delta t + 2\eta^2\right)\right)\Big] / N_S \\ \\ \Big[\frac{1}{6K}(6n^2K - 4nK^2 + 4n + K^3 - K)16D^2(\Delta t)^2 \\ +8\alpha^2 D(\Delta t)^3 \left(\frac{n^2}{3K}(3nK - K^2 + 1)\right) & n > K \\ +\frac{8\eta^2}{K}(\alpha^2(n\Delta t)^2 + 4Dn\Delta t + 2\eta^2)\Big] / N_S. \end{cases} \quad (11)$$

In Figure 2 we plot the theoretical variance of the MSD (11) with an empirical estimate averaged over 1000 and 10,000 samples for the parameter values $D = 2\,\mu m^2/s$, $\alpha = 1\,\mu m/s$, $\eta = 2\,\mu m$, $N_S = 10$, $N = 100$ and $T = 100\,s$. We can see that as we increase the number of samples, the empirical estimate gets closer to the theoretical expression (11).

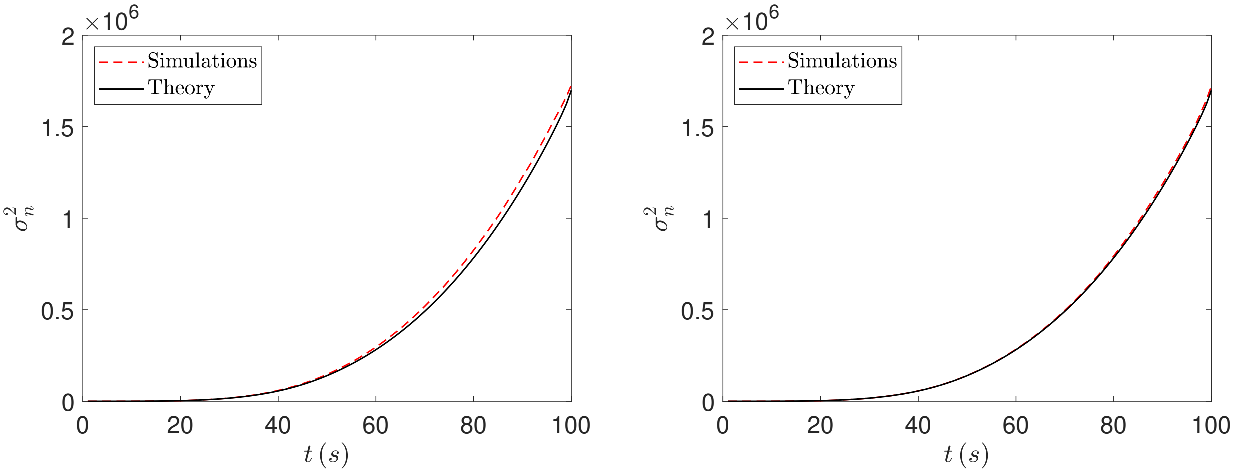

Figure 2: A plot of the theoretical variance of the MSD (11) (— solid black line) with an empirical estimate (- - - dashed red line) averaged over 1000 (left) and 10,000 (right) samples for $D = 2\,\mu m^2/s$, $\alpha = 1\,\mu m/s$, $\eta = 2\,\mu m$, $N_S = 10$, $N = 100$ and $T = 100\,s$.

3 Derivation of the covariance of the MSD

A theoretical expression for the covariance is needed to calculate the uncertainties in the regression parameters. As before, we consider the covariance of the MSD for a single particle, denoted by $\sigma_{nm}^{2(S)}$. Then, the covariance of the MSD for an ensemble of particles will be

$$\sigma_{nm}^2 = \frac{\sigma_{nm}^{2(S)}}{N_S}.$$

From the definition of the covariance, we have that

$$\sigma_{nm}^{2(S)} = \mathbb{E}(\rho_n^{(1)}\rho_m^{(1)}) - \mathbb{E}(\rho_n^{(1)})\mathbb{E}(\rho_m^{(1)}).$$

The process of calculating the covariance is similar to that of deriving the formula for the variance of the MSD. First, we assume that $m > n$, then letting $K = N+1-n$ and $P = N+1-m$ we have

$$\sigma_{nm}^{2(S)} = \frac{1}{KP}\sum_{i=1}^{K}\sum_{j=1}^{P}\mathrm{Cov}(i,j),$$

where

$$\mathrm{Cov}(i,j) = \mathbb{E}(|\boldsymbol{x}_{i+n} - \boldsymbol{x}_i|^2|\boldsymbol{x}_{j+m} - \boldsymbol{x}_j|^2) - \mathbb{E}(|\boldsymbol{x}_{i+n} - \boldsymbol{x}_i|^2)\mathbb{E}(|\boldsymbol{x}_{j+m} - \boldsymbol{x}_j|^2).$$

As before, we have to consider the different ways the trajectories can overlap. This time we consider the five cases as shown in Figure 3. The covariance for each case can be re-written as

(a) $\mathrm{Cov}(i,j) = \mathbb{E}(\boldsymbol{R}_1^2\boldsymbol{R}_3^2) - \mathbb{E}(\boldsymbol{R}_1^2)\mathbb{E}(\boldsymbol{R}_3^2)$ $\quad i < i+n < j < j+m$

(b) $\mathrm{Cov}(i,j) = \mathbb{E}((\boldsymbol{R}_1+\boldsymbol{R}_2)^2(\boldsymbol{R}_2+\boldsymbol{R}_3)^2) - \mathbb{E}((\boldsymbol{R}_1+\boldsymbol{R}_2)^2)\,\mathbb{E}((\boldsymbol{R}_2+\boldsymbol{R}_3)^2).$ $\quad i < j \le i+n < j+m$

(c) $\mathrm{Cov}(i,j) = \mathbb{E}(|\boldsymbol{R}_2^2|(\boldsymbol{R}_1+\boldsymbol{R}_2+\boldsymbol{R}_3)^2) - \mathbb{E}(|\boldsymbol{R}_2^2|)\mathbb{E}((\boldsymbol{R}_1+\boldsymbol{R}_2+\boldsymbol{R}_3)^2)$ $\quad j \le i < i+n < j+m$

(d) $\mathrm{Cov}(i,j) = \mathbb{E}((\boldsymbol{R}_1+\boldsymbol{R}_2)^2(\boldsymbol{R}_2+\boldsymbol{R}_3)^2) - \mathbb{E}((\boldsymbol{R}_1+\boldsymbol{R}_2)^2)\,\mathbb{E}((\boldsymbol{R}_2+\boldsymbol{R}_3)^2)$ $\quad j < i < j+m \le i+n$

(e) $\mathrm{Cov}(i,j) = \mathbb{E}(\boldsymbol{R}_1^2\boldsymbol{R}_3^2) - \mathbb{E}(\boldsymbol{R}_1^2)\mathbb{E}(\boldsymbol{R}_3^2)$ $\quad j < j+m \le i < i+n$

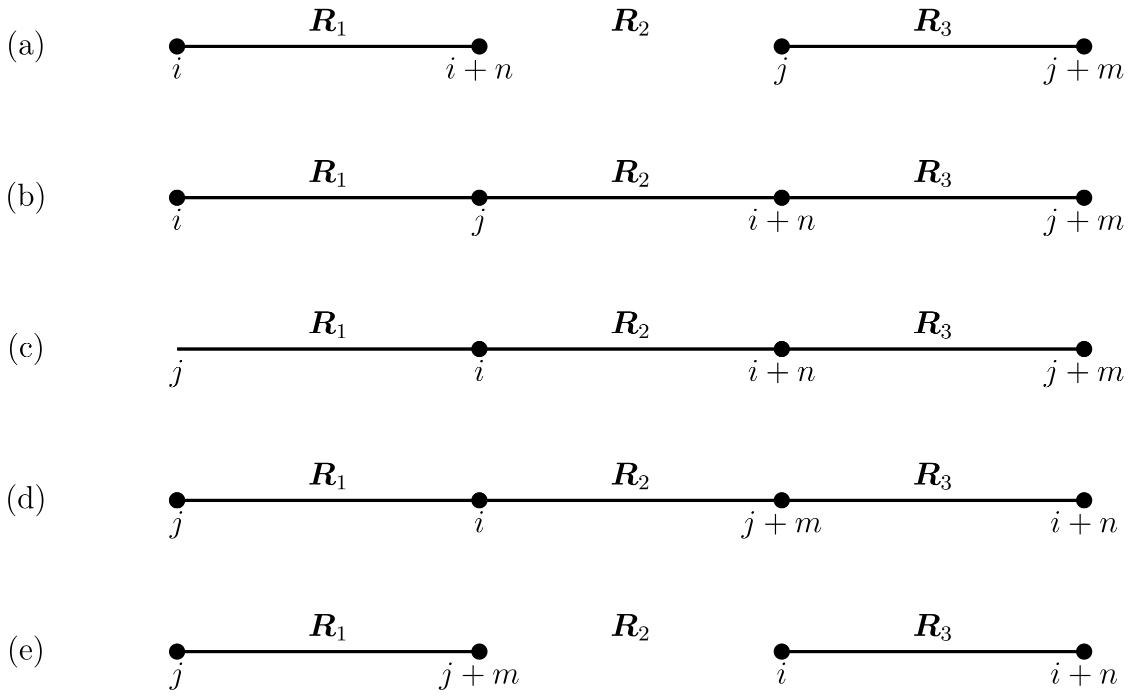

Figure 3: Diagram showing the five different combinations for overlapping displacements used to calculate the covariance of the MSD.

These expectations can be calculated using the results of (8). From this we obtain

$$(a) \quad \text{Cov}(l_1) = 0$$

$$(b) \quad \text{Cov}(l_2) = 16D^2((n-l_2)\Delta t)^2 + 8D\alpha^2 mn(n-l_2)(\Delta t)^3 + (4\eta^4 - 4\alpha^2\eta^2 mn(\Delta t)^2)\delta_{j,\,i+n}, \text{ where } l_2 = j - i.$$

$$(c) \quad \text{Cov}(l_3) = 16D^2(n\Delta t)^2 + 8D\alpha^2 n^2 m(\Delta t)^3 + (16D\eta^2 n\Delta t + 4\eta^4 + 4\alpha^2\eta^2 mn(\Delta t)^2)\delta_{i,j}.$$

$$(d) \quad \text{Cov}(l_4) = 16D^2(l_4\Delta t)^2 + 8D\alpha^2 mnl_4(\Delta t)^3 + (16D\eta^2 n\Delta t + 4\eta^4 + 4\alpha^2\eta^2 mn(\Delta t)^2)\delta_{i+n,\,j+m}, \text{ where } l_4 = j + m - i.$$

$$(e) \quad \text{Cov}(l_5) = (4\eta^4 - 4\alpha^2\eta^2 mn(\Delta t)^2)\delta_{i,\,j+m}.$$

We need to sum these different covariance values to obtain the overall covariance of the MSD in the two cases $m+n \leq N$ and $m+n > N$. As before, this is done by summing the non-zero terms along diagonals in the covariance matrix. Also, note the Kronecker delta terms must be

considered separately. The sums are done as follows

$m + n \leq N$

(a) 0.

(b) $\left(\sum_{l_2=1}^{n-1}(P - l_2)\mathrm{Cov}(l_2)\right) + (P - n)(4\eta^4 - 4\alpha^2\eta^2 mn(\Delta t)^2).$

(c) $\left(\sum_{l_3=1}^{m-n+1} P\mathrm{Cov}(l_3)\right) + P(16D\eta^2 n\Delta t + 4\eta^4 + 4\alpha^2\eta^2 mn(\Delta t)^2).$

(d) $\left(\sum_{l_4=1}^{n-1}(P - (n-1))\mathrm{Cov}(l_4)\right) + P(16D\eta^2 n\Delta t + 4\eta^4 + 4\alpha^2\eta^2 mn(\Delta t)^2).$

(e) $(P - n)(4\eta^4 - 4\alpha^2\eta^2 mn(\Delta t)^2).$

$m + n > N$

(a) 0.

(b) $\left(\sum_{l_2=1}^{P-1}(P - l_2)\mathrm{Cov}(l_2)\right) + (P - n)(4\eta^4 - 4\alpha^2\eta^2 mn(\Delta t)^2).$

(c) $\left(\sum_{l_3=1}^{m-n+1} P\mathrm{Cov}(l_3)\right) + P(16D\eta^2 n\Delta t + 4\eta^4 + 4\alpha^2\eta^2 mn(\Delta t)^2).$

(d) $\left(\sum_{l_4=n+1-P}^{n-1}(P - (n-l_4))\mathrm{Cov}(l_4)\right) + P(16D\eta^2 n\Delta t + 4\eta^4 + 4\alpha^2\eta^2 mn(\Delta t)^2).$

(e) 0.

We used the software Maple to evaluate these sums. Finally, we obtain

$$\sigma^2_{nm} = \begin{cases} \Big[\frac{16nD^2(\Delta t)^2}{6KP}(-n^3 - 2Pn^2 + (1 - 6m^2 + 6(N+1)m)n + 2P) \\ +\frac{8\alpha^2(\Delta t)^3 mn^2 D}{3KP}(-n^2 + 3(-m^2 + (N+1)m + 1/3)) + \frac{32\eta^2 nD\Delta t}{K} \\ +\frac{8\eta^4(-n+2P)}{KP} + \frac{8\alpha^2(\Delta t)^2 mn^2\eta^2}{KP}\Big] /N_S & m + n \leq N \\ \\ \Big[\frac{8D^2(\Delta t)^2}{3K}(-m^3 + (3 + 3N - 4n)m^2 + (8(N+1)n - 2 - 3N^2 - 6N)m \\ -6n^3 + 6(N+1)n^2 - (4N^2 + 8N)n + N(N+2)(N+1)) \\ +\frac{8\alpha^2 D(\Delta t)^3 mn}{3K}(m^2 - 2(N+1)m + 3n^2 - 3(N+1)nN^2 + 2N) & m + n > N \\ +\frac{8\eta^2}{K}(\alpha^2(\Delta t)^2 mn + 4Dn\Delta t + \eta^2)\Big] /N_S, \end{cases} \tag{12}$$

Figure 4 shows the comparison between the theoretical covariance of the MSD (12) with its empirical estimate, along with a cross section of the covariance of the MSD averaged over 1000 and 10,000 samples, for the parameters values $D = 2\,\mu m^2/s$, $\alpha = 1\,\mu m/s$, $\eta = 2\,\mu m$, $N_S = 10$, $N = 100$ and $T = 100\,s$. Note that we only provide one plot of the full covariance because the plots for the two different number of particles are very similar. Again, we see good agreement between the theoretical covariance of the MSD (12) and its empirical estimate.

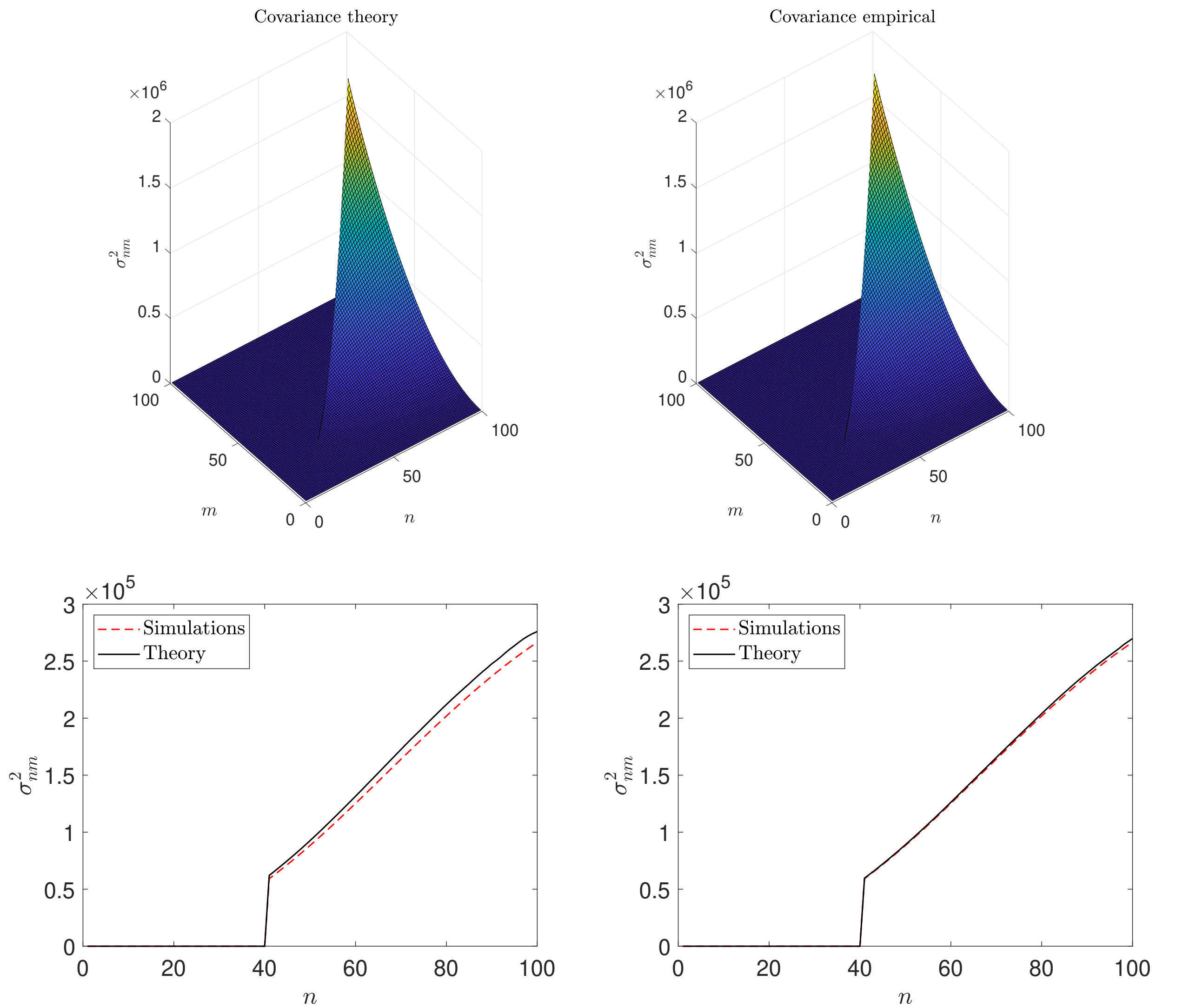

Figure 4: A plot comparing the theoretical covariance of the MSD (12) (top left) and its empirical estimate (top right) averaged over 1000 samples, along with a plot of a cross section along $m = 40$ of the theoretical covariance of the MSD (— solid black line) with its empirical estimate (- - - dashed red line) averaged over 1000 (bottom left) and 10,000 (bottom right) samples for $D = 2\,\mu m^2/s$, $\alpha = 1\,\mu m/s$, $\eta = 2\,\mu m$, $N_S = 10$, $N = 100$ and $T = 100\,s$. Note that the jump in the bottom plots are a consequence of only calculating the upper triangular part of the covariance matrix.

4 Further fitting points experiments

4.1 Existence of p_{opt} for different parameter values

In the main paper, all ensemble-averaged calculations used $N_S = 10$ trajectories calculated at $N = 100$ time points. To test the robustness of our calculations, we now test with $N_S = 100$ and $N = 10$. Figure 5 shows the theoretical and simulated uncertainty $\sigma_b/b + \sigma_c/c$ as a function of the number of fitting points. These experiments were for $D = 2\,\mu m^2/s$, $\alpha = 1\,\mu m/s$, $\eta = 2\,\mu m$, $N_S = 100$ and $N = 10$, with $\Delta t = 1\,s$ giving $T = 10\,s$ for the left plot, while $\Delta t = 10\,s$ giving $T = 100\,s$ for the right plot. Here we see a similar result as that in Figure 2 in the main paper. We have that, for a small value of Δt, the optimal estimate of the parameters comes from fitting with all the MSD points. As Δt is increased, we see that the optimal inference comes from fitting with a subset of points; specifically, for all the cases tested, $p_{opt} = 9$.

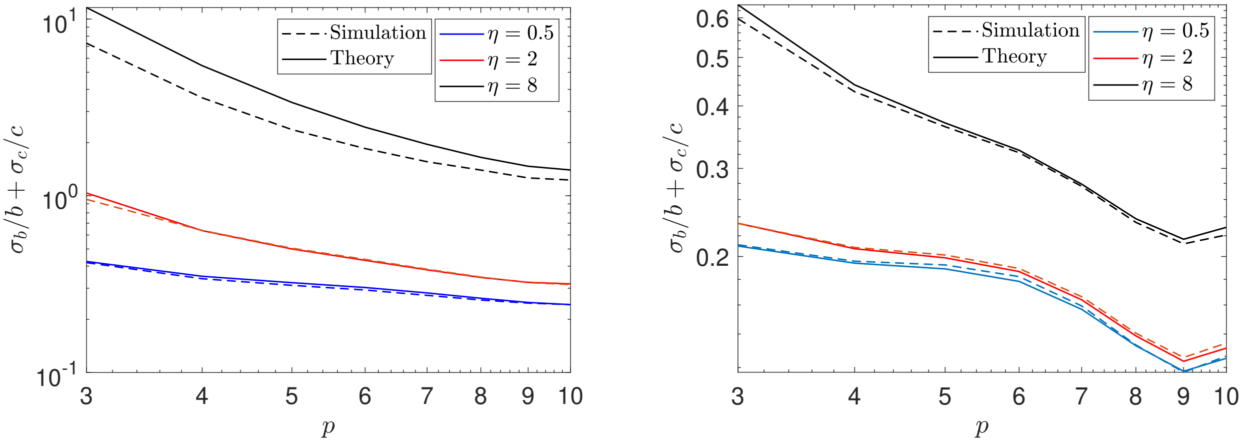

Figure 5: A plot of the theoretical value of $\sigma_b/b + \sigma_c/c$ (—— solid lines) and its empirically estimated value using 1000 samples (- - - dashed lines) when fit with the first p MSD points for $\eta = 0.5\,\mu m$, $2\,\mu m$ and $8\,\mu m$. These experiments were for $D = 2\,\mu m^2/s$, $\alpha = 1\,\mu m/s$, $N_S = 100$ and $N = 10$, for $\Delta t = 1\,s$ giving $T = 10\,s$ (left), and $\Delta t = 10\,s$ giving $T = 100\,s$ (right). For $\eta = 0.5\,\mu m$, $2\,\mu m$ and $8\,\mu m$, the optimal number of fitting points are 10 for each (left) and 9 for each (right).

We also looked for the existence of an optimal number of fitting points when inferring the diffusion coefficient D, the drift magnitude α and the observational noise η. Analogous to the two parameter case, we look to minimise the uncertainty $\sigma_a/a + \sigma_b/b + \sigma_c/c$ since the noise term is related to the regression coefficient a. Figure 6 shows the theoretical and simulated value of $\sigma_a/a + \sigma_b/b + \sigma_c/c$ as a function of the number of fitting points p for $D = 2\,\mu m^2/s$, $\alpha = 1\,\mu m/s$, $\eta = 2\,\mu m$, $N_S = 10$ and $N = 100$, with $\Delta t = 1\,s$ giving $T = 100\,s$ for the left plot, while $\Delta t = 10\,s$ giving $T = 1000\,s$ for the right plot. Here we see that the level of uncertainty, as well as the optimal number of fitting points, cannot be easily predicted by the value of η.

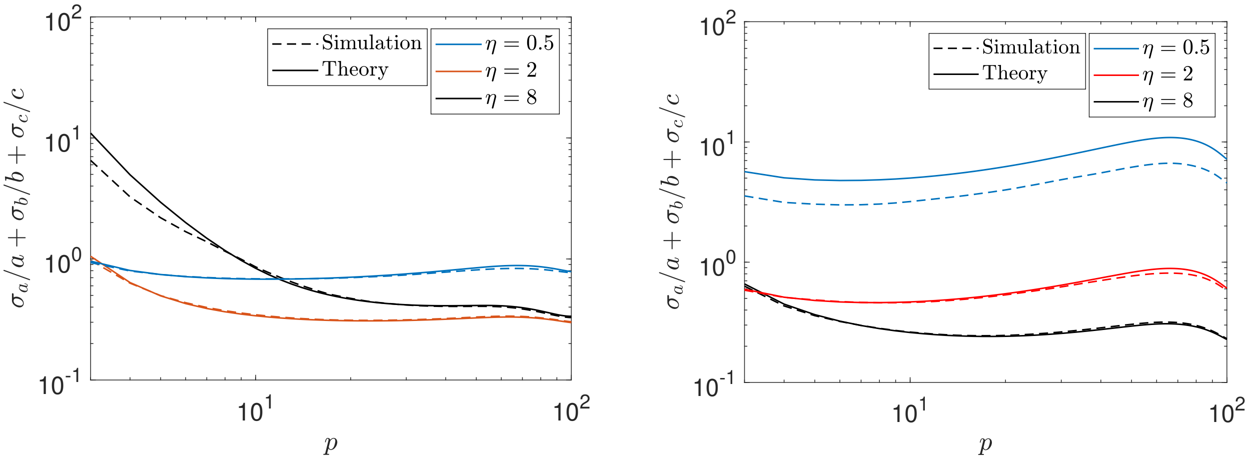

Figure 6: A plot of the theoretical value of $\sigma_a/a+\sigma_b/b+\sigma_c/c$ (—— solid lines) and its empirically estimated value using 1000 samples (- - - dashed lines) when fit with the first p MSD points for $\eta = 0.5\,\mu m$, $2\,\mu m$ and $8\,\mu m$. These experiments were for $D = 2\,\mu m^2/s$, $\alpha = 1\,\mu m/s$, $N_S = 10$ and $N = 100$, for $\Delta t = 1\,s$ giving $T = 100\,s$ (left), and $\Delta t = 10\,s$ giving $T = 1000\,s$ (right). For $\eta = 0.5\,\mu m$, $2\,\mu m$ and $8\,\mu m$, the optimal number of fitting points are 11, 100 and 100, respectively (left) and 6, 8 and 100, respectively (right).

4.2 MATLAB routine to calculate optimal number of fitting points

```
%%%%%%%%%%%%%%%%%%%%%%%%%%%%%%%%%%%%%%%%%%%%%%%%%%%%%%%%%%%%%%%%%%%%%%%%%%%%%%%
%                                                                            %
% Code to calculate the optimal number of fitting points for the             %
% overlapping time—averaged mean—square displacement (MSD).                  %
%                                                                            %
%%%%%%%%%%%%%%%%%%%%%%%%%%%%%%%%%%%%%%%%%%%%%%%%%%%%%%%%%%%%%%%%%%%%%%%%%%%%%%%


%
% Input:     D — Estimate of the diffusion coefficient.
%           al — Estimate of the drift magnitude.
%          eta — Estimate of the observational noise.
%           dt — The time interval between frames.
%            N — The number of time points.
%
%
% Output:  popt — The optimal number of fitting points for the given
%                 parameter values.
%
%
% Notes —  This code works for the overlapping time—averaged MSD
%          calculated both from a single particle and an ensemble of
%          particles as they have the same optimal number of fitting
%          points.
%
```

```
function [popt] = optimal_preg(D,al,eta,dt,N)

T=N*dt;
alp=4*D*dt;
ep=4*eta^2;

%
% Calculate the variance of the MSD
%

for n=1:N
    t(n)=n*dt;
    K=N+1-n;
    if n<=K
        varmsd(n)=(n*(4*n^2*K+2*K-n^3+n)/(6*K^2))*(4*D*dt)^2+...
            8*al^2*D*(dt)^3*(n^3*(3*K*n+1-n^2)/(3*K^2));
        varmsd(n)=varmsd(n)+((8*eta^2)/(K^2))*((K-n)*(eta^2-al^2*(n*dt)^2)+...
            K*(al^2*(n*dt)^2+4*D*n*dt+2*eta^2));
    else
        varmsd(n)=((6*n^2*K-4*n*K^2+4*n+K^3-K)/(6*K))*((4*D*dt)^2)+...
            8*al^2*D*(dt)^3*(n^2*(3*n*K+1-K^2))/(3*K);
        varmsd(n)=varmsd(n)+8*eta^2*(al^2*(n*dt)^2+4*D*n*dt+2*eta^2)/K;
    end
end

%
% Calculate the covariance of the MSD
%

for n=1:N
    for m=1:N
        K=N-n+1;
        P=N-m+1;
        if m+n<=N
            covarmsd(n,m)=((alp^2*n)/(6*K*P))*(-n^3-2*P*n^2+(1-6*m^2+...
                (6*N+6)*m)*n+2*P)+2*ep*n*alp/K+...
                ((8*al^2*dt^3*m*n^2*D)/(3*K*P))*(-n^2+3*(-m^2+(N+1)*m+1/3))+...
                ((-n+2*P)*ep^2)/(2*K*P)+...
                (2*al^2*dt^2*m*n^2*ep)/(K*P);
        else
            covarmsd(n,m)=((alp^2)/(6*K))*(-m^3+(3+3*N-4*n)*m^2+...
                ((8+8*N)*n-2-3*N^2-6*N)*m-6*n^3+(6+6*N)*n^2-...
                (4*N^2+8*N)*n+N*(N+2)*(N+1))-...
                ((8*al^2*dt^3*m*n*D)/(3*K))*(m^2-2*(N+1)*m+...
                (3*n^2-3*(N+1)*n+N^2+2*N))+...
                (2*(al^2*dt^2*m*n+n*alp)*ep+ep^2/2)/K;
```

```
        end
    end
end

for n=1:N
    for m=1:n
        covarmsd(n,m)=0;
    end
end

%
% Calculate the variance of the regression coefficients
%

for p=3:N
    s0over=0;s1over=0;s2over=0;s3over=0;s4over=0;
    for n=1:p
        tn=(n*T/N);
        variover=varmsd(n);
        s0over=s0over+1/variover;
        s1over=s1over+tn/variover;
        s2over=s2over+tn^2/variover;
        s3over=s3over+tn^3/variover;
        s4over=s4over+tn^4/variover;
    end

    del_over=s0over*s2over*s4over-s0over*(s3over)^2-...
        (s1over)^2*s4over+2*s1over*s2over*s3over-(s2over)^3;

    vara_over(p)=0;
    varb_over(p)=0;
    varc_over(p)=0;

    for n=1:p
        tn=(n*T/N);
        variover=varmsd(n);
        da_over(n)=(s2over*s4over-s3over^2-s1over*s4over*tn+...
            s1over*s3over*tn^2+s2over*s3over*tn-s2over^2*tn^2)/(del_over*
               variover);
        db_over(n)=(s0over*s4over*tn-s0over*s3over*tn^2-...
            s1over*s4over+s2over*s3over+s1over*s2over*tn^2-s2over^2*tn)/(
               del_over*variover);
        dc_over(n)=(s0over*s2over*tn^2-s0over*s3over*tn-...
            s1over^2*tn^2+s1over*s2over*tn+s1over*s3over-s2over^2)/(del_over*
               variover);
        vara_over(p)=vara_over(p)+variover*da_over(n)^2;
        varb_over(p)=varb_over(p)+variover*db_over(n)^2;
```

```
        varc_over(p)=varc_over(p)+variover*dc_over(n)^2;
        if n>1
            for m=1:n-1
                vara_over(p)=vara_over(p)+2*covarmsd(m,n)*da_over(n)*da_over(m)
                    ;
                varb_over(p)=varb_over(p)+2*covarmsd(m,n)*db_over(n)*db_over(m)
                    ;
                varc_over(p)=varc_over(p)+2*covarmsd(m,n)*dc_over(n)*dc_over(m)
                    ;
            end
        end
    end
end

%
% Calculate the optimal number of fitting points
%

[~,I]=min(sqrt(varb_over(3:N))/(4*D)+sqrt(varc_over(3:N))/(al*al));
popt=I+2;

end
```

4.3 Additional iterative algorithm experiments for p_{opt}

The iterative algorithm was also tested for different values of D and α to check the robustness of the algorithm. First we test for $D = 2\,\mu m^2/s$, $\alpha = 7\,\mu m/s$, $\eta = 2\,\mu m$, $N_S = 10$ and $N = 1000$, for $\Delta t = 1\,s$, $\Delta t = 10\,s$ and $\Delta t = 100\,s$. The results are shown in Figure 7. These are similar to the case shown in the main paper. We see that the value of $\langle p_i \rangle$ converges to p_{opt} in no more than three iterations for all the cases shown. The value of $\langle |\alpha_i/\alpha - 1| \rangle$ does not decrease between iterations but decreases as Δt increases. The value of $\langle |D_i/D - 1| \rangle$ decreases when we fit with p_{opt} points rather than all the points, and the final value of $\langle |D_i/D - 1| \rangle$ decreases very slightly from $\Delta t = 1\,s$ to $\Delta t = 10\,s$ and then increases again from $\Delta t = 10\,s$ to $\Delta t = 100\,s$.

The algorithm was also tested for the same parameter values given above but for $D = 6\,\mu m^2/s$ and $\alpha = 1\,\mu m/s$. The results are shown in Figure 8. Again, the value of $\langle p_i \rangle$ converges quickly to p_{opt}, in this case, after 2 iterations each time. The value of $\langle |\alpha_i/\alpha - 1| \rangle$ remains roughly the same between iterations but again decreases as Δt increases. Here, the value of $\langle |D_i/D - 1| \rangle$ decreases after one iteration in each case but the final value of $\langle |D_i/D - 1| \rangle$ does not change much between Δt values. Although not shown here, if the value of Δt was further increased then the value of $\langle |D_i/D - 1| \rangle$ would begin to increase again.

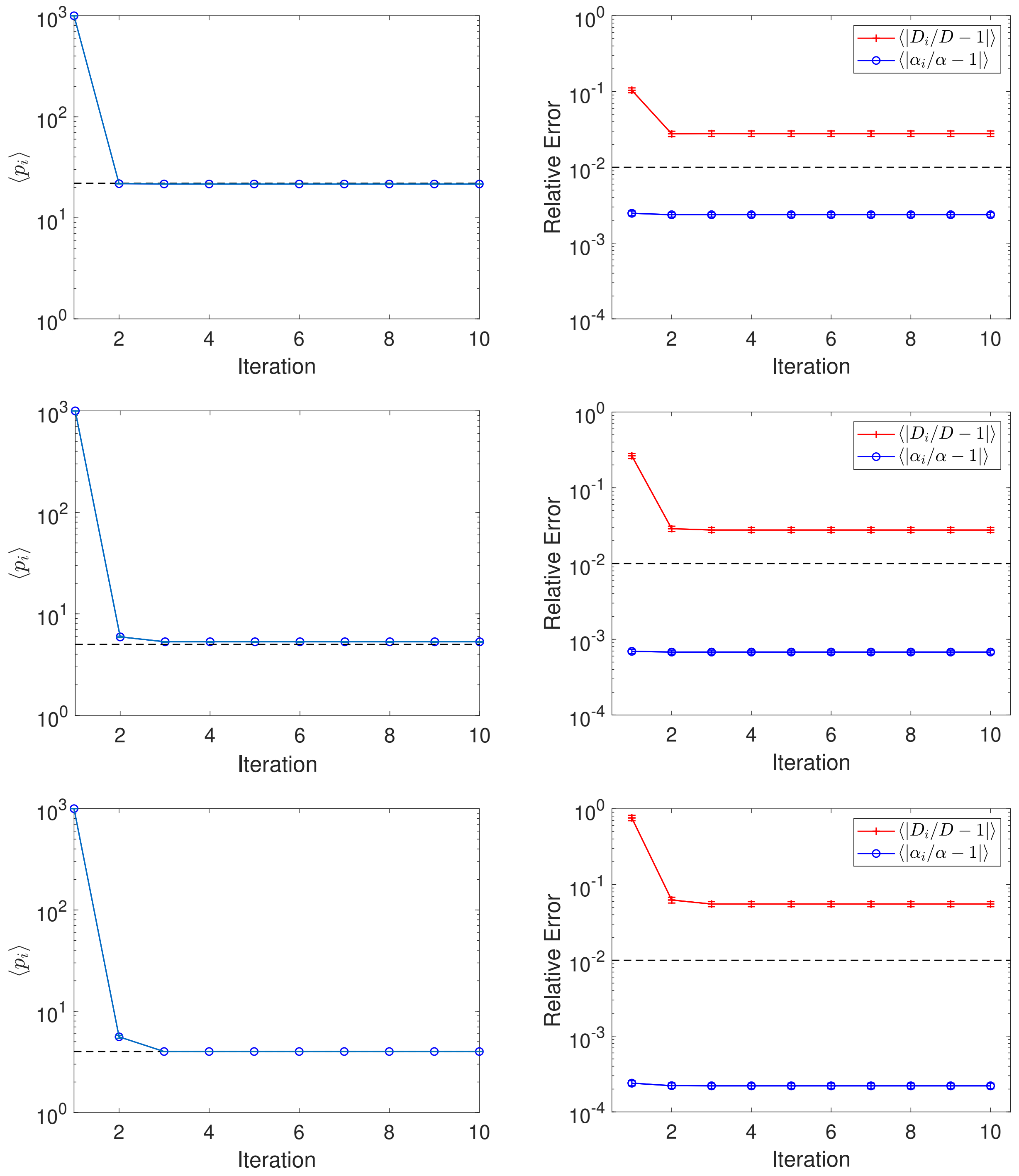

Figure 7: A plot of the value of $\langle p_i \rangle$ for each iteration with standard error bars (left plot in each row), along with a plot of the value of $\langle |D_i/D - 1| \rangle$ (+ red crosses) and $\langle |\alpha_i/\alpha - 1| \rangle$ (○ blue circles) for each iteration with standard error bars (right plot in each row). These experiments were for $D = 2\,\mu m^2/s$, $\alpha = 7\,\mu m/s$, $\eta = 2\,\mu m$, $N_S = 10$ and $N = 1000$. The dashed line in each left plot corresponds to $p_{opt} = 22$ (top), $p_{opt} = 5$ (middle), and $p_{opt} = 4$ (bottom), while the dashed line in each right plot corresponds with the value 10^{-2}, indicating a 1% error. These correspond with $\Delta t = 1\,s$ (top), $\Delta t = 10\,s$ (middle) and $\Delta t = 100\,s$ (bottom).

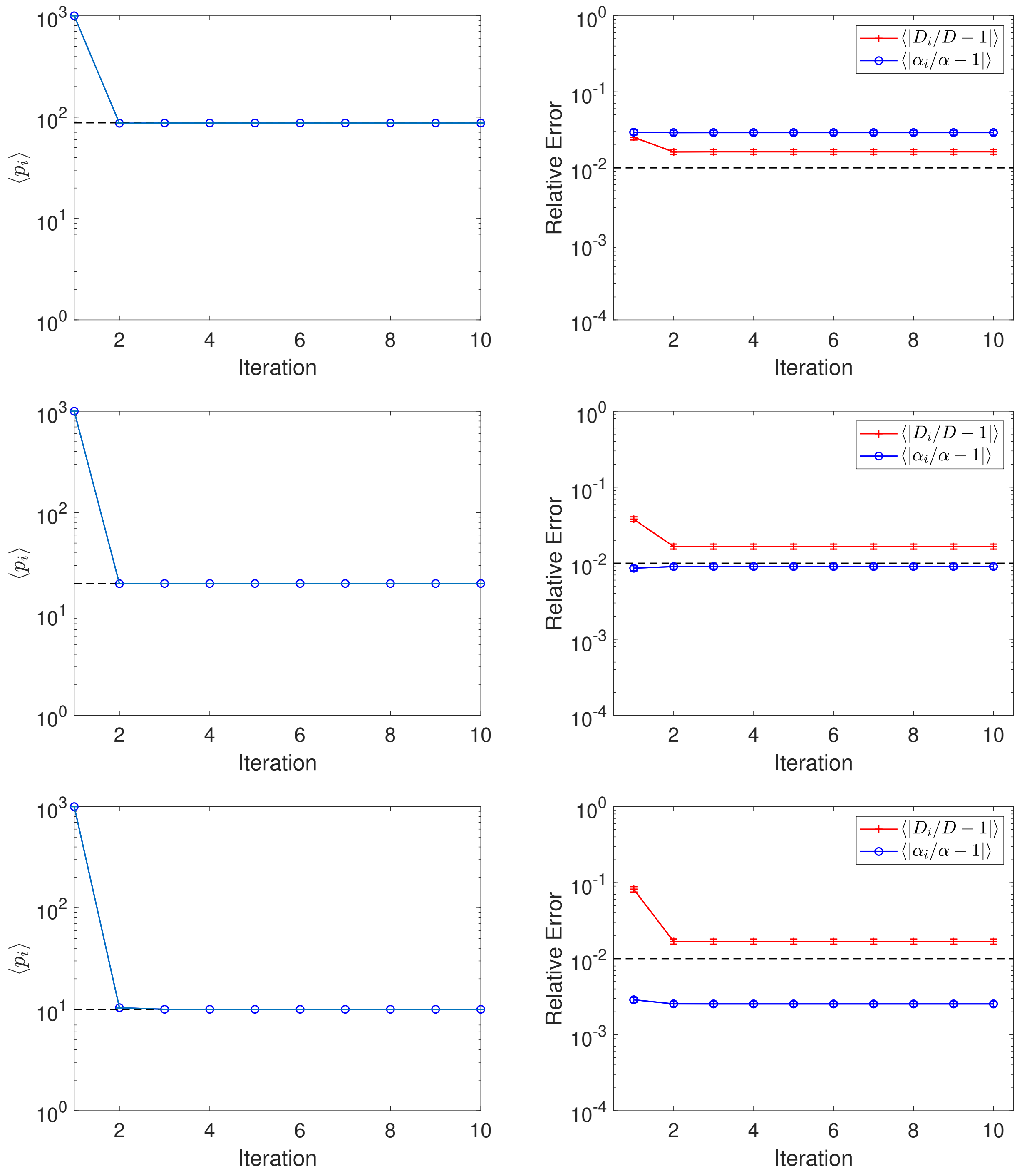

Figure 8: A plot of the value of $\langle p_i \rangle$ for each iteration with standard error bars (left plot in each row), along with a plot of the value of $\langle|D_i/D - 1|\rangle$ (+ red crosses) and $\langle|\alpha_i/\alpha - 1|\rangle$ (○ blue circles) for each iteration with standard error bars (right plot in each row). These experiments were for $D = 6\,\mu m^2/s$, $\alpha = 1\,\mu m/s$, $\eta = 2\,\mu m$, $N_S = 10$ and $N = 1000$. The dashed line in each left plot corresponds to $p_{opt} = 88$ (top), $p_{opt} = 20$ (middle), and $p_{opt} = 10$ (bottom), while the dashed line in each right plot corresponds with the value 10^{-2}, indicating a 1% error. These correspond with $\Delta t = 1\,s$ (top), $\Delta t = 10\,s$ (middle) and $\Delta t = 100\,s$ (bottom).

4.4 Additional single particle experiments for p_{opt}

We now test the iterative algorithm in the single particle case for the same parameters as in Section 4.2. First we look at $D = 2\,\mu m^2/s$, $\alpha = 7\,\mu m/s$, $\eta = 2\,\mu m$, $N_S = 1$ and $N = 1000$, for $\Delta t = 1\,s$, $\Delta t = 10\,s$ and $\Delta t = 100\,s$. The results are shown in Figure 9. We see that the results have similar dynamics to those from Figure 4 in the main paper. In all three cases, the value of p_i converges close to p_{opt}, the value of $\langle|\alpha_i/\alpha - 1|\rangle$ only decreases significantly when Δt is increased and the value of $\langle|D_i/D-1|\rangle$ is significantly improved when fit with p_{opt} points rather than all the points. Notice, however, for corresponding Δt values, that the final value of $\langle|\alpha_i/\alpha - 1|\rangle$ are always smaller, while the final value of $\langle|D_i/D - 1|\rangle$ are always larger, than those given in the main paper. This is expected as the larger value of α will cause the MSD to increase quicker for larger times, and so a smaller value of Δt would be required for better inference of D.

The case where $D = 6\,\mu m^2/s$, $\alpha = 1\,\mu m/s$, $\eta = 2\,\mu m$, $N_S = 1$ and $N = 1000$, for $\Delta t = 1\,s$, $\Delta t = 10\,s$ and $\Delta t = 100\,s$ was also tested and is shown in Figure 10. Again, the results show the same dynamics as those in the main paper. This time, however, we see that the final value of $\langle|\alpha_i/\alpha - 1|\rangle$ are always larger for corresponding values of Δt. Again, this is is expected as the larger value of D will in turn require a larger value of Δt to optimise the inference of α.

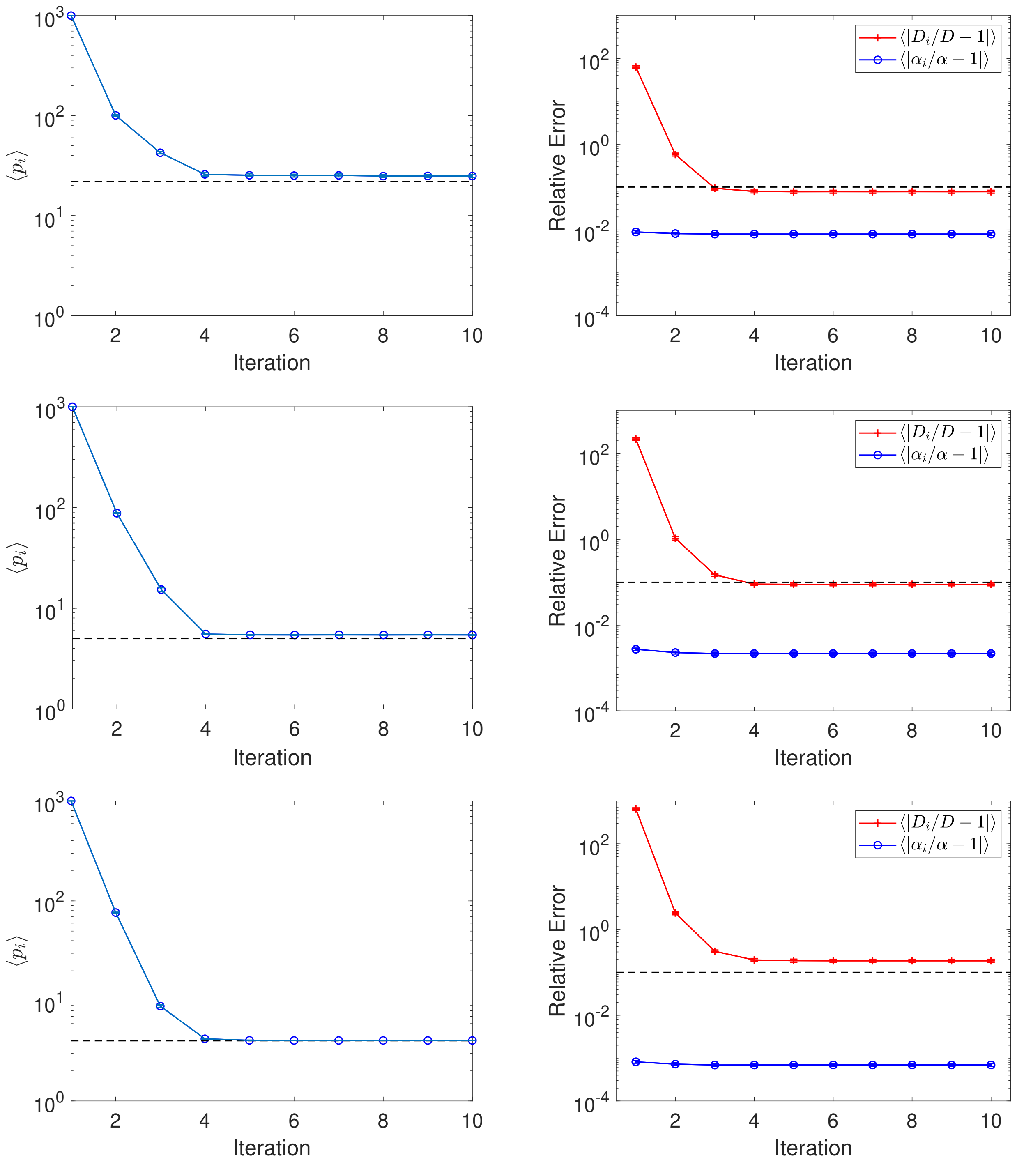

Figure 9: A plot of the value of $\langle p_i \rangle$ for each iteration with standard error bars (left plot in each row), along with a plot of the value of $\langle |D_i/D - 1| \rangle$ (+ red crosses) and $\langle |\alpha_i/\alpha - 1| \rangle$ (○ blue circles) for each iteration with standard error bars (right plot in each row). These experiments were for $D = 2\,\mu m^2/s$, $\alpha = 7\,\mu m/s$, $\eta = 2\,\mu m$, $N_S = 1$ and $N = 1000$. The dashed line in each left plot corresponds to $p_{opt} = 50$ (top), $p_{opt} = 16$ (middle), and $p_{opt} = 7$ (bottom), while the dashed line in each right plot corresponds with the value 10^{-1}, indicating a 10% error. These correspond with $\Delta t = 1\,s$ (top), $\Delta t = 10\,s$ (middle) and $\Delta t = 100\,s$ (bottom).

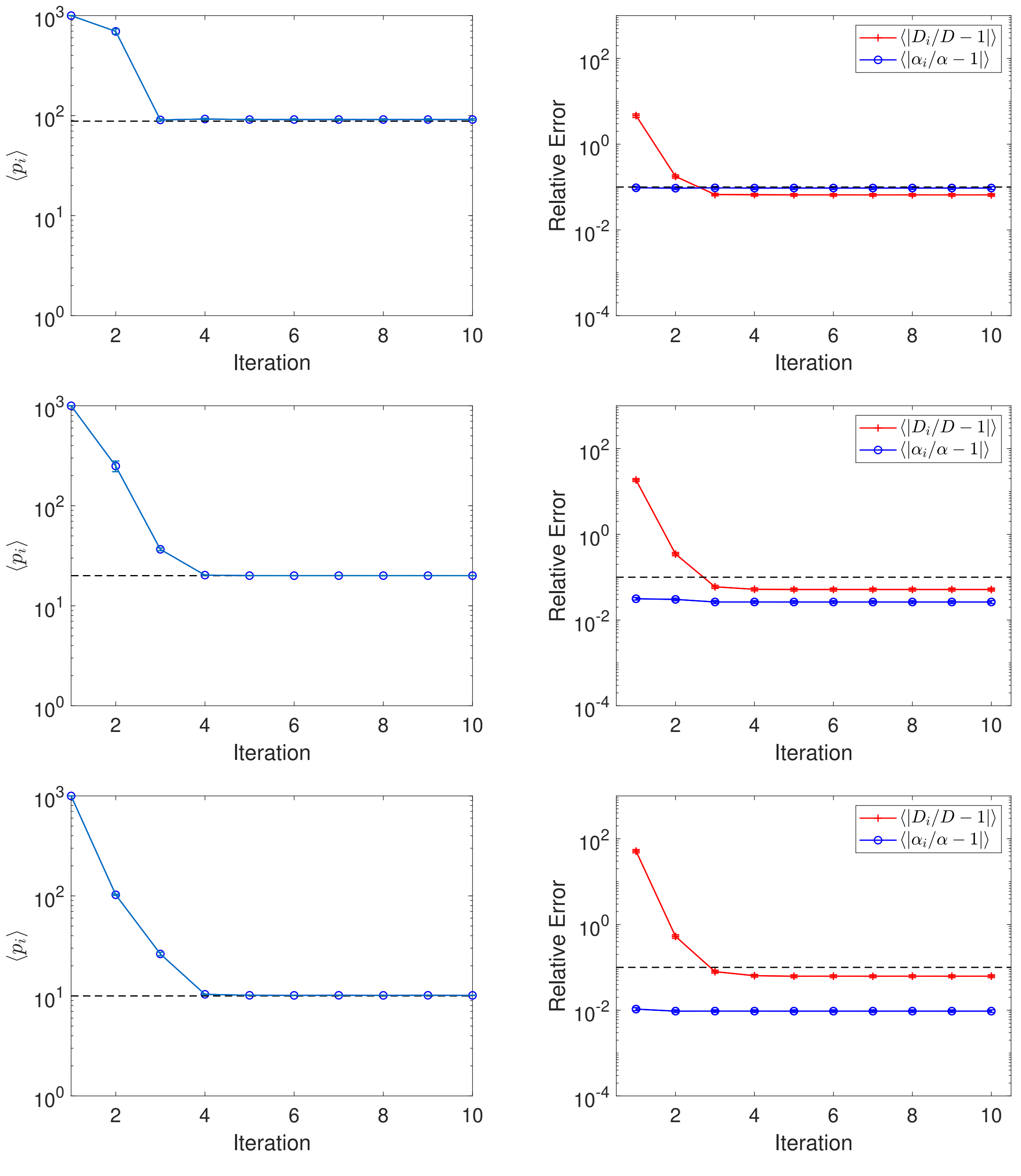

Figure 10: A plot of the value of $\langle p_i \rangle$ for each iteration with standard error bars (left plot in each row), along with a plot of the value of $\langle |D_i/D-1| \rangle$ (+ red crosses) and $\langle |\alpha_i/\alpha-1| \rangle$ (○ blue circles) for each iteration with standard error bars (right plot in each row). These experiments were for $D = 6\,\mu m^2/s$, $\alpha = 1\,\mu m/s$, $\eta = 2\,\mu m$, $N_S = 1$ and $N = 1000$. The dashed line in each left plot corresponds to $p_{opt} = 50$ (top), $p_{opt} = 16$ (middle), and $p_{opt} = 7$ (bottom), while the dashed line in each right plot corresponds with the value 10^{-1}, indicating a 10% error. These correspond with $\Delta t = 1\,s$ (top), $\Delta t = 10\,s$ (middle) and $\Delta t = 100\,s$ (bottom).

5 Further measurement time interval experiments

5.1 Existence of T_{opt} for different parameter values

As before, to test the robustness of our calculations, we now test the existence of an optimal measurement time interval with $N_S = 100$ and $N = 10$. Figure 11 shows the value of $\sigma_b/b + \sigma_c/c$, again with a comparison between theoretical expressions and simulations, for $\eta = 0.5\,\mu m$, $2\,\mu m$ and $8\,\mu m$. We see good agreement between the theory and simulations, particularly that both have their minimum's at the same times. We also notice that the optimal measurement time intervals have decreased to $T_{opt} \approx 189\,s$, $212\,s$ and $445\,s$. We have observed in general that as the value of N is increased the optimal measurement time interval T_{opt} also increases.

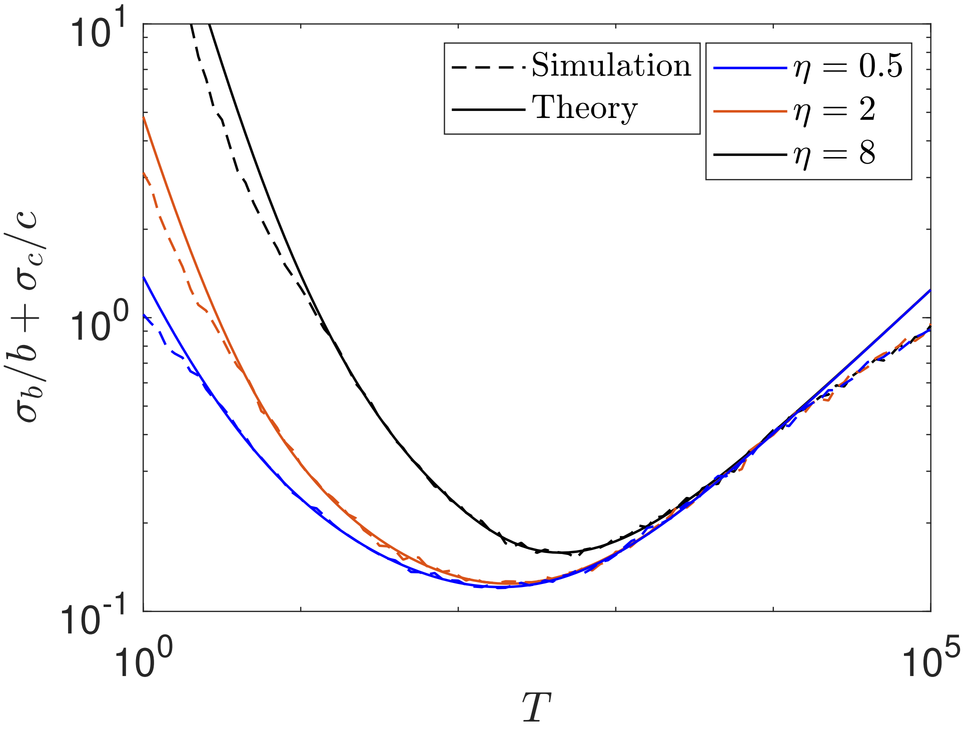

Figure 11: A plot of the theoretical value of $\sigma_b/b + \sigma_c/c$ (—— solid lines) and its empirically estimated value using 1000 samples (- - - dashed lines) against T for $\eta = 0.5\,\mu m$, $2\,\mu m$ and $8\,\mu m$. These experiments were for $D = 2\,\mu m^2/s$, $\alpha = 1\,\mu m/s$, $N_S = 100$ and $N = 10$. For $\eta = 0.5\,\mu m$, $2\,\mu m$ and $8\,\mu m$, the optimal measurement time intervals are $T_{opt} \approx 189\,s$, $212\,s$ and $445\,s$, respectively.

Again, we also look for the existence of an optimal measurement time interval which minimises the uncertainty $\sigma_a/a + \sigma_b/b + \sigma_c/c$ in order to infer the diffusion coefficient D, the drift magnitude α and the observational noise η. The results are plotted in Figure 12. We can see good agreement between the theoretical and simulated uncertainties, which for $\eta = 0.5\,\mu m$, $2\,\mu m$ and $8\,\mu m$, have corresponding optimal measurement time intervals $T_{opt} \approx 32\,s$, $178\,s$ and $708\,s$, respectively.

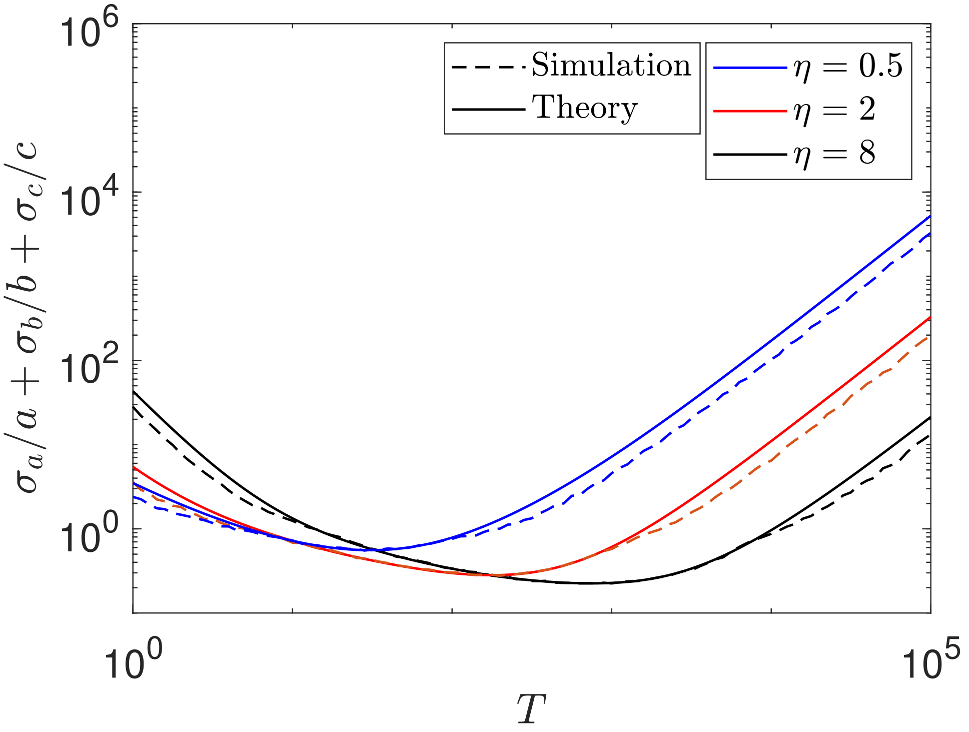

Figure 12: A plot of the theoretical value of $\sigma_a/a+\sigma_b/b+\sigma_c/c$ (—— solid lines) and its empirically estimated value using 1000 samples (- - - dashed lines) against T for $\eta = 0.5\,\mu m$, $2\,\mu m$ and $8\,\mu m$. These experiments were for $D = 2\,\mu m^2/s$, $\alpha = 1\,\mu m/s$, $N_S = 100$ and $N = 10$. For $\eta = 0.5\,\mu m$, $2\,\mu m$ and $8\,\mu m$, the optimal measurement time intervals are $T_{opt} \approx 32\,s$, $178\,s$ and $708\,s$, respectively.

5.2 MATLAB routine to calculate optimal time measurement interval

```
%%%%%%%%%%%%%%%%%%%%%%%%%%%%%%%%%%%%%%%%%%%%%%%%%%%%%%%%%%%%%%%%%%%%%%%%%%%
%                                                                         %
% Code to calculate the optimal measurement time interval for the        %
% overlapping time—averaged mean—square displacement (MSD).              %
%                                                                         %
%%%%%%%%%%%%%%%%%%%%%%%%%%%%%%%%%%%%%%%%%%%%%%%%%%%%%%%%%%%%%%%%%%%%%%%%%%%


%
% Input:    D — Estimate of the diffusion coefficient.
%          al — Estimate of the drift magnitude.
%         eta — Estimate of the observational noise.
%          N  — The number of MSD points.
%
% Output:  Topt — An estimate of the optimal measurement time interval.
%
%
% Notes —  This code works for the overlapping time—averaged MSD
%          calculated both from a single particle and an ensemble of
```

```
%           particles as they have the same optimal measurement time
%           interval.
%

function [Topt] = optimal_over(D,al,eta,N)

NT=251;
Topt_init=100;

for i=1:NT
    T=Topt_init*(1.03)^(i-(NT+2)/2);
    time(i)=T;
    dt=T/N;
    alp=4*D*dt;
    ep=4*eta^2;

%
% Calculate the variance of the MSD
%

    for n=1:N
        K=N+1-n;
        if n<=K
            varmsd(n)=(n*(4*n^2*K+2*K-n^3+n)/(6*K^2))*(4*D*dt)^2+...
                8*al^2*D*(dt)^3*(n^3*(3*K*n+1-n^2)/(3*K^2));
            varmsd(n)=varmsd(n)+((8*eta^2)/(K^2))*((K-n)*(eta^2-...
                al^2*(n*dt)^2)+K*(al^2*(n*dt)^2+4*D*n*dt+2*eta^2));
        else
            varmsd(n)=((6*n^2*K-4*n*K^2+4*n+K^3-K)/(6*K))*((4*D*dt)^2)+...
                8*al^2*D*(dt)^3*(n^2*(3*n*K+1-K^2))/(3*K);
            varmsd(n)=varmsd(n)+8*eta^2*(al^2*(n*dt)^2+4*D*n*dt+2*eta^2)/K;
        end
    end

%
% Calculate the covariance of the MSD
%

    for n=1:N
        for m=1:N
            K=N+1-n;
            P=N+1-m;
            if m+n<=N+1
                covarmsd(n,m)=((alp^2*n)/(6*K*P))*(-n^3-2*P*n^2+(1-...
                    6*m^2+(6*N+6)*m)*n+2*P)+2*ep*n*alp/K+...
                    ((8*al^2*dt^3*m*n^2*D)/(3*K*P))*(-n^2+3*(-m^2+...
                    (N+1)*m+1/3))+((-n+2*P)*ep^2)/(2*K*P)+...
```

```
                (2*al^2*dt^2*m*n^2*ep)/(K*P);
            else
                covarmsd(n,m)=((alp^2)/(6*K))*(-m^3+(3+3*N-4*n)*m^2+...
                    ((8+8*N)*n-2-3*N^2-6*N)*m-6*n^3+(6+6*N)*n^2-...
                    (4*N^2+8*N)*n+N*(N+2)*(N+1))-...
                    ((8*al^2*dt^3*m*n*D)/(3*K))*(m^2-2*(N+1)*m+...
                    (3*n^2-3*(N+1)*n+N^2+2*N))+...
                    (2*(al^2*dt^2*m*n+n*alp)*ep+ep^2/2)/K;
            end
        end
    end

    for n=1:N
        for m=1:n
            covarmsd(n,m)=0;
        end
    end

%
% Calculate the variance of the regression coefficients
%

    s0=0;s1=0;s2=0;s3=0;s4=0;
    for n=1:N
        tn=(n*T/N);
        vari=varmsd(n);
        s0=s0+1/vari;
        s1=s1+tn/vari;
        s2=s2+tn^2/vari;
        s3=s3+tn^3/vari;
        s4=s4+tn^4/vari;
    end

    del=s0*s2*s4-s0*(s3)^2-...
        (s1)^2*s4+2*s1*s2*s3-(s2)^3;

    var_a(i)=0;
    var_b(i)=0;
    var_c(i)=0;

    for n=1:N
        tn=(n*T/N);
        vari=varmsd(n);
        da(n)=(s2*s4-s3^2-s1*s4*tn+...
            s1*s3*tn^2+s2*s3*tn-s2^2*tn^2)/...
            (del*vari);
        db(n)=(s0*s4*tn-s0*s3*tn^2-s1*s4+...
```

```
            s2*s3+s1*s2*tn^2-s2^2*tn)/...
            (del*vari);
        dc(n)=(s0*s2*tn^2-s0*s3*tn-...
            s1^2*tn^2+s1*s2*tn+s1*s3-s2^2)/...
            (del*vari);
        var_a(i)=var_a(i)+vari*da(n)^2;
        var_b(i)=var_b(i)+vari*db(n)^2;
        var_c(i)=var_c(i)+vari*dc(n)^2;
        for m=1:n-1
            var_a(i)=var_a(i)+2*covarmsd(m,n)*da(n)*da(m);
            var_b(i)=var_b(i)+2*covarmsd(m,n)*db(n)*db(m);
            var_c(i)=var_c(i)+2*covarmsd(m,n)*dc(n)*dc(m);
        end
    end
end

%
% Calculate the optimal measurement time interval
%

[~,I]=min(sqrt(var_b)/(4*D)+sqrt(var_c)/(al^2));
Topt=time(I);

end
```

5.3 Additional iterative algorithm experiments for T_{opt}

The iterative algorithm was tested for other values of the adaptation parameter ψ, as well as more values of D and α, to test the robustness of the algorithm. First, we look at testing the iterative algorithm, but changing the adaptation parameter (Algorithm 2, Step 10 in the main paper) to $\psi = 0.5$ and $\psi = 0.2$. Both of these experiments were for $D = 2\,\mu m^2/s$, $\alpha = 1\,\mu m/s$ and $\eta = 2\,\mu m$ with the two initial measurement time intervals, $T_0 = 10^7\,s$ and $T_0 = 10^{-3}\,s$. The results are shown in Figures 13 and 14. The reduction of the adaptation parameter does not appear to have affected the inference in either case. The value of $\langle T_i \rangle$ still converges to a time close to T_{opt} and the final value of both $\langle |D_i/D - 1| \rangle$ and $\langle |\alpha_i/\alpha - 1| \rangle$ are under 10% in both cases.

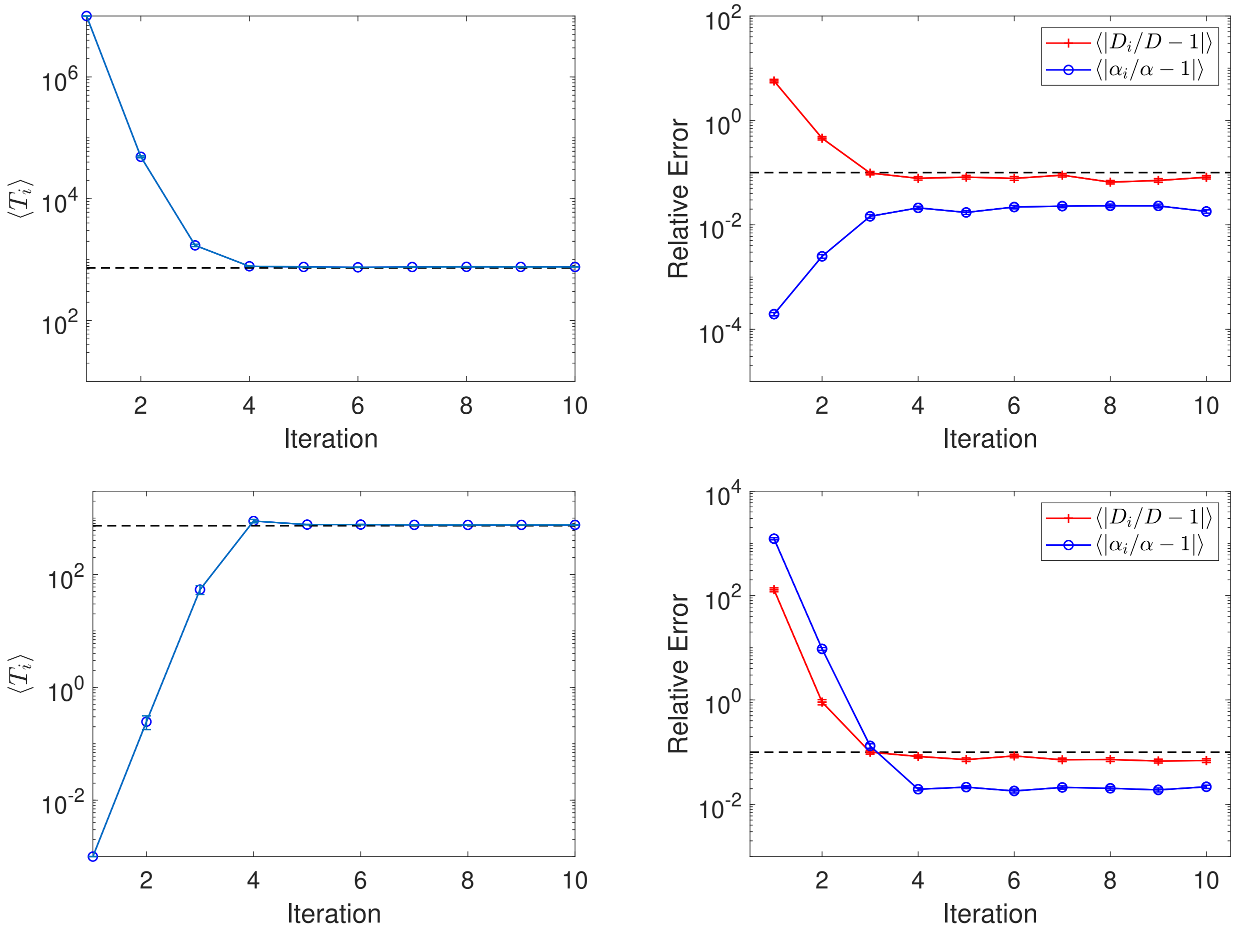

Figure 13: A plot of the value of $\langle T_i \rangle$ for each iteration with standard error bars (left in each), along with a plot of the value of $\langle |D_i/D - 1| \rangle$ (+ red crosses) and $\langle |\alpha_i/\alpha - 1| \rangle$ (○ blue circles) for each iteration with standard error bars (right in each). These experiments were for $D = 2\,\mu m^2/s$, $\alpha = 1\,\mu m/s$, $\eta = 2\,\mu m$, $N_S = 10$, $N = 100$ and $\psi = 0.5$, with a starting time of $T_0 = 10^7\,s$ (top) and $T_0 = 10^{-3}\,s$ (bottom). The dashed line in each left plot corresponds to $T_{opt} \approx 780\,s$, while the dashed line in each right plot corresponds with the value 10^{-1}, indicating a 10% error.

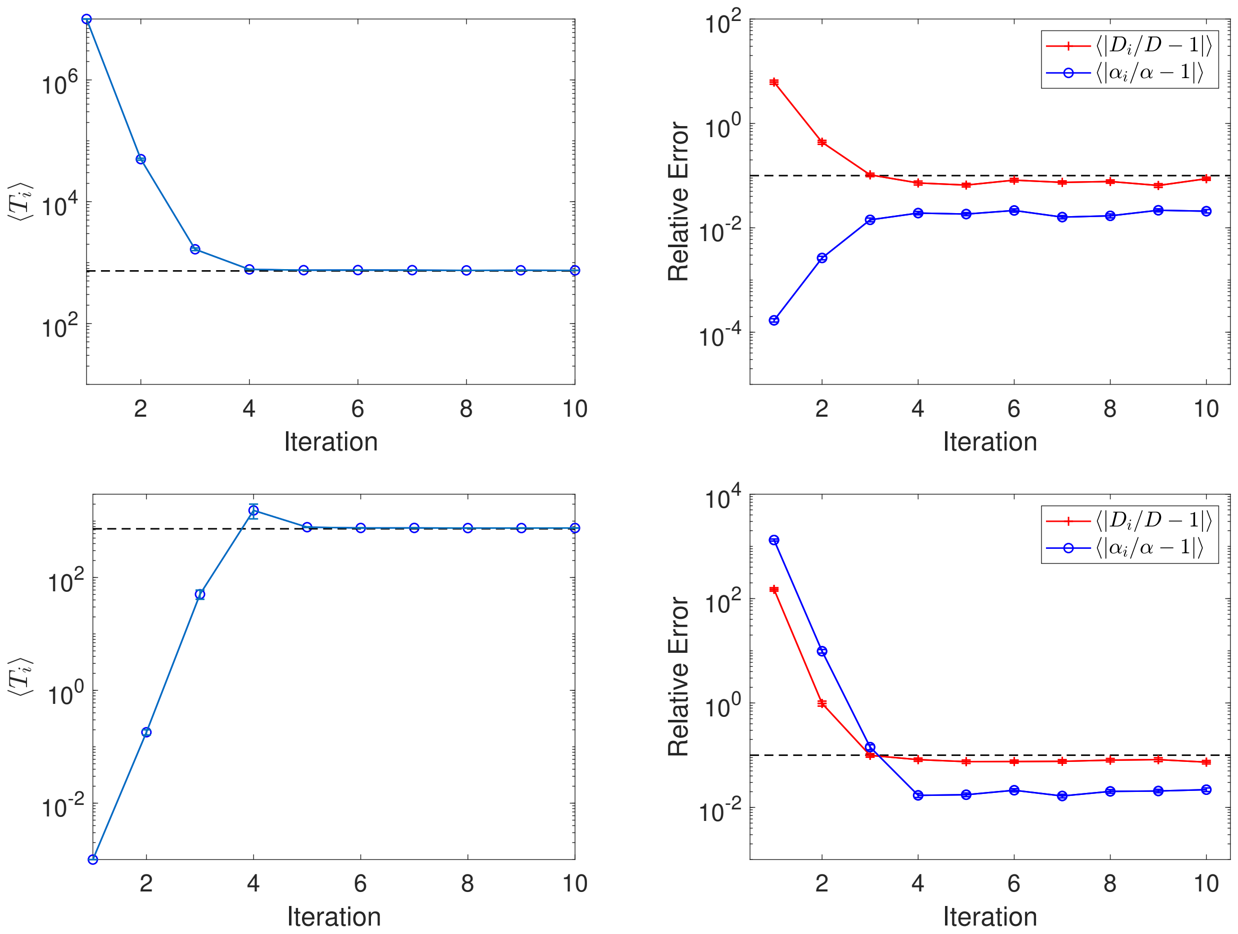

Figure 14: A plot of the value of $\langle T_i \rangle$ for each iteration with standard error bars (left in each), along with a plot of the value of $\langle |D_i/D-1| \rangle$ (+ red crosses) and $\langle |\alpha_i/\alpha-1| \rangle$ (○ blue circles) for each iteration with standard error bars (right in each). These experiments were for $D = 2\,\mu m^2/s$, $\alpha = 1\,\mu m/s$, $\eta = 2\,\mu m$, $N_S = 10$, $N = 100$ and $\psi = 0.2$, with a starting time of $T_0 = 10^7\,s$ (top) and $T_0 = 10^{-3}\,s$ (bottom). The dashed line in each left plot corresponds to $T_{opt} \approx 780\,s$, while the dashed line in each right plot corresponds with the value 10^{-1}, indicating a 10% error.

We now test adapting the values of D and α. The first experiment is for $D = 2\,\mu m^2/s$, $\alpha = 7\,\mu m/s$ and $\eta = 2\,\mu m$; for these parameters $T_{opt} \approx 32\,s$. The second experiment is for $D = 6\,\mu m^2/s$, $\alpha = 1\,\mu m/s$ and $\eta = 2\,\mu m$; for these parameters $T_{opt} \approx 2195\,s$. The value of the adaptation parameter for both experiments were set back to $\psi = 0.8$. The results are plotted in Figure 15 and 16, respectively. As before, the results are very similar to what has been seen before. As both cases converge to T_{opt} the relative errors are reduced, particularly for a smaller value of the measurement time interval where the reduction is more significant.

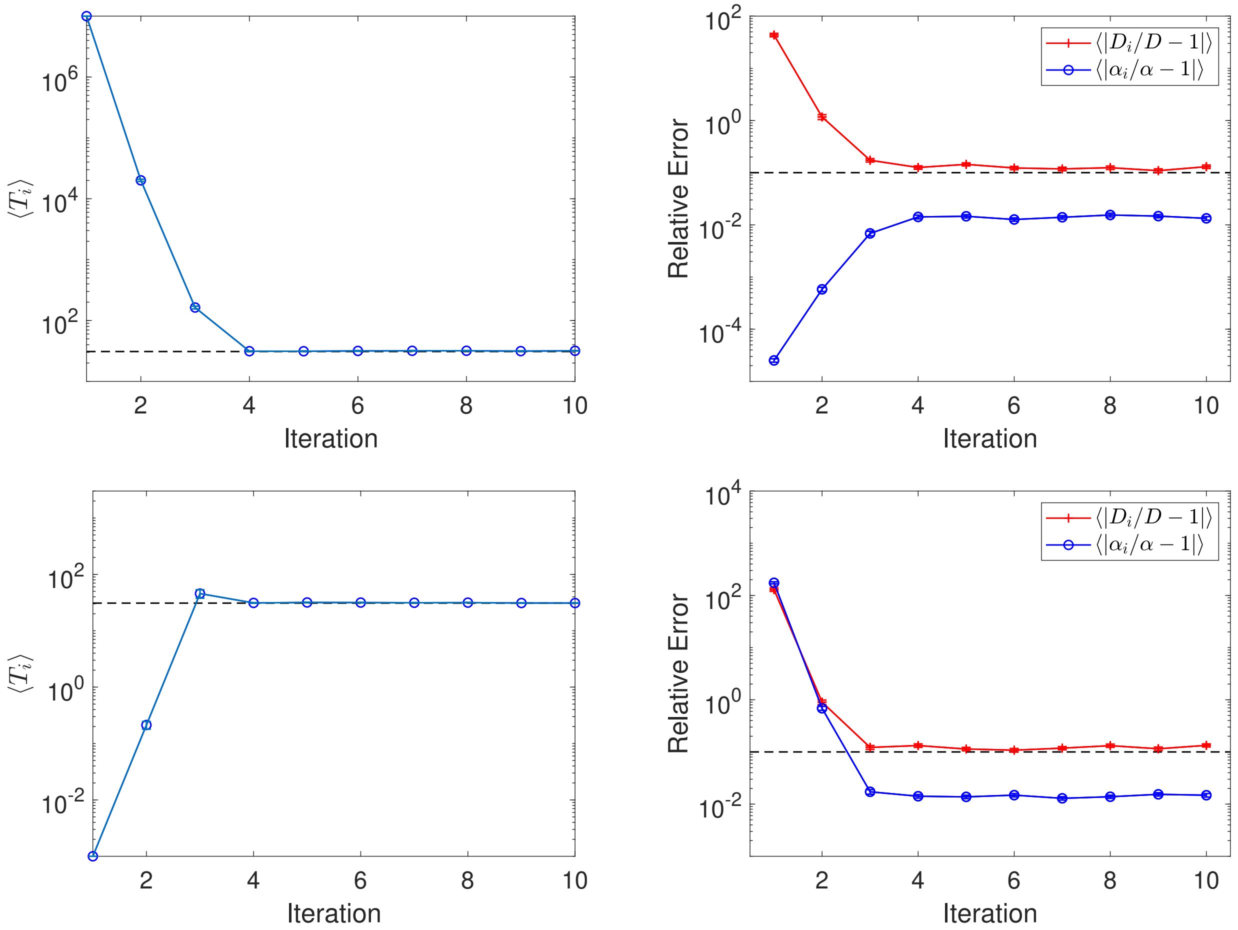

Figure 15: A plot of the value of $\langle T_i \rangle$ for each iteration with standard error bars (left in each), along with a plot of the value of $\langle |D_i/D - 1| \rangle$ (+ red crosses) and $\langle |\alpha_i/\alpha - 1| \rangle$ (○ blue circles) for each iteration with standard error bars (right in each). These experiments were for $D = 2\,\mu m^2/s$, $\alpha = 7\,\mu m/s$, $\eta = 2\,\mu m$, $N_S = 10$ and $N = 100$, with a starting time of $T_0 = 10^7\,s$ (top) and $T_0 = 10^{-3}\,s$ (bottom). The dashed line in each left plot corresponds to $T_{opt} \approx 32\,s$, while the dashed line in each right plot corresponds with the value 10^{-1}, indicating a 10% error.

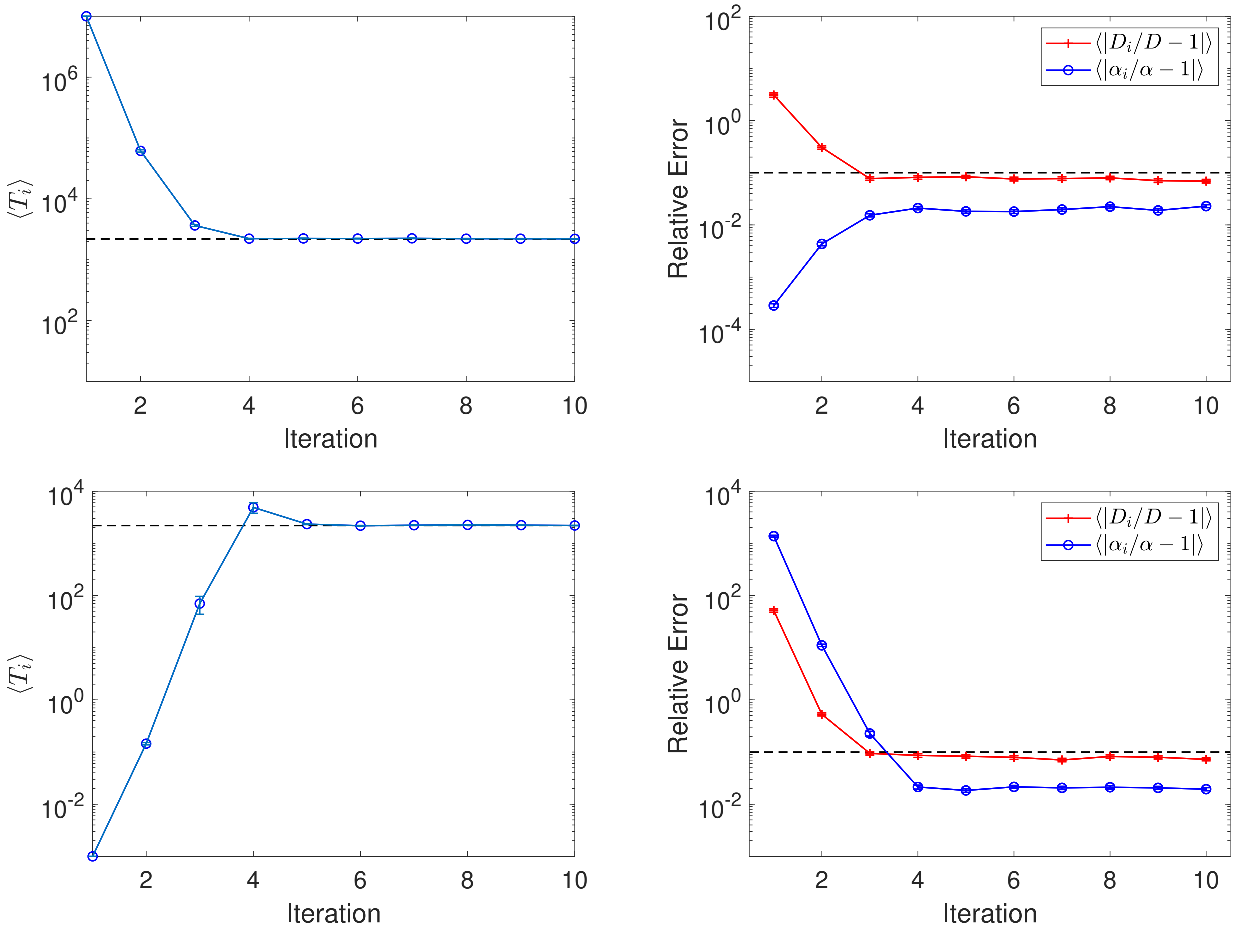

Figure 16: A plot of the value of $\langle T_i \rangle$ for each iteration with standard error bars (left in each), along with a plot of the value of $\langle |D_i/D - 1| \rangle$ (+ red crosses) and $\langle |\alpha_i/\alpha - 1| \rangle$ (○ blue circles) for each iteration with standard error bars (right in each). These experiments were for $D = 6\,\mu m^2/s$, $\alpha = 1\,\mu m/s$, $\eta = 2\,\mu m$, $N_S = 10$ and $N = 100$, with a starting time of $T_0 = 10^7\,s$ (top) and $T_0 = 10^{-3}\,s$ (bottom). The dashed line in each left plot corresponds to $T_{opt} \approx 2195\,s$, while the dashed line in each right plot corresponds with the value 10^{-1}, indicating a 10% error.

5.4 Additional single particle experiments for T_{opt}

We look at using the iterative algorithm in the single particle case for different values of D and α. First we test for $D = 2\,\mu m^2/s$, $\alpha = 7\,\mu m/s$, $\eta = 2\,\mu m$, $N_S = 1$ and $N = 100$, again for both $T_0 = 10^7\,s$ and $T_0 = 10^{-3}\,s$. For these parameter values, we have that $T_{opt} \approx 32\,s$. The results are given in Figure 17. The algorithm takes a few more iterations to converge compared with the ensemble of particles case. However, we still see convergence in a small number of iterations, especially for a smaller measurement time interval.

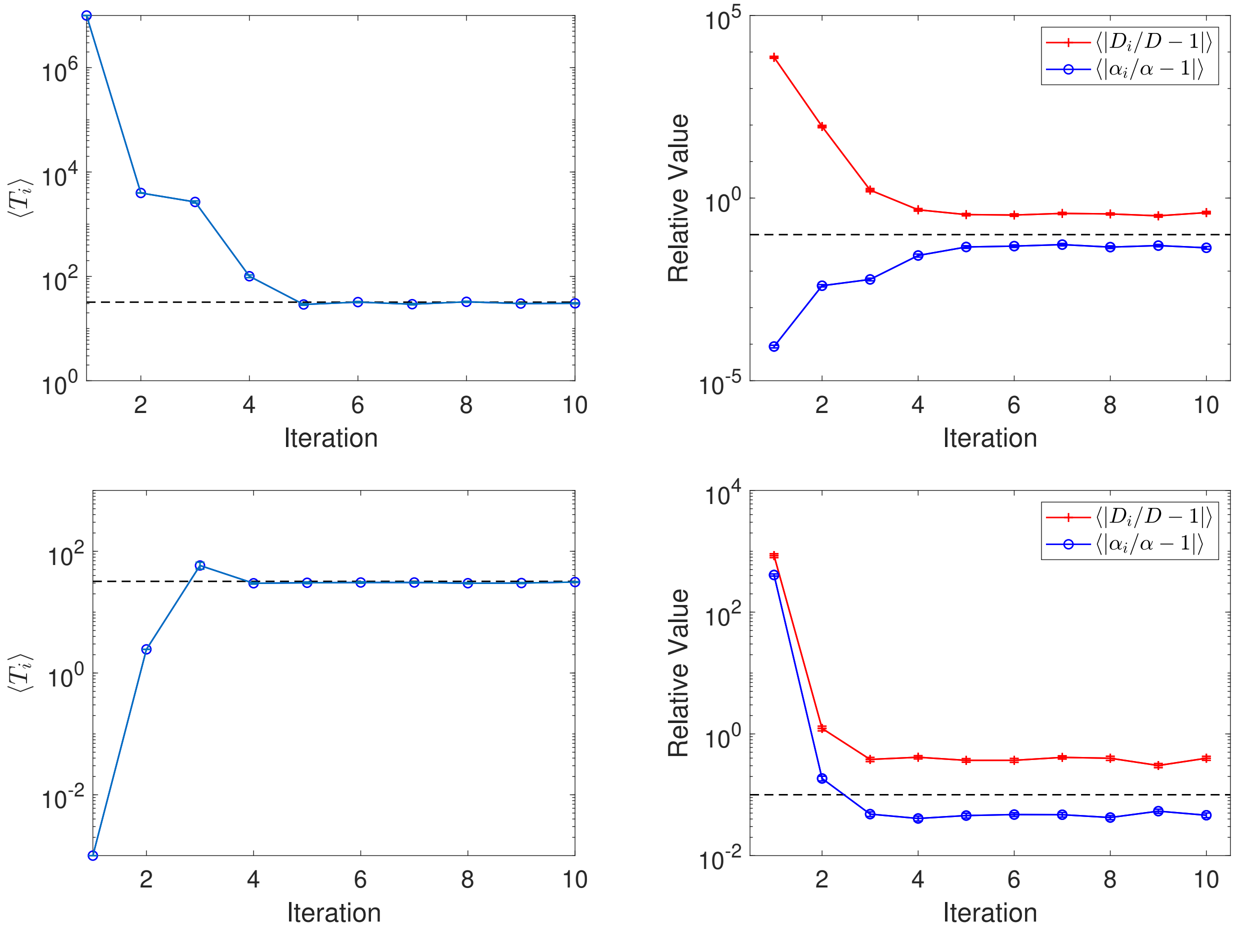

Figure 17: A plot of the value of $\langle T_i \rangle$ for each iteration with standard error bars (left in each), along with a plot of the value of $\langle |D_i/D - 1| \rangle$ (+ red crosses) and $\langle |\alpha_i/\alpha - 1| \rangle$ (○ blue circles) for each iteration with standard error bars (right in each). These experiments were for $D = 2\,\mu m^2/s$, $\alpha = 7\,\mu m/s$ and $\eta = 2\,\mu m$, $N_S = 1$ and $N = 100$, with a starting time of $T_0 = 10^7\,s$ (top) and $T_0 = 10^{-3}\,s$ (bottom). The dashed line in the left plots correspond to $T_{opt} \approx 32\,s$, while the dashed line in the right plots correspond with the value 10^{-1}, indicating a 10% error.

The final parameter values tested are $D = 6\,\mu m^2/s$, $\alpha = 1\,\mu m/s$, $\eta = 2\,\mu m$, $N_S = 1$ and $N = 100$, again for both $T_0 = 10^7$ and $T_0 = 10^{-3}$. For these parameter values, we have that $T_{opt} \approx 2195\,s$. The results are given in Figure 18. The algorithm continues to converge in a small number of iterations and reduce the errors significantly in both cases.

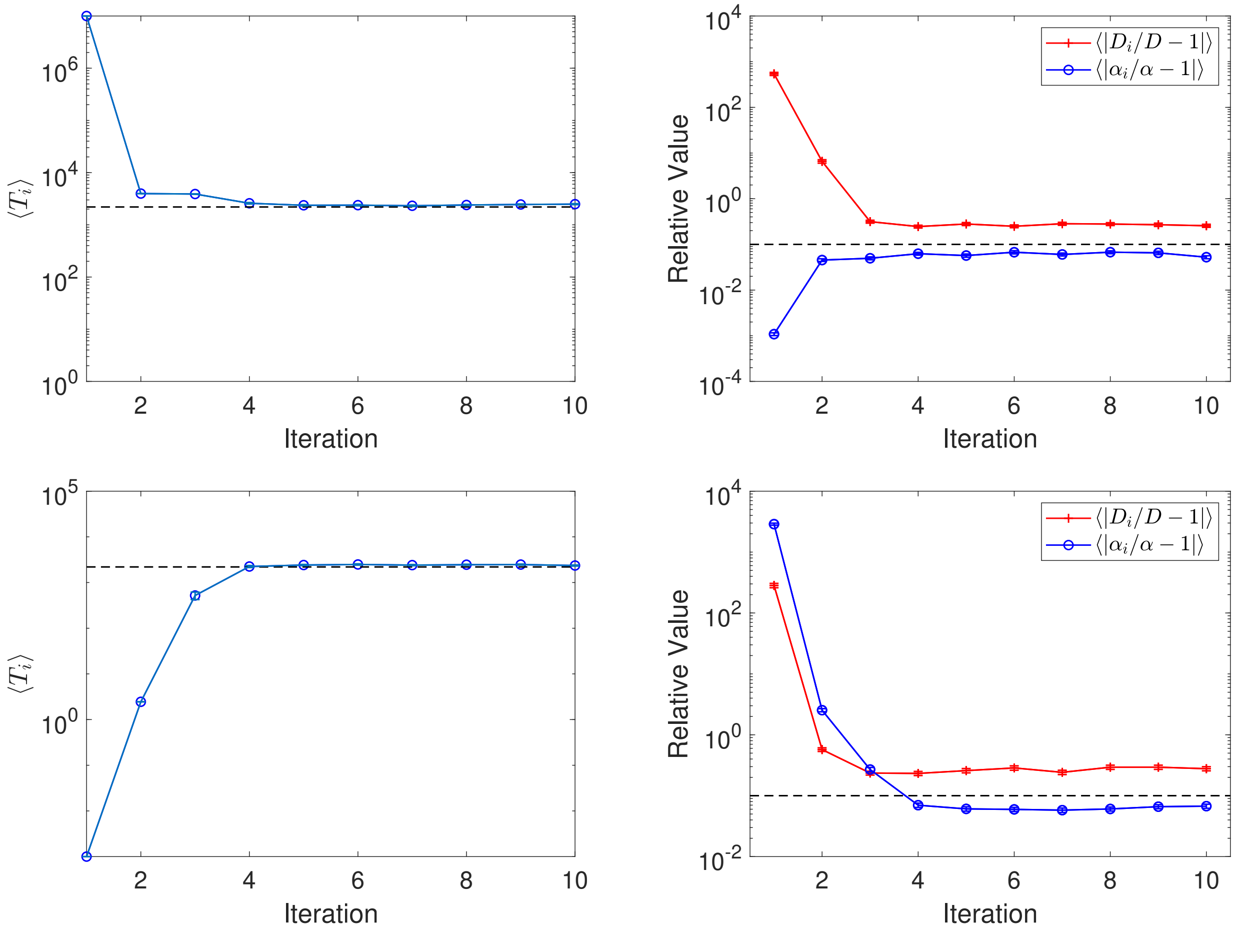

Figure 18: A plot of the value of $\langle T_i \rangle$ for each iteration with standard error bars (left in each), along with a plot of the value of $\langle|D_i/D-1|\rangle$ (+ red crosses) and $\langle|\alpha_i/\alpha-1|\rangle$ (○ blue circles) for each iteration with standard error bars (right in each). These experiments were for $D = 6\,\mu m^2/s$, $\alpha = 1\,\mu m/s$ and $\eta = 2\,\mu m$, $N_S = 1$ and $N = 100$, with a starting time of $T_0 = 10^7\,s$ (top) and $T_0 = 10^{-3}\,s$ (bottom). The dashed line in the left plots correspond to $T_{opt} \approx 2195\,s$, while the dashed line in the right plots correspond with the value 10^{-1}, indicating a 10% error.

6 Motion blur

It is of interest what effect motion blur has on the MSD. To answer this question we follow the approach of Goulian and Simon [?], who considered the effect of full-frame motion blur on the MSD of a purely diffusive motion. We assume that the particle position in the nth image, $\boldsymbol{x}(t)$ with $t = n\Delta t$, is taken to be the combination of the average of the true position of the particle $\tilde{\boldsymbol{x}}(t)$ over the time frame and the addition of static Gaussian measurement noise. If the time interval Δt is divided by M smaller microsteps of size $\delta t = \Delta t/M$, then we assume that the measured position

$$\boldsymbol{x}(t) = \frac{1}{M+1}\sum_{m=0}^{M}\tilde{\boldsymbol{x}}(t - m\delta t) + \boldsymbol{\eta}_t \equiv \bar{\boldsymbol{x}}(t) + \boldsymbol{\eta}_t.$$

We then have for $t' \geq t + M\delta t$

$$\begin{aligned}\mathbb{E}((\boldsymbol{x}(t)-\boldsymbol{x}(t'))^2) &= \mathbb{E}((\bar{\boldsymbol{x}}(t)+\boldsymbol{\eta}_t-\bar{\boldsymbol{x}}(t')-\boldsymbol{\eta}_{t'})^2) \\ &= \mathbb{E}((\bar{\boldsymbol{x}}(t)-\bar{\boldsymbol{x}}(t'))^2)+2\mathbb{E}((\boldsymbol{\eta}_t-\boldsymbol{\eta}_{t'})(\bar{\boldsymbol{x}}(t)-\bar{\boldsymbol{x}}(t')))+\mathbb{E}((\boldsymbol{\eta}_t-\boldsymbol{\eta}_{t'})^2) \\ &= \mathbb{E}((\bar{\boldsymbol{x}}(t)-\bar{\boldsymbol{x}}(t'))^2)+4\eta^2. \end{aligned} \tag{13}$$

Furthermore, we have

$$\begin{aligned}&\mathbb{E}((\bar{\boldsymbol{x}}(t)-\bar{\boldsymbol{x}}(t'))^2) \\ &= \frac{1}{(M+1)^2}\sum_{m_1,m_2=0}^{M}\mathbb{E}([\tilde{\boldsymbol{x}}(t-m_1\delta t)-\tilde{\boldsymbol{x}}(t'-m_1\delta t)][\tilde{\boldsymbol{x}}(t-m_2\delta t)-\tilde{\boldsymbol{x}}(t'-m_2\delta t)] \\ &= \frac{1}{2(M+1)^2}\sum_{m_1,m_2=0}^{M}2\mathbb{E}([\tilde{\boldsymbol{x}}(t-m_1\delta t)-\tilde{\boldsymbol{x}}(t'-m_2\delta t)]) \\ &\quad -\mathbb{E}(\tilde{\boldsymbol{x}}(t-m_1\delta t)-\tilde{\boldsymbol{x}}(t-m_2\delta t))-\mathbb{E}(\tilde{\boldsymbol{x}}(t'-m_1\delta t)-\tilde{\boldsymbol{x}}(t'-m_2\delta t)) \\ &= \frac{1}{(M+1)^2}\sum_{m_1,m_2=0}^{M}\big[\alpha^2(t'-t-(m_2-m_1)\delta t)^2+4D(t'-t-(m_2-m_1)\delta t) \\ &\quad -(\alpha|m_2-m_1|\delta t)^2+4D|m_2-m_1|\delta t)\big] \\ &= \alpha^2(t'-t)^2+4D\left(t'-t-\frac{(M+1)^2-1}{3(M+1)}\delta t\right). \end{aligned} \tag{14}$$

For the MSD we take $t'-t=n\Delta t$, and using (13) and (14) and letting $M\to\infty$ we finally get

$$MSD(n\Delta t)=\alpha^2(n\Delta t)^2+4Dn\Delta t+4\left(\eta^2-\frac{D\Delta t}{3}\right). \tag{15}$$

It is clear that motion blur only effects the offset of the MSD curve for zero time lags. In particular, if the MSD is fitted by a quadratic polynomial then the quadratic term can be used to estimate the drift velocity and the linear term used to estimate the diffusion coefficient. Note that the same MSD expression given by (15) can be obtained using the general formula for the MSD subject to dynamic and static localization errors derived in Savin and Doyle [**?**].

The effect of motion blur and static measurement noise was simulated by first generating true particle trajectories using microsteps of size

$$\delta t=\frac{\Delta t}{M},$$

where Δt is the time between frames and M is the number of microsteps between frames. For the jth particle the displacement was updated as follows:

$$\tilde{\boldsymbol{x}}_{i+1}^{(j)}=\tilde{\boldsymbol{x}}_i^{(j)}+\boldsymbol{\alpha}\delta t+\sqrt{2D\delta t}\boldsymbol{\mathcal{N}}(0,1), \qquad i=1,\ldots,NM+1,$$

with $\tilde{\boldsymbol{x}}_1=0$. The measured displacement at $t=(n-1)\Delta t$ was obtained by averaging the true position of the particle over the previous M microsteps to simulate full-frame motion blur, and a Gaussian static measurement noise was then added so that

$$\boldsymbol{x}_n^{(j)}=\frac{1}{M+1}\sum_{i=(n-2)M+1}^{(n-1)M+1}\tilde{\boldsymbol{x}}_i^{(j)}+\boldsymbol{\eta}_n, \qquad n=2,\ldots,N+1,$$

and $\boldsymbol{x}_1^{(j)} = \boldsymbol{\eta}_1$.

The following results are for calculating the optimal number of fitting points. First, we look for the existence of an optimal number of fitting points which minimises the value of $\sigma_b/b+\sigma_c/c$. Figure 19 shows a comparison between the theoretical value of $\sigma_b/b+\sigma_c/c$ assuming no motion blur and its simulated value which includes motion blur. We see that for small Δt, corresponding to using all the MSD points in the fitting, motion blur does not seem to have an effect on the results. However, when Δt is increased, motion blur appears to effect the value of $\sigma_b/b+\sigma_c/c$. While the theoretical optimal number of fitting points is 8, the simulated optimal number of fitting points is 5.

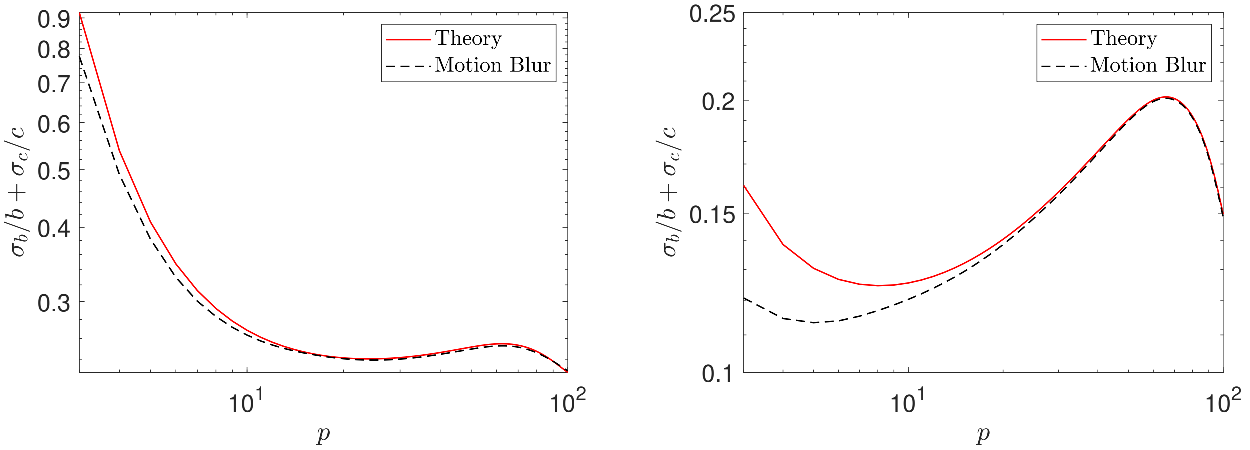

Figure 19: A plot of the theoretical value of $\sigma_b/b+\sigma_c/c$ assuming no motion blur (—— solid red line) and an empirically estimated value found using 1000 samples which include motion blur (- - - dashed black line) when fit with the first p MSD points for $D = 2\,\mu m^2/s$, $\alpha = 1\,\mu m/s$, $\eta = 2\,\mu m$, $N_S = 10$ and $N = 100$, for $\Delta t = 1\,s$ giving $T = 100\,s$ (left), and $\Delta t = 10\,s$ giving $T = 1000\,s$ (right). The optimal number of fitting points for the left plot are both 100, while for the right it is 8 (theory) and 5 (simulations).

Since it can be seen that motion blur has an effect on the value of p_{opt}, we look to see whether using our theoretical value for p_{opt} on data which includes motion blur leads to a change in results. To test this, we will use the iterative algorithm to see the effect of motion blur on the inference of D and α. For this, the simulated data will include motion blur but will be driven towards the theoretical p_{opt} which does not include motion blur (see section 4.2). In the absence of motion blur, the regression coefficient $c = 4\eta^2$. However, when motion blur is present, the regression coefficient becomes $c = 4\eta^2 - \frac{4}{3}D\Delta t$ and so the value of η will be inferred with this in mind. The results are shown in Figure 20. For this we have $D = 2\,\mu m^2/s$, $\alpha = 1\,\mu m/s$, $\eta = 2\,\mu m$, $N_S = 10$ and $N = 1000$ for $\Delta t = 1\,s$, $\Delta t = 10\,s$ and $\Delta t = 100\,s$. Note that these plots are comparable with Figure 3 from the main paper. Even though we are driving p_i to the theoretical optimal number of fitting points which does not include motion blur, we do not see a significant difference in the inference of D and α when motion blur is included. This suggests that our theory for the optimal number of fitting points can still be used when motion blur is present.

To further test the effects of motion blur, we also tested the iterative algorithm for a single particle. The parameters values were chosen to be the same as those above but for $N_S = 1$. The results are shown in Figure 21. Note that these plots are comparable with Figure 4 from

the main paper. Again, we do not see a significant difference with the inclusion of motion blur, further suggesting that our theoretical p_{opt} value works when motion blur is present.

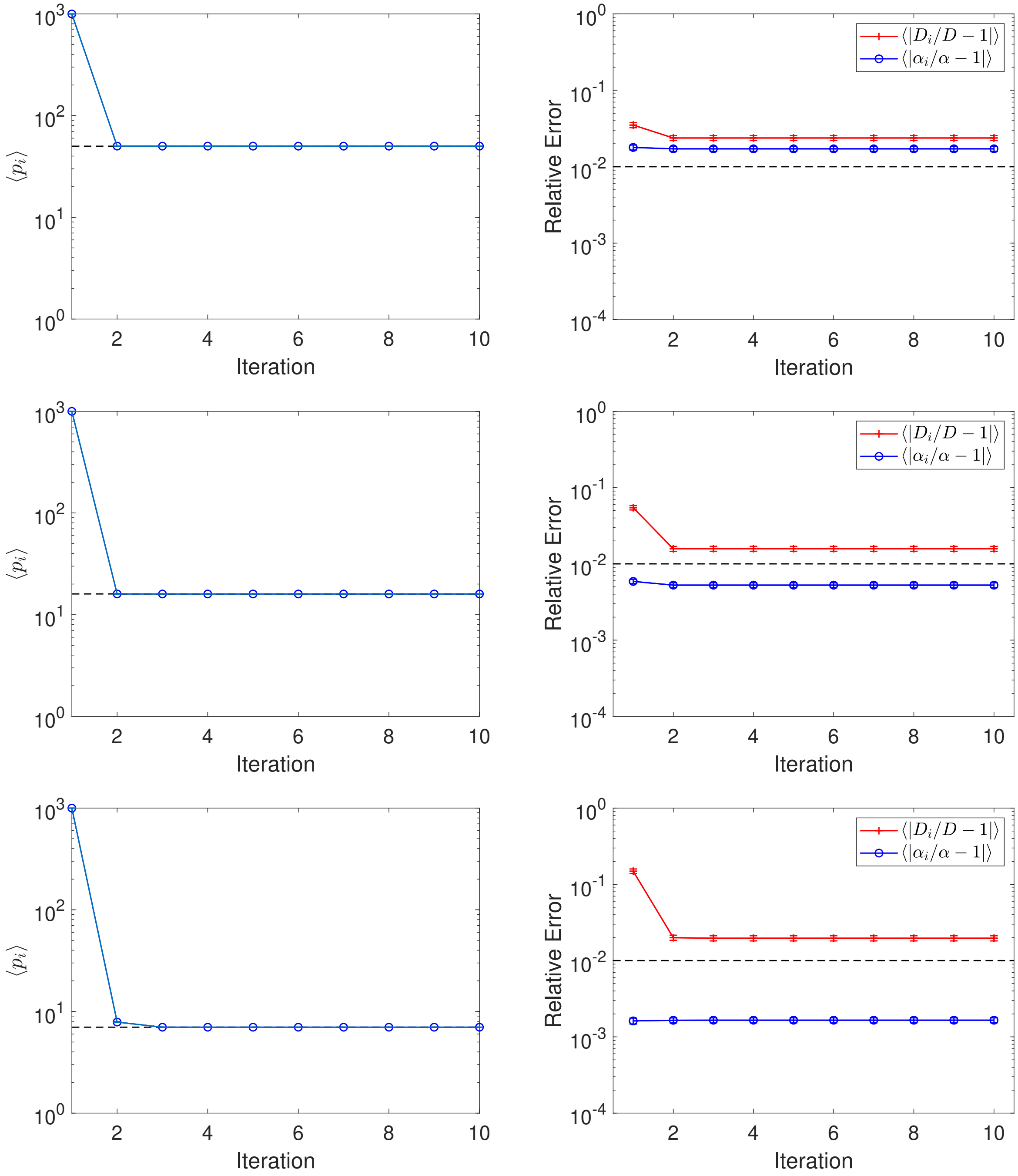

Figure 20: A plot of the value of $\langle p_i \rangle$ for each iteration with standard error bars (left plot in each row), along with a plot of the value of $\langle |D_i/D-1| \rangle$ (+ red crosses) and $\langle |\alpha_i/\alpha-1| \rangle$ (○ blue circles) for each iteration with standard error bars (right plot in each row). The simulated values include motion blur. These experiments were for $D = 2\,\mu m^2/s$, $\alpha = 1\,\mu m/s$, $\eta = 2\,\mu m$, $N_S = 10$ and $N = 1000$. The dashed line in each left plot corresponds to $p_{opt} = 50$ (top), $p_{opt} = 16$ (middle), and $p_{opt} = 7$ (bottom), while the dashed line in each right plot corresponds with the value 10^{-2}, indicating a 1% error. These correspond with $\Delta t = 1\,s$ (top), $\Delta t = 10\,s$ (middle) and $\Delta t = 100\,s$ (bottom).

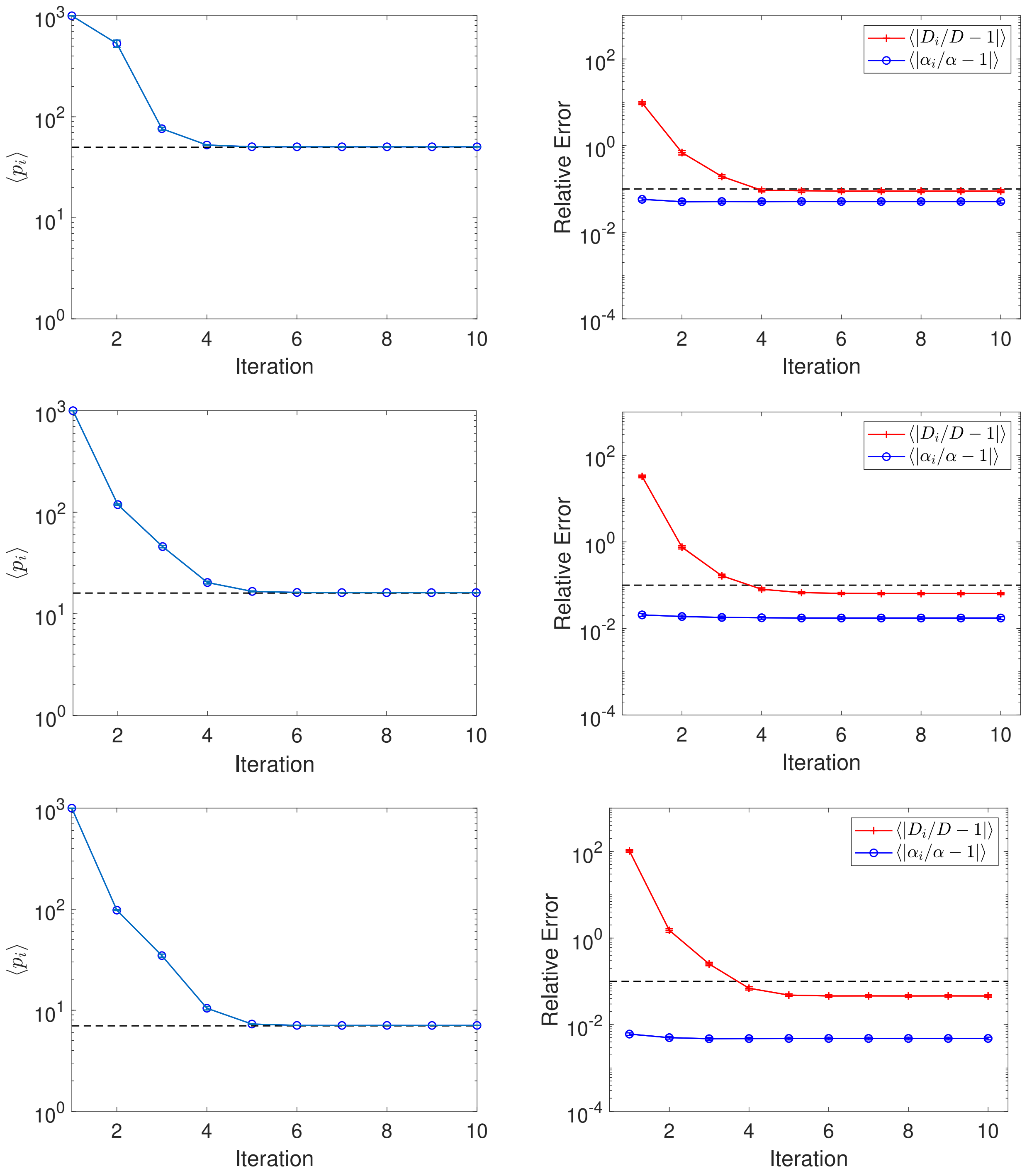

Figure 21: A plot of the value of $\langle p_i \rangle$ for each iteration with standard error bars (left plot in each row), along with a plot of the value of $\langle |D_i/D-1| \rangle$ (+ red crosses) and $\langle |\alpha_i/\alpha-1| \rangle$ (○ blue circles) for each iteration with standard error bars (right plot in each row). These experiments were for $D = 2\,\mu m^2/s$, $\alpha = 1\,\mu m/s$, $\eta = 2\,\mu m$, $N_S = 1$ and $N = 1000$. The dashed line in each left plot corresponds to $p_{opt} = 50$ (top), $p_{opt} = 16$ (middle), and $p_{opt} = 7$ (bottom), while the dashed line in each right plot corresponds with the value 10^{-1}, indicating a 10% error. These correspond with $\Delta t = 1\,s$ (top), $\Delta t = 10\,s$ (middle) and $\Delta t = 100\,s$ (bottom).

7 Determination of the drift direction

Until now we have assumed that the main interest is the determination of the magnitude of the drift velocity as well as the diffusion coefficient. If the drift angle is also of interest then this can also be inferred using the trajectory data. To calculate the mean direction and measures of the spread about the mean requires the use of circular statistics, details of which can be found in, for example, Fisher [?] or Mardia and Jupp [?]. Following in the same vain as the estimation of the MSD, we first calculate the ensemble overlapping time-averaged quantities

$$C_n = \sum_{j=1}^{N_S} \sum_{i=1}^{N+1-n} \cos\, \theta_{i,n}^{(j)} \quad \text{and} \quad S_n = \sum_{j=1}^{N_S} \sum_{i=1}^{N+1-n} \sin\, \theta_{i,n}^{(j)}, \tag{16}$$

where

$$\cos\, \theta_{i,n}^{(j)} = \frac{x_{i+n}^{(j)} - x_i^{(j)}}{|\boldsymbol{x}_{i+n}^{(j)} - \boldsymbol{x}_i^{(j)}|} \quad \text{and} \quad \sin\, \theta_{i,n}^{(j)} = \frac{y_{i+n}^{(j)} - y_i^{(j)}}{|\boldsymbol{x}_{i+n}^{(j)} - \boldsymbol{x}_i^{(j)}|}. \tag{17}$$

The resultant vector using data with a time lag of $n\Delta t$ is $R_n = \sqrt{C_n^2 + S_n^2}$ from which we can define the cosine and sine of the average angle using a time lag of n by

$$\cos \overline{\theta}_{d,n} = C_n / R_n \quad \text{and} \quad \sin \overline{\theta}_{d,n} = S_n / R_n. \tag{18}$$

The average angle using displacements with a time lag of $n\Delta t$ is given by

$$\overline{\theta}_{d,n} = \begin{cases} \tan^{-1}(S_n/C_n) & S_n > 0,\ C_n > 0 \\ \tan^{-1}(S_n/C_n) + \pi & C_n > 0 \\ \tan^{-1}(S_n/C_n) + 2\pi & S_n < 0,\ C_n > 0. \end{cases} \tag{19}$$

To help quantify the uncertainty in inferring the drift angle, the circular variance V_n is defined as

$$V_n = 1 - \overline{R}_n \quad \text{where} \quad \overline{R}_n = R_n / (N_S(N + 1 - n))$$

is the mean resultant length. Finally, the first and second central trigonometric moments

$$m_{1,n} = \frac{1}{N_S(N+1-n)} \sum_{j=1}^{N_S} \sum_{i=1}^{N+1-n} \cos\, (\theta_{i,n}^{(j)} - \overline{\theta}_n),$$

and

$$m_{2,n} = \frac{1}{N_S(N+1-n)} \sum_{j=1}^{N_S} \sum_{i=1}^{N+1-n} \cos\, (2(\theta_{i,n}^{(j)} - \overline{\theta}_n)),$$

can be used to define the sample circular dispersion

$$\hat{\delta}_n = (1 - m_{2,n})(2m_{1,n}^2).$$

The error bars in the following plots use the circular standard error

$$\hat{\sigma}_n = \sqrt{\hat{\delta}_n / (N_S(N + 1 - n))}.$$

Figure 22 shows the calculated circular variance and mean drift angle using $N_S = 10$ particles and $N = 100$ measurements, where we have assumed that the optimal measurement time has

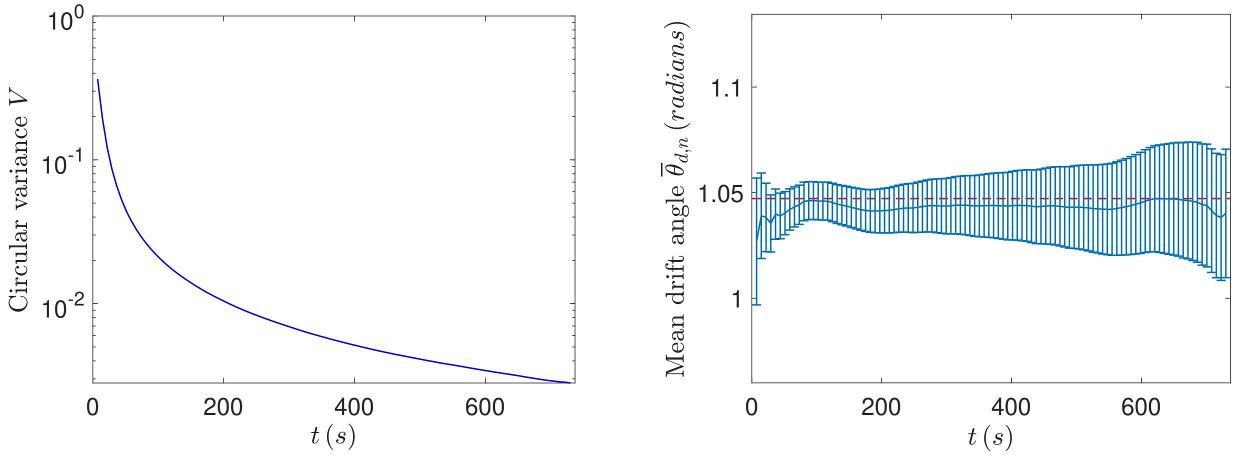

Figure 22: A plot of the circular variance V (left) and the mean drift angle $\overline{\theta}_{d,n}$ with standard error bars (right) for $D = 2\,\mu m^2/s$, $\alpha = 1\,\mu m/s$, $\eta = 2\,\mu m$, $\theta_d = \pi/3$ (1.047) $radians$, $N_S = 10$ and $N = 100$.

been used. We can see that the variance in the estimation of the drift angle decreases rapidly over time. This would suggest that the optimal time to infer the drift angle would be at the maximal measurement time. However, due to the lack of samples in the time averaging using the large time lags we find that there is very little difference in the quality of the inferred drift angle other than when very short lags are used. We leave till further study a theoretical analysis to determine the optimal strategy of the drift direction in combination with the other parameters of the drift-diffusion model.
